# Validation of upper thermal thresholds for outdoor sports using thermal physiology modelling

**DOI:** 10.1080/23328940.2023.2210477

**Published:** 2023-05-14

**Authors:** Takahiro Oyama, Minoru Fujii, Kenichi Nakajima, Jun’ya Takakura, Yasuaki Hijioka

**Affiliations:** a Department of Environment Systems, Graduate School of Frontier Sciences, The University of Tokyo, Kashiwa, Japan; b Center for Climate Change Adaptation, National Institute for Environmental Studies, Tsukuba, Japan; c Social Systems Division, National Institute for Environmental Studies, Tsukuba, Japan; d Material Cycles Division, National Institute for Environmental Studies, Tsukuba, Japan

**Keywords:** Body temperature, Computer simulation, Heat stroke, Practice guidelines, Safety, Sports, Thermal physiology modeling

## Abstract

Thermal safety guidelines with upper thresholds aim to protect athletes’ health, yet evidence-based sport-specific thresholds remain unestablished. Experimenting with athletes in severely hot conditions raises ethical concerns, so we used a thermo-physiological model to validate the thresholds of guidelines for outdoor sports. First, the reproducibility of the joint system thermoregulation model (JOS-3) of core temperature has been validated for 18 sports experiments (*n* = 213) and 11 general exercise experiments (*n* = 121) using the Bland – Altman analysis. Then, core temperatures were predicted using the JOS-3 in conditions corresponding to the upper thresholds, and if the 90^th^–99.7^th^ percentile core temperature value (corresponding to 0.3%–10% of the participants) exceeded 40°C, the thresholds were judged as potentially hazardous. Finally, we proposed revisions for sports with potentially hazardous thresholds. As a result, the JOS-3 could simulate core temperature increases in most experiments (27/29) for six sports and general exercises with an accuracy of 0.5°C. The current upper thresholds for marathons, triathlons, and football are potentially hazardous. Suggested revisions, based on specified percentiles, include: Football: revise from wet bulb globe temperature (WBGT) 32°C to 29–31°C or not revise. Marathon: revise from WBGT 28°C to 24–27°C. Triathlon: revise from WBGT 32.2°C to 23–26°C. If conducting sports events under the revised upper thresholds proves difficult, taking measures for a possible high incidence of heat illness becomes crucial, such as placing additional medical resources, assisting heat acclimatization and cooling strategies for participants, and rule changes such as shorter match times and increased breaks.

## Introduction

Outdoor sports competitors, who may perform strenuous exercise in severe heat environments, are among the most susceptible to heat stress. Recently, extreme heat has frequently caused large changes in sporting events such as the Summer Olympics, the 2019 World Athletics marathon, and the 2014 Tennis Australian Open [1]. The International Olympic Committee (2016) stated that safety requirements must be defined and applied in each sport to protect the health of participants and the public, and referred to environmental conditions such as heat [2]. Thermal safety guidelines (hereinafter referred to as guidelines) with upper thresholds have been developed by many organizations, such as international sports federations. However, the thresholds can still be validated for a couple of reasons. First, evidence-based sport-specific thresholds have not yet been established [3,4]. Second, there are cases where the current upper thresholds are either too liberal [5] or conservative [6]. It is ethically questionable to conduct experimental studies where athletes perform various outdoor sports in an upper threshold environment to study the risk of heat illnesses [7]. Conversely, computer body temperature simulations could provide insight into the heat illness risk under various climate conditions without imposing a health burden on athletes [8]. Therefore, here we validate the upper thresholds of guidelines for outdoor sports by estimating the risk of heat illnesses using a thermo-physiological model.

## Methods

### Sports selection

To select sports with many competitors, i.e. potential heat illness patients, we initially targeted the 21 outdoor sports of the 2020 Tokyo Olympics. These represent the current sports world for two reasons: (1) Many were already “widely practiced by men in at least seventy-five countries and on four continents, and by women in at least forty countries and on three continents” when they were adopted for the 2004 Athens Olympics [9]; (2) More recently added sports (e.g. skateboarding) were adopted because of their global popularity [10]. We identified the sports with thermal threshold guidelines through a survey of the international sports federations and other relevant organizations’ websites.

Among the sports identified, we selected those for which the measured core temperature during exercise, as well as the minimum parameters for simulating core temperature were specified in the literature. For each sport, we searched the full literature text in English using ”(*name of sports*) AND ([rectal temp*] OR [core temp*])” on UTokyo REsource Explorer (TREE, https://tokyo.summon.serialssolutions.com), then used the top 50 pieces of literature. TREE provides access to more than 120 million scholarly and peer-reviewed articles (as of March 2023) from multiple major databases such as Web of Science, Scopus, and EBSCOhost. This allows for easy access to a wide range of pertinent literature. The minimum parameters were those related to the environment (ambient temperature [T_a_], relative humidity [RH]), and exercise (type and duration) and individual factors (height, weight, age) for heat illnesses. We retrieved and surveyed 260 articles relevant to the six sports selected for this study. We initially narrowed the list down to 62 articles by screening titles and abstracts, and subsequently, to 12 by screening the full text. A summary of the literature review on the six sports is presented in Table 1.

**Table 1.** Summary of literature review on six sports.

Sport	Marathon	Football	Rowing	Rugby	Tennis	Triathlon	Total (6 sports)
Search words	(marathon) AND ((rectal temp*) OR (core temp*)) NOT (TitleCombined:(swim*)) NOT (TitleCombined:(ultra*)) NOT (TitleCombined:(ironman))	((football) OR (soccer)) AND ((rectal temp*) OR (core temp*))	(rowing) AND ((rectal temp*) OR (core temp*))	(rugby) AND ((rectal temp*) OR (core temp*))	(tennis) AND ((rectal temp*) OR (core temp*))	(triathlon) AND ((rectal temp*) OR (core temp*))	N/A
Search condition 1	Scholarly & Peer-Reviewed	Scholarly & Peer-Reviewed	Scholarly & Peer-Reviewed	Scholarly & Peer-Reviewed	Scholarly & Peer-Reviewed	Scholarly & Peer-Reviewed	N/A
Search condition 2	Full Text Online	Full Text Online	Full Text Online	Full Text Online	Full Text Online	Full Text Online	N/A
Search condition 3	English	English	English	English	English	English	N/A
Search date	October 31st, 2022	October 31st, 2022	October 31st, 2022	October 31st, 2022	October 31st, 2022	October 31st, 2022	N/A
# of references found	138	305	22	137	93	38	733
# of references for which the title and abstracts were reviewed	50	50	22	50	50	38	260
# of references for which the full text was reviewed	19	5	4	4	23	7	62
# of references identified in the review above	3	2	1	1	3	1	11
# of references identified from literature review of other sports	0	1	0	0	0	0	1
# of references identified for the sport(s)	3	3	1	1	3	1	12

### Validation of the thermal physiology model accuracy in simulating core temperature

Among the most recognized models [11], we selected Tanabe (2002) because its latest model (the joint system thermoregulation model [JOS-3] [12]) can set parameters according to target sports (e.g. high metabolic rate, high wind speed) and because the calculation process is transparent since the source code is publicly available. Other models were not considered to be able to perform arbitrarily parameterized calculations under the high metabolic and wind speed conditions required in this study. JOS-3 was developed to predict human physiological responses while taking personal characteristics in transient and non-uniform thermal environments into consideration [12]. It consists of 85 nodes, which are specific points within the JOS-3 that represent different parts of the human body (the central blood node, 17 artery nodes, 17 vein nodes, 12 superficial vein nodes, 17 core nodes, 2 muscle nodes, 2 fat nodes, and 17 skin nodes) and the entire body is divided into the following 17 segments: head, neck, chest, back, pelvis, left (L)-shoulder, L-arm, L-hand, right (R)-shoulder, R-arm, R-hand, L-thigh, L-leg, L-foot, R-thigh, R-leg, and R-foot [12]. Similar to many other models, JOS-3 simulates the heat balance of the human body by modeling phenomena, such as heat production, vasodilation, vasoconstriction, and heating. JOS-3 is unique as it also takes the arteriovenous anastomosis blood flow, non-shivering thermogenesis, and age effect on heat production and thermoregulation into account [12].

JOS-3 was validated in low-metabolism indoor settings [12] and Stolwijk’s model, on which JOS-3 is based, has demonstrated high accuracy in reproducing body temperature during intense exercise with a metabolic rate exceeding 8 met in a hot environment (Ta 30°C, RH 30%, wind speed 0.1 m/s) [13]. However, JOS-3’s applicability to high-metabolism outdoor sports is unknown. Thus, we validated the model’s accuracy in simulating the core temperature of sampled persons in sports experiments by simulating either games in fields or chambers (*n* = 213) or general exercise experiments in chambers (*n* = 121). Most of the exercise experiments included subjects in their 20s and 30s with relatively high aerobic capacity (maximal oxygen uptake>50 mL.kg-1.min-1). They were approximately 1.6–1.7 m in height and 60–70 kg in weight. These participants exercised for more than 1 hour in a hot and humid environment with ambient temperature above 30°C, relative humidity above 50%, and light wind. In all but one exercise experiment, either heat acclimation was not implemented, or the status of heat acclimation was unknown. The conditions of each exercise experiment are listed in Supplementary Tables 1 and 3. We have drawn the measured and simulated core temperature values on a Bland – Altman plot [14] and determined whether they fall within±0.5°C of the corrected mean for the systematic error (bias). The criterion of ±0.5°C was set with reference to the accepted criterion for noninvasive core temperature measurement [15], which is the root mean squared error<0.5°C. If parameters of JOS-3 other than the minimally required ones are unknown, we set them according to the assumptions in Table 2.

**Table 2.** Settings for unknown parameters.

Parameters		Settings for unknown parameters
Environmental factors	Mean radiant temperature	Ambient temperature [T_a_] +20°C (for outdoor daytime) or the same as T_a_ (for indoor/outdoor nighttime)
	Wind speed	Three patterns of 0, 1.0, and 3.0 m/s (outdoor) or 0 (indoor)
	Trend of T_a_	Three constant patterns at average T_a_, low (average – standard deviation [SD]) in the first half and high (average + SD) in the second half, high (average + SD) in the first half of the game and low (average – SD) in the second half of the game.
Exercise factors	Metabolic rate	In running-type exercises, American College of Sports Medicine (ACSM) formula was used [16]. If the SD of the running speed was unknown, 7% [16] of the average metabolic rate was considered the SD. For other types of exercise, metabolic rate was set referring to the typical METs values by Ainsworth et al. (2011) [17] and adjusted them as necessary; 10% of the average metabolic rate was considered the SD.
	Duration	For marathons and triathlons, where metabolic rate and duration are considered related, the assumption is that if the metabolic rate is high (average + SD), the duration is short (average – SD), and if the metabolic rate is low (average – SD), the duration is prolonged (average + SD).

For height, weight, and age (individual factors), metabolic rate and duration (exercise factors), three patterns of mean and mean±standard deviation (SD) were set, respectively, and for the T_a_ trend (environmental factors), the above three patterns were set and combined using an exhaustive approach to generate all possible combinations, resulting in a total of 243 patterns. However, if the SD was unknown for a parameter, no SD pattern was set, and 81 patterns were calculated for certain experiments. Metabolic rate and duration were assumed to be linked as described in Table 2. If the experiment was carried out outdoors and the wind speed was unknown, 729 patterns of calculations were carried out, with three wind speeds of 0, 1.0, and 3.0 m/s. The number of patterns for 29 experiments is given in the Supplementary information. Shortwave solar radiation was not considered because there is currently insufficient pertinent human experimental data to validate the JOS-3 [12].

### Validation of upper thermal thresholds

First, for each sport, there are three or four different patterns of environmental factors (T_a_, RH) based on the upper thresholds of the guidelines. Then, as in the previous section, 81 patterns consisting of individual factors (height, weight, age) and exercise factors (metabolic rate, duration of sport) were generated. Using an exhaustive approach to combine all possible combinations of these patterns with the patterns of environmental factors, we predicted core temperature, resulting in a total of 243 or 729 patterns for each sport. For sports where WBGT dictated the upper threshold, T_a_ and RH were estimated from the Korey Stringer Institute table [18] for three patterns: high temperature and low humidity (RH 20%), medium temperature and medium humidity (RH 50%), and low temperature and high humidity (RH 80%). For sports where the upper threshold was set only by T_a_, three patterns were set for the same temperature: low (RH 20%), medium (RH 50%), and high humidity (RH 80%). For rugby, where the Heat Stress Index dictated the upper threshold, four temperature and humidity patterns were set according to the World Rugby guideline [19]. Ambient wind speed was set at 1.0 m/s for all sports. The main parameter settings in JOS-3 for the six target sports are shown in Supplementary Table 5.

Secondly, if the bias-corrected 90^th^, 95^th^, 97.5^th^, 99^th^, or 99.7^th^ percentile (PCTL) values of predicted core temperatures exceeded 40°C, a diagnostic criterion for exertional heat stroke (EHS), we judged the upper threshold as potentially hazardous. The manageable incidence of heat illness at each sporting event varies depending on the medical resources available in each region and during each event. For example, mass casualty incidents (events that produce more patients than the number that can be managed safely using routine procedures and available resources such as ambulances, emergency room beds, emergency room staffing) occurred at the Twin Cities Marathon (Minneapolis-St. Paul, MN) during the years 1997–2008 in races where the rate of unsuccessful starters per finisher exceeded 0.12–0.13 [5]. Therefore, we present multiple PCTL values corresponding to several incidences of heat illness. The 99.7^th^ PCTL value corresponds to the exertional heat illness incidence rate of 2.97/1000 finishers in the Falmouth Road Race (11.26 km, FRR) in the USA [20]. The race has a 100% survival rate for EHS patients due to effective first aid measures, such as cold water immersion [21], but as such events are rare, the incidence rate is considered to be the upper limit for many events. For sporting events with smaller participants, 90^th^, 95^th^, 97.5^th^, and 99^th^ PCTL values are also referenced. We set a limit of 40°C for core temperature, that of 40–41°C is included in multiple diagnostic criteria for exertional heat stroke [22], and the prognosis worsens with longer >40°C core temperature duration [23]. The tolerance to elevated core temperature varies greatly among athletes. Generally, higher aerobic capacity is associated with higher core temperatures at which exhaustion is reached [24]. Some athletes have been reported to not experience any clinical sequelae even when their core temperature well exceeds 40°C [7]. Therefore, we conducted sensitivity analyses for different core temperature limits (39.4°C, 41°C) [22] and prolonged duration of core temperature>40°C (30 min).

Finally, for the potentially hazardous thresholds, we propose revising the thresholds according to our calculations for safety.

## Statistical analysis

### Validation of the thermal physiology model accuracy in simulating core temperature

After confirming that the difference between the measured and simulated mean core temperatures followed a normal distribution using the Shapiro – Wilk test, the Bland – Altman analysis was carried out to determine the model accuracy. The 95% confidence intervals for bias and the upper and lower limits of agreement were calculated by (1), (2), and (3), respectively, according to Bland and Altman (1986) [14] and the CHAMP statement [25].

(1) $$95\%\;\mathrm{CIs}\;\mathrm{of}\;\mathrm{Bias}:\mathrm{Bias}\pm t_{n-1}\times \sqrt{\frac{SD^{2}}{n}}$$

(2) $$95\%\;\mathrm{CIs}\;\mathrm{of}\;\mathrm{the}\;\mathrm{upper}\;\mathrm{limits}\;\mathrm{of}\;\mathrm{LOA}:\mathrm{Bias}+2\mathrm{SD}\pm t_{n-1}\times \sqrt{\frac{3SD^{2}}{n}}$$

(3) $$95\%\;\mathrm{CIs}\;\mathrm{of}\;\mathrm{the}\;\mathrm{lower}\;\mathrm{limits}\;\mathrm{of}\;\mathrm{LOA}:\mathrm{Bias}-2\mathrm{SD}\pm t_{n-1}\times \sqrt{\frac{3SD^{2}}{n}}$$

**Table:** 

Abbreviations in equations CIs, confidence intervals; LOA, limits of agreement; n, sample size; SD, standard deviation; t_n-1_, t-value for a two-sided probability of 5% in a t-distribution with *n*-1 degrees of freedom

## Other

The significance level was set at *p* = 0.05. Statistical analyses were performed in Python 3.9.7 on a Windows 10 Pro machine.

## Results

### Sports selection

Guidelines with thermal thresholds were confirmed for nine of the 21 outdoor sports of the 2020 Tokyo Olympics (athletics [26,27], beach volleyball [28], field hockey [29], football [30], golf [31], rowing [32], rugby seventh [19], tennis [33], and triathlon [34]). Most sports adopted WBGT as an indicator in their guidelines (six; in rowing, WBGT is used in combination with T_a_), with an upper threshold ranging 28–32.2°C. Other indicators were T_a_ (three sports), Heat Stress Index (one sport) and apparent temperature (one sport, as an alternative to WBGT). In six of the above nine sports, core temperatures and the parameters necessary for the simulations were found in the literature (athletics [marathon] [35–37], football [38–40], rowing [41], rugby seventh [42], tennis [43–45], and triathlon [46]). Thus, we verified the guideline validity for these six sports. The overall results are presented in Table 3.

**Table 3.** Sport selection results.

Outdoor sports in Tokyo Olympics 2020	Thermal safety guidelines with upper thresholds			References with core temperature and calculation parameters (number of references and sample size [n])	Selected sports
	Guidelines identified	Index (upper threshold)	Author(s) (*other than IFs)		
Archery	F	N/A	N/A	N/A	F
Athletics (marathon)	T	WBGT (28°C)	ACSM*, IIRM*	T (3, *n* = 99)	T
Baseball/softball	F	N/A	N/A	N/A	F
Beach volleyball	T	WBGT (31°C)	FIVB	F	F
Canoe	F	N/A	N/A	N/A	F
Cycling (mountain bike, road, BMX)	F	N/A	N/A	N/A	F
Equestrianism	F	N/A	N/A	N/A	F
Field hockey	T	T_a_ (42°C)/ RH (75%)	FIH	F	F
Football	T	WBGT (32°C)	FIFA	T (3, *n* = 38)	T
Golf	T	T_a_ (37°C)	IGF	F	F
Modern pentathlon	F	N/A	N/A	N/A	F
Rowing	T	T_a_ (38°C)/ WBGT (32°C)	FISA	T (1, *n* = 4)	T
Rugby seventh	T	HIS (150)	World Rugby	T (1, *n* = 22)	T
Tennis	T	WBGT (32.2°C)/ T_ap_ (34°C)	ITF	T (3, *n* = 25)	T
Triathlon	T	WBGT (32.2°C)	World Triathlon	T (1, *n* = 14)	T
Sailing	F	N/A	N/A	N/A	F
Shooting	F	N/A	N/A	N/A	F
Skateboarding	F	N/A	N/A	N/A	F
Sport climbing	F	N/A	N/A	N/A	F
Surfing	F	N/A	N/A	N/A	F
3×3 basketball	F	N/A	N/A	N/A	F

Abbreviations: ACSM, American College of Sports Medicine; FIFA, International Federation of Association Football; FIH, International Hockey Federation; FIVB, International Volleyball Federation; HSI, Heat Stress Index; IGF, International Golf Federation; Ifs, international sports federations; ITF, International Tennis Federation; IIRM, International Institute for Running Medicine; T_a_, ambient temperature; UIPM, Union Internationale de Pentathlon Moderne; WBGT, Wet Bulb Globe Temperature; T, True; F, False.

### Validation of the thermal physiology model accuracy in simulating core temperature

**Figure 1.**
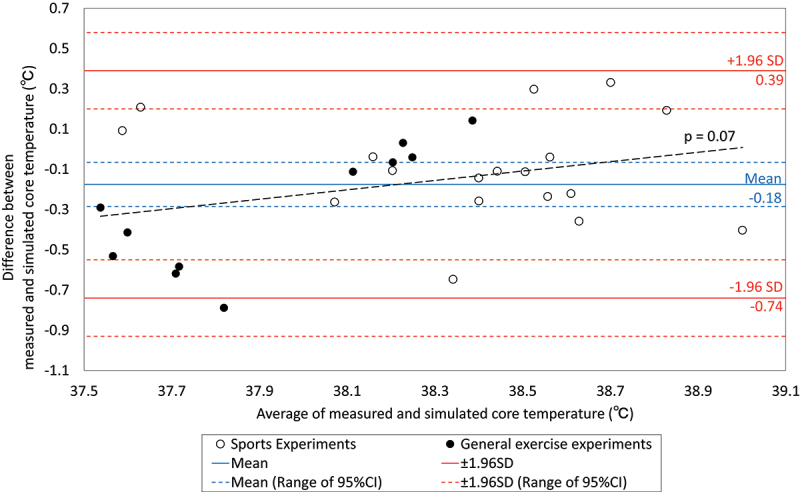
Bland-Altman plot of measured and simulated core temperature in 29 experiments: 11 for general exercise experiments and 18 for six selected sports.

Abbreviations: CI, confidence interval; SD, standard deviation

We validated the accuracy of the JOS-3 in simulating core temperature in 29 experiments: 11 for general exercises in chambers [47–49] and 18 for six sports confirmed in the previous section, using Bland – Altman analysis. The difference between the measured and simulated temperatures averaged over the number of measurements for each case followed a normal distribution (*p* = 0.77). Figure 1 shows an average bias of 0.18°C higher for the simulated values, with 95% limits of agreement (mean±1.96 SD) ranging from −0.74 to 0.39°C. No proportional relationship was identified (*p* = 0.07). As all but two experiments (one of laboratory exercise and one of football) fell within a range of±0.5°C, around −0.18°C, it is judged that the JOS-3 model can be applied to validate the upper guideline thresholds if the settings correspond to the conditions of the experiments that met the accuracy requirements. The results and parameter settings for 29 experiments are shown in the Supplementary information (Supplementary Table 6 -34 , Supplementary Fig. 1 -51). Simulated sweat loss in JOS-3 tended to be less than measured values (Supplementary Fig. 41–51).

### Validation of upper thermal thresholds

**Figure 2.**
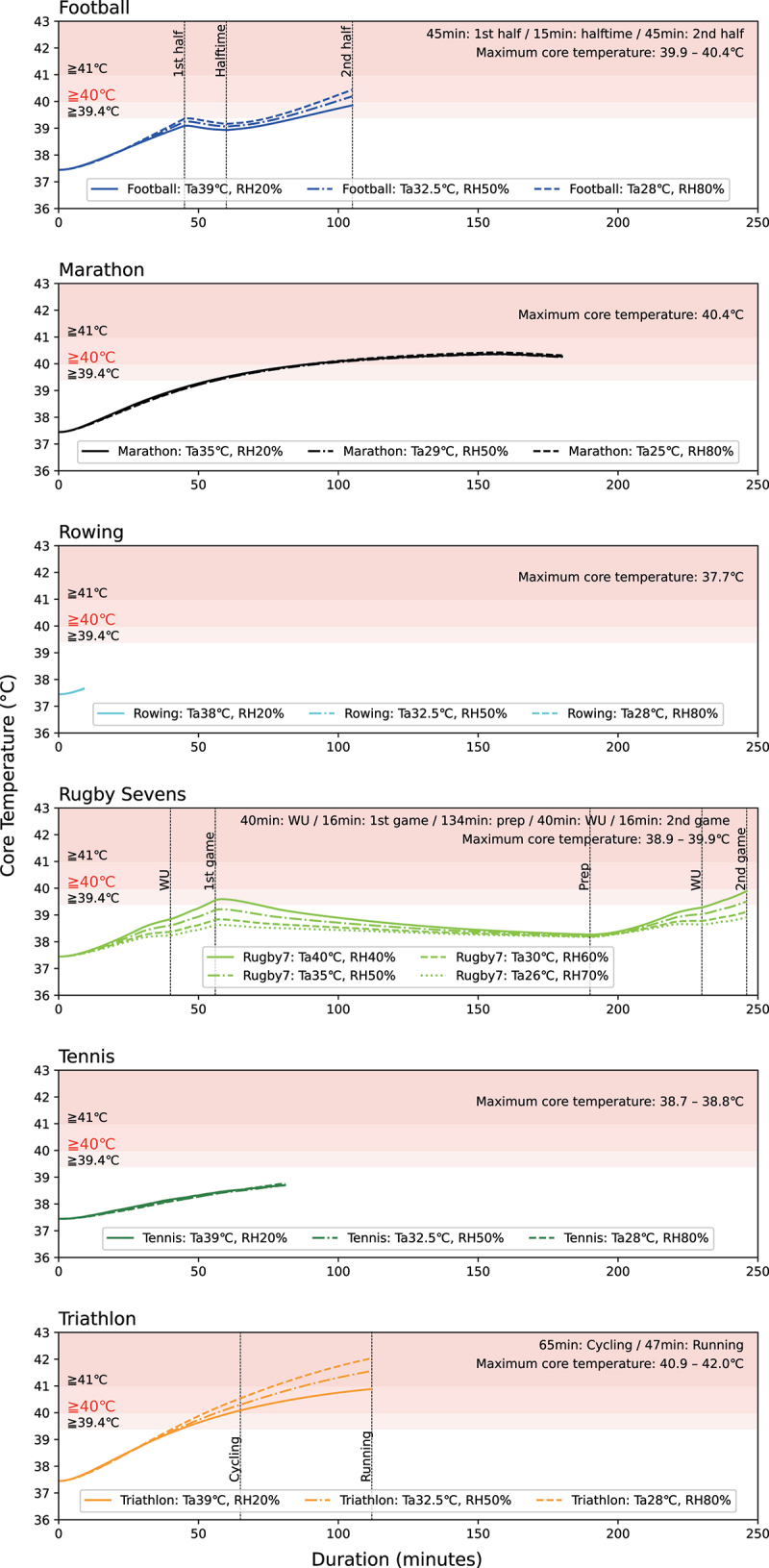
Predicted core temperature in six sports at the upper thresholds of the thermal safety guidelines (99.7^th^ percentile).

Abbreviations: prep, preparation; RH: relative humidity; T_a_: ambient temperature; WU: warm-up

The potential hazard of the upper thermal thresholds was validated according to whether the 90^th^, 95^th^, 97.5^th^, 99^th^, and 99.7^th^ PCTL values of predicted core temperature, corrected for the systematic error from measurements (−0.18°C) exceeded the 40°C limit. As the trend of the results is similar for any PCTL, the results for the 99.7^th^ PCTL are explained in the text, while the results for the other PCTL are in Supplementary Fig. 52–55. The result (Figure 2) shows a significant difference in predicted peak core temperature from below 39°C to above 42°C among the six sports. For football, marathons, and triathlons, the core temperature exceeded 40°C in all calculations except in football. This is particularly true for triathlons, where core temperatures above 41°C were predicted.

Sensitivity analyses were performed for different core temperature limits (39.4°C, 41°C) and the duration of core temperature above 40°C (30 min).

- Core temperature: When the limit for core temperature was set at 41°C, the core temperature fell below the limit in marathons and football, but still exceeded in two calculations in triathlons. When the core temperature limit was set at 39.4°C, core temperatures exceeded the limit in marathons, football, and triathlons, as well as in one out of four calculations of Rugby sevens.
- Duration of core temperature above 40°C: If the duration of core temperature above 40°C is allowed up to 30 minutes [23], assuming a situation where rapid cooling of the core temperature can be carried out, then elevated core temperature over time was acceptable in football, but still unacceptable in all triathlon and marathon calculations.

**Figure 3.**
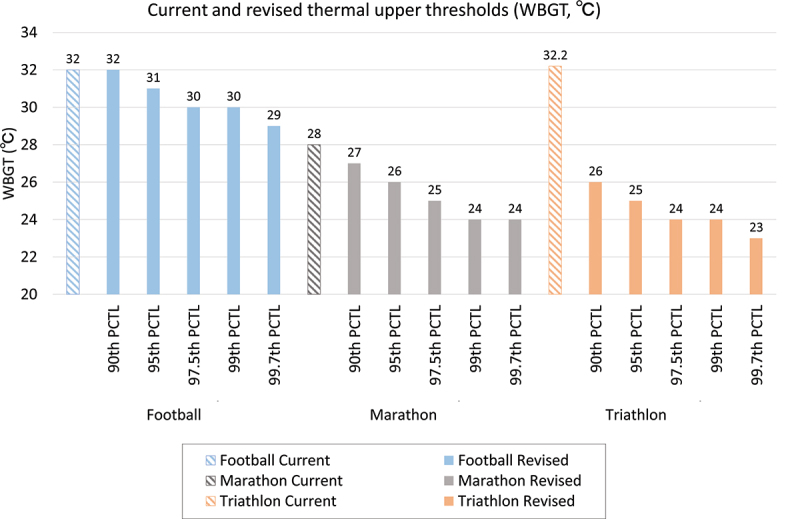
Current and revised upper thermal thresholds for football, marathons, and triathlons.

Abbreviations: PCTL, percentile; WBGT, Wet Bulb Globe Temperature

Based on the above set-up, to ensure that the 90/95/97.5/99/99.7th PCTL values for core temperature did not exceed 40°C while retaining the parameter settings (other than the environmental factors), the upper thresholds for football could be revised from WBGT 32°C to 29–31°C or not be revised, for marathons from WBGT 28°C to 24–27°C, and for triathlons from WBGT 32.2°C to 23–26°C (Figure 3). The changes in the parameter settings of the three sports in JOS-3 and predicted core temperatures are shown in Supplementary Table 35–39 and Supplementary Fig. 56–60. Supplementary Table 40 demonstrates the PCTLs corresponding to a core temperature of 40°C, stratified according to WBGT and sport.

## Discussion

### Study findings

The first finding of this study are the validation results of the upper thermal thresholds for currently representative outdoor sports. We found relative heat illness risks under the upper thresholds for the six sports and pointed out that the thresholds for the marathons, football, and triathlons are potentially hazardous. In particular, in the triathlons and marathons, with predicted core temperatures above 40°C for more than 30 minutes in many calculations, and peak core temperatures above 41°C in the triathlon, the current upper thresholds may be putting runners at high risk of heat illness (Figure 2). The potential hazard of the current upper threshold for marathons identified is consistent with previous findings [5] that the current guideline may be “too liberal” and for races at latitudes equal to or above 40°N, nonelite, non-acclimatized participants should consider not starting if the WBGT is above 20 to 21°C. The current upper threshold for triathlons, WBGT 32.2°C, was initially recommended for “Training and noncontinuous activity” in the American College of Sports Medicine’s Position Stand [7]. In the same position stand, an upper threshold of 27.9°C was recommended for “Continuous Activity and Competition” [7]. Although not currently applied, the latter threshold could be more appropriate for endurance and competitive nature of triathlon events. Relatedly, Triathlon Australia has set WBGT of 30°C as the threshold for postponement or cancellation of events (except for swimming) and 26°C for limit intensity and duration to less than 60 min per session [34]. In addition, 15 competitors (0.8% of all competitors) experienced heat-related collapses during fun or sprint distance triathlons in Melbourne due to insufficient heat acclimatization, even though the WBGT during the race was between 20 and 27°C [50]. These cases support this study’s proposal to lower the thresholds for triathlons. There have been no recorded deaths from exertional heat stroke among elite football players during competition matches [51]. However, exertional heat illness is common in both youth (ages 9–19) and college football in the United States [52,53]. Many of these episodes of heat illness occur in areas above 40°N [54], which rarely reach a WBGT of 32°C even during summer days [52]. Therefore, reducing the current threshold WBGT of 32.2°C could be considered for football. Under the present conditions, the upper thresholds could be revised from WBGT 32°C to 29–31°C or not be revised for football, from WBGT 28°C to 24–27°C for marathons, and from WBGT 32.2°C to 23–26°C for the triathlon, depending on the manageable incidence of heat illness (Figure 3).

The second finding is that the thermo-physiological modeling approach can be applied to heat illness risk assessments in various sports. The difference between the measured and calculated values is −0.18 ± 0.29°C, which is comparable to the difference (−0.1 ± 0.5°C) found when applying a different thermal physiology model to simple exercise [8]. Our thermo-physiological modeling approach avoids the logistical and ethical difficulties [7,8] of experimenting with athletes in a severely hot environment and may be an option for heat illness risk assessments for a wider variety of sports as well as environmental, exercise, and individual factors.

### Research and policy implications

For sport guideline validation, we predicted the core temperature using parameter settings based on selected literature, including core temperature during and after sport. Height, weight, age, and sex were assumed to be uniform in all sports. In reality, exercise and individual factors differ significantly between sports groups. Therefore, validating the applicability of our proposed method in each target group and considering threshold modification or creating new thresholds are important. Also, the authors recommend prioritizing the validation of thresholds for general sportspersons rather than for elite athletes. In large elite events, risk mitigation is carried out with a well-equipped medical staff, cold water immersion bathtubs [55,56], and some athletes can tolerate core temperatures of well above 40°C [7]. However, for general events, assuming the above conditions does not seem appropriate, and the prevention of heat illness relies more on the observation of guidelines.

International and national sports organizations should refer to this and future relevant research to consider adopting more conservative thresholds for safer heat illness prevention based on environment, exercise, individual factors, as well as organizational and local healthcare capacity for tolerating heat illness incidence increase. It is especially important to identify effective measures to avoid, whenever possible, cancellation of events in places that experience intense heat as it leads to lost opportunities for participation in sports. These may include measures that involve human factors, such as educational programs regarding heat acclimation and cooling strategies as well as rule adjustments, such as shortening the event duration, increasing breaks, and reducing exercise intensity. Other measures that can be implemented include changes to the external environment, such as changing the timing of the event, holding the event indoors, and improving medical facilities. Subsequently, the thresholds may change depending on the measures that can be implemented for each location and event. Furthermore, it is considered important to provide support, such as dissemination and training, in order for local sports organizations with limited resources to implement the guidelines.

The above studies, and the development and implementation of guidelines, could reduce the incidence of heat illnesses in a wide range of sports.

### Limitations

(1) Central nervous system abnormalities, which are included in the general EHS diagnostic criteria [22], are not simulated by current thermo-physiological models. However, considering that central nervous system abnormalities are triggered by an excessive increase in body temperature [57], it is reasonable to use core temperature to estimate EHS risk. (2) In the present study, the risk of heat illness was assessed during matches. When targeting practice sessions, the challenge will be establishing exercise factors, such as duration and metabolic rate, considering that various forms of exercise, such as conditioning, running, and strength training [17], can be included in the practice menu. (3) Shortwave solar radiation was not considered because there is insufficient human experimentation data to validate the JOS-3. Therefore, the applicability of this study is not clear in cases where there is a significant deviation from the solar radiation during outdoor daytime exercise experiments used to validate the accuracy of the model in this study, e.g. when the solar radiation is significantly intense. Some weather-related phenomena, such as sportsperson water adhesion due to rainy weather, were not considered too. The accuracy of core temperature may be further improved by implementing and validating a function to simulate more detailed weather conditions in thermo-physiological models. (4) Most of the exercise experiments used to verify the accuracy of JOS-3, as described in the Methods section, included subjects in their 20s-30s, who had relatively high endurance, average height and weight, and who exercised continuously for one hour or more in a hot and humid environment. Heat acclimation was either not implemented or the status of heat acclimation was unknown. Exposed surface area was also not considered in this study as data were not available for many exercise experiments. The ability to regulate body temperature varies with various factors including age [58], heat acclimation status [59], and disabilities such as spinal cord injury [60]. In the future, it is desirable to accumulate core temperature data during exercise under a wider range of conditions, such as those mentioned above, in order to verify the reproducibility of the JOS-3. We did not consider ethnic differences in thermal physiology as well, as the data used to validate the model did not include information on ethnicity. When targeting certain groups of people, minor adjustments in JOS-3, such as the proportions of body tissues, would result in higher accuracy. (5) The number of studies selected to validate the accuracy of JOS-3 is not large, as also demonstrated in Tables 1 and 3; 12 articles (18 experiments) were chosen for 6 sports. In the future, as more studies are published leading to a more comprehensive literature, the sample size would increase. Hence, it is possible that different results may be obtained using the same method and that more sports could be evaluated. (6) We could not confirm the compliance of thermal environment and thermal strain measurements with ISO 7726:1998 [61] and ISO 9886:2004 [62], as none of the selected studies included complete information on the accuracy and uniformity of ambient temperature measurement, core temperature measurement, rectal thermistor insertion depth, and calibration, as required by these standards. Only academic and peer-reviewed articles have been included in this study, and only rectal and intra-abdominal temperatures suitable for measurement during exercise have been considered as core temperatures. These should have contributed to the quality of the data. However, there may still be a certain amount of variability among the data collected. (7) This study did not consider the effects of cooling, such as water dousing, which is commonly used in marathons and triathlons. With the accumulation of physiological data such as core temperature when cooling is applied, as well as the addition of JOS-3 functionality, it could be possible to investigate the effects of cooling on core temperature and thresholds using the methods employed in this study. Cooling strategies such as ice slurry ingestion, cold water immersion, facial wind/water spray, and ice vest are particularly effective [63]. They are promising targets for consideration in the future, along with chronic heat acclimation strategies [64]. (8) Although the accuracy of JOS-3 in simulating core temperature during sports is acceptable when compared with the standard for noninvasive core temperature measurement (±0.5°C) [15], there are cases in which core temperature changes of 0.2°C or more are considered meaningful [65]. Therefore, it should be noted that the accuracy of the JOS-3 is insufficient to capture small changes in core temperature during sports. Improvement of the environmental and exercise input data, as well as the JOS-3 model itself, may improve the accuracy and expand the application of this research method. Enhancing the JOS-3‘s ability to accurately estimate sweat loss could potentially improve the accuracy of core temperature simulation.

In conclusion, the upper thermal thresholds of guidelines for marathons, triathlons, and football are potentially hazardous. The thresholds could be revised from WBGT 32°C to 29–31°C or not be revised for football, from WBGT 28°C to 24–27°C for marathons, and from WBGT 32.2°C to 23–26°C for triathlons, for participants’ safety. If conducting sports events under the revised upper thresholds proves difficult, taking measures for a possible high incidence of heat illness becomes crucial, such as placing additional medical resources, assisting heat acclimatization and cooling strategies for participants, and rule changes such as shorter match times and increased breaks. The thermo-physiological model approach can be applied to heat illness risk assessment in various sports and has the potential to contribute to such assessment for various sports, as well as environmental, exercise, and individual factors in the future.

## Supplementary Material

Supplemental Material

## Data Availability

The datasets generated and analyzed during the current study are available from the corresponding author upon reasonable request.

## Abbreviations

EHS: Exertional heat stroke
JOS-3: Joint system thermoregulation model
PCTL: Percentile
RH: Relative humidity
SD: Standard deviation
Ta: Ambient temperature
TREE: UTokyo REsource Explorer
WBGT: Wet bulb globe temperature
