# Supplementary material for: Validation of upper thermal thresholds for outdoor sports using thermal physiology modelling
Source: Temperature (Austin). 2023 May 14;11(1):92–106. doi: 10.1080/23328940.2023.2210477 (PMC10989705; doi:10.1080/23328940.2023.2210477)
Supplement: Supplemental Material [file KTMP_A_2210477_SM5261.docx]

Supplementary information for “Validation of upper thermal thresholds for outdoor sports using thermal physiology modelling”

1. Conditions of the general exercise experiments used to validate the accuracy of the joint system thermoregulation model [JOS-3]; Values represent means, with standard deviations appended where available.

| Experiments | Conditions | | | | | | | | | | | | |
| --- | --- | --- | --- | --- | --- | --- | --- | --- | --- | --- | --- | --- | --- |
|  | Environment | | | | Exercise | | Individual | | | | | | n |
|  | T_a_ (℃) | RH (%) | v (m/s) | Location | M (ml・kg^-1^・min^-1^) | D (min) | W (kg) | H (m) | Age (year) | Sex | VO_2max_ (ml・kg^-1^・min^-1^) | HA |  |
| 1: Ichinose-Kuwahara et al. (2010), untrained female | 30 | 45 | Unk. | Chamber | 15.0±0.4, 21.5±0.6, and 27.9±0.8 | 65 | 52±2.8 | 1.62±0.013 | 21±0.2 | Female | 42.9±1.2 | Unk. | 10 |
| 2: Ichinose-Kuwahara et al. (2010), trained female | 30 | 45 | Unk. | Chamber | 18.9±0.7, 27±1.0, and 35.0±1.2 | 65 | 54.2±2.2 | 1.602±0.018 | 20.4±0.3 | Female | 53.9±1.9 | Unk. | 10 |
| 3: Ichinose-Kuwahara et al. (2010), untrained male | 30 | 45 | Unk. | Chamber | 18.9±0.7, 27±1.0, and 35.0±1.2 | 65 | 64.3±3.3 | 1.742±0.017 | 21.1±0.4 | Male | 47.2±1.4 | Unk. | 9 |
| 4: Ichinose-Kuwahara et al. (2010), trained male | 30 | 45 | Unk. | Chamber | 18.9±0.7, 27±1.0, and 35.0±1.2 | 65 | 64.3±2.1 | 1.712±0.012 | 20.3±0.5 | Male | 56.6±2.2 | Unk. | 8 |
| 5: Muhamed et al. (2016), RH=23% | 31 | 23 | Unk. | Chamber | 42.7±4.2 | 60 | 72±6 | 1.80±0.06 | 30±4 | Male | 61±6 | False | 11 |
| 6: Muhamed et al. (2016), RH=43% | 31 | 43 | Unk. | Chamber | 42.7±4.2 | 60 | 72±6 | 1.80±0.06 | 30±4 | Male | 61±6 | False | 11 |
| 7: Muhamed et al. (2016), RH=52% | 31 | 52 | Unk. | Chamber | 42.7±4.2 | 60 | 72±6 | 1.80±0.06 | 30±4 | Male | 61±6 | False | 11 |
| 8: Muhamed et al. (2016), RH=61% | 31 | 61 | Unk. | Chamber | 42.7±4.2 | 60 | 72±6 | 1.80±0.06 | 30±4 | Male | 61±6 | False | 11 |
| 9: Muhamed et al. (2016), RH=71% | 31 | 71 | Unk. | Chamber | 42.7±4.2 | 60 | 72±6 | 1.80±0.06 | 30±4 | Male | 61±6 | False | 11 |
| 10: Lei et al. (2021), winter | 32 | 75 | 0-40min: 0.2,  40-60min: 1.1 | Chamber | 17.8±3.1 | 60 | 64.5±7.3 | 1.727±0.069 | 23±3 | Male | 44.4±7.8 | False | 12 |
| 11: Lei et al. (2021), summer | 32 | 75 | 0-40min: 0.2, 40-60min: 1.1 | Chamber | 16.0±1.8 | 60 | 64.2±7.4 | 1.726±0.069 | 24±3 | Male | 39.9±4.6 | True | 12 |

(Abbreviations – D: duration, H: height, HA: heat acclimatization, M: metabolic rate, RH: relative humidity, T_a_: ambient temperature, v: wind speed, Unk.: unknown, VO_2max_: maximal oxygen uptake, W: weight)

1. Equipment used to measure conditions of the general exercise experiments used to validate the accuracy of the joint system thermoregulation model [JOS-3]

| Experiments | Equipment used to measure conditions | | |
| --- | --- | --- | --- |
|  | Environmental conditions | VO_2max_ | Core temperature |
| 1: Ichinose-Kuwahara et al. (2010), untrained female | T_a_ and RH were controlled in a chamber (model EZ-101-MCU13,Tabai Espec, Osaka, Japan). | Estimated by extrapolating the relationship between O_2_ uptake and HR to the estimated maximal HR | Rectal thermometer (model unknown; inserted 8 to 10 cm beyond the anal sphincter) |
| 2: Ichinose-Kuwahara et al. (2010), trained female | T_a_ and RH were controlled in a chamber (model EZ-101-MCU13,Tabai Espec, Osaka, Japan). | Estimated by extrapolating the relationship between O_2_ uptake and HR to the estimated maximal HR | Rectal thermometer (model unknown; inserted 8 to 10 cm beyond the anal sphincter) |
| 3: Ichinose-Kuwahara et al. (2010), untrained male | T_a_ and RH were controlled in a chamber (model EZ-101-MCU13,Tabai Espec, Osaka, Japan). | Estimated by extrapolating the relationship between O_2_ uptake and HR to the estimated maximal HR | Rectal thermometer (model unknown; inserted 8 to 10 cm beyond the anal sphincter) |
| 4: Ichinose-Kuwahara et al. (2010), trained male | T_a_ and RH were controlled in a chamber (model EZ-101-MCU13,Tabai Espec, Osaka, Japan). | Estimated by extrapolating the relationship between O_2_ uptake and HR to the estimated maximal HR | Rectal thermometer (model unknown; inserted 8 to 10 cm beyond the anal sphincter) |
| 5: Muhamed et al. (2016), RH=23% | T_a_ and RH were controlled in a chamber. | A treadmill (model unknown) | Rectal thermometer (YSI 400 series; Mallinckrodt Medical, St. Louis, MO; inserted 12 cm beyond the anal sphincter) |
| 6: Muhamed et al. (2016), RH=43% | T_a_ and RH were controlled in a chamber. | A treadmill (model unknown) | Rectal thermometer (YSI 400 series; Mallinckrodt Medical, St. Louis, MO; inserted 12 cm beyond the anal sphincter) |
| 7: Muhamed et al. (2016), RH=52% | T_a_ and RH were controlled in a chamber. | A treadmill (model unknown) | Rectal thermometer (YSI 400 series; Mallinckrodt Medical, St. Louis, MO; inserted 12 cm beyond the anal sphincter) |
| 8: Muhamed et al. (2016), RH=61% | T_a_ and RH were controlled in a chamber. | A treadmill (model unknown) | Rectal thermometer (YSI 400 series; Mallinckrodt Medical, St. Louis, MO; inserted 12 cm beyond the anal sphincter) |
| 9: Muhamed et al. (2016), RH=71% | T_a_ and RH were controlled in a chamber. | A treadmill (model unknown) | Rectal thermometer (YSI 400 series; Mallinckrodt Medical, St. Louis, MO; inserted 12 cm beyond the anal sphincter) |
| 10: Lei et al. (2021), winter | T_a_ and RH were controlled in a chamber (Model: FLC 2700s, Fuji Medical Science, Japan). | A cycle ergometer (Aerobike, 75XLIII, Konami, Japan) | Rectal thermometer (model unknown; inserted 12 cm beyond the anal sphincter) |
| 11: Lei et al. (2021), summer | T_a_ and RH were controlled in a chamber (Model: FLC 2700s, Fuji Medical Science, Japan). | A cycle ergometer (Aerobike, 75XLIII, Konami, Japan) | Rectal thermometer (model unknown; inserted 12 cm beyond the anal sphincter) |

(Abbreviations – HR: heart rate, RH: relative humidity, Ta: ambient temperature, VO2max: maximal oxygen uptake)

1. Conditions of the sports experiments to validate the accuracy of the joint system thermoregulation model [JOS-3]; Values represent means, with standard deviations appended where available.

| Experiments | Environment | | | | Exercise | | Individual | | | | | | n |
| --- | --- | --- | --- | --- | --- | --- | --- | --- | --- | --- | --- | --- | --- |
|  | T_a_ (℃) | RH (%) | v (m/s) | Location | M (ml・kg^-1^・min^-1^) | D (min) | W (kg) | H (m) | Age (year) | Sex | VO_2max_ (ml・kg^-1^・min^-1^) | HA |  |
| 1 marathon, Noakes et al. (1991) | 20.5 | 68 | 0.3 to 3 | 1987 42.2km Cape Peninsula marathon | 0-42km: 44.1±6.0 36-42km: 38.2±5.9 | 207±26 | 75.7±10.9 | 1.78±0.07 | 36.7±7.5 | Male | 58.3±5.9 | Unk. | 30 |
| 2: marathon, Coso et al. (2013) | 27 | 27 | Unk. | 2012 Madrid Marathon | Unk. | 192±33 | 70±9 | 1.72±0.07 | 41±8 | Male | Unk. | Unk. | 40 |
| 3: marathon, Racinais et al. (2021), female | 32 | 77.9 | 0.1±0.2 | Doha 2019 | Unk. | 169±10 | 48.3±4.3 | 1.62±0.05 | 32.6±6.0 | Female | Unk. | Unk. | 15 |
| 4: marathon, Racinais et al. (2021), male | 29.3 | 46.3 | 0.4±0.5 | Doha 2019 | Unk. | 141±7 | 60.8±4.5 | 1.76±0.07 | 30.6±3.3 | Male | Unk. | Unk. | 14 |
| 5: football, Edwards and Clark (2006), recreational players | 16 | 47 | Unk. | A soccer field | Unk. | 1st half: 45 HT: 15 2nd half: 45 | 81.1±3.9 | 1.79±0.02 | 20.0±2.2 | Male | 52.73±4.1 | Unk. | 8 |
| 6: football, Edwards and Clark (2006), professional players | 19 | 53 | Unk. | A soccer field | Unk. | 1st half: 45 HT: 15 2nd half: 45 | 79.5±2.6 | 1.79±0.04 | 24±3 | Male | 65.62±4.9 | Unk. | 7 |
| 7: football, Özgünen et al. (2010), moderate heat | 34 | 38 | Unk. | A soccer field | Unk. | 1st half: 45 HT: 15 2nd half: 45 | 68.5±5.3 | 1.77±0.05 | 20.4±2.1 | Male | 62.6±6.8 | False | 11 |
| 8: football, Özgünen et al. (2010), high heat | 36 | 61 | Unk. | A soccer field | Unk. | 1st half: 45 HT: 15 2nd half: 45 | 68.5±5.3 | 1.77±0.05 | 20.4±2.1 | Male | 62.6±6.8 | False | 11 |
| 9: football, Chalmers et al. (2019), high heat | 35.7 | 52.5 | 1 | Chamber | Unk. | 1st half: 45 HT: 20 2nd half: 45 | 75±6 | 1.79±0.05 | 24±7 | Male | 58±3 | Unk. | 12 |
| 10: rowing, Taylor et al. (2014), cool | 20 | 40 | Unk. | Chamber | Unk. | 9 | 66.8±3.1 | 1.66±0.04 | 19.9±1.5 | Female | Unk. | False | 8 |
| 11: rowing, Taylor et al. (2014), hot | 35 | 60 | Unk. | Chamber | Unk. | 9 | 66.8±3.1 | 1.66±0.04 | 19.9±1.5 | Female | Unk. | False | 8 |
| 12: rugby sevens, Fenemor et al. (2021), day 1 | Game1: 31.1 Game2: 29.0 | Game1: 71 Game2: 73 | Unk. | Oceania sevens tournament | Unk. | 243 | 94.3±7.5 | 1.87±0.05 | 24±3 | Male | Unk. | False | 11 |
| 13: rugby sevens, Fenemor et al. (2021), day 2 | Game 3: 30.4 Game 4: 29.9 Game 5: 26.0 | Game 3: 73 Game 4: 75 Game 5: 81 | Unk. | Oceania sevens tournament | Unk. | 534 | 94.3±7.5 | 1.87±0.05 | 24±3 | Male | Unk. | False | 11 |
| 14: tennis, Schranner et al. (2017), no cooling | 36.5 | 51 | Unk. | Chamber | Unk. | 94 | 77.3±6.7 | 1.79±0.06 | 25±4 | Male | 50.6±6.3 | False | 9 |
| 15: tennis, Lynch et al. (2018), no cooling | 45 | 9 | 0.2 | Chamber | Exercise: 26.7±1.7 Recovery and break: Unk. | 113 | 73.9±5.0 | 1.79±0.05 | 22±3 | Male | 51.7±4.6 | Unk. | 9 |
| 16: tennis, Naito et al. (2018), water trial | 36.5 | 50 | Unk. | Chamber | Unk. | 81 | 64.8±6.8 | 1.72±0.08 | 22±2 | Male | Unk. | Unk. | 7 |
| 17: triathlon (only cycling and running), Chan et al. (2008), moderate | 22 | 76 | Unk. | Chamber | Unk. | Cycling: 68±3 Running: 51±4 | 63.7±2.3 | 1.65±0.02 | 26.1±1.6 | Male | Cycling: 62.2±2.2 Running: 67.8±1.5 | Unk. | 7 |
| 18: triathlon (only cycling and running), Chan et al. (2008), hot | 31.2 | 76.4 | Unk. | Chamber | Unk. | Cycling: 69±3 Running: 59±5 | 63.7±2.3 | 1.65±0.02 | 26.1±1.6 | Male | Cycling: 62.2±2.2 Running: 67.8±1.5 | Unk. | 7 |

(Abbreviations – D: duration, Doha 2019: the Doha 2019 IAAF World Athletics Championships, H: height, HA: heat acclimatization, HT: halftime, M: metabolic rate, RH: relative humidity, Ta: ambient temperature, v: wind speed, Unk.: unknown, VO2max: maximal oxygen uptake, W: weight, Unk.: unknown)

1. Equipment used to measure conditions of the sports experiments used to validate the accuracy of the joint system thermoregulation model [JOS-3]

| Experiments | Equipment used to measure conditions | | |
| --- | --- | --- | --- |
|  | Environmental conditions | VO_2max_ | Core temperature |
| 1: marathon, Noakes et al. (1991) | Obtained from the local meteorological office | A treadmill (model unknown) | Rectal thermometer (inserted 5 cm into the rectum and left in place for a minimum of 3 min) (measured within 2-5 min of the finish) |
| 2: marathon, Coso et al. (2013) | Unknown | N/A | Ingestible telemetry pills (HT150002, HQ Inc, US; ingested at least three hours before the race) |
| 3: marathon, Racinais et al. (2021), female | A heat stress monitor (Kestrel 4400, Boothwyn, USA) was placed on a tripod approximately 1.5 m above the floor and 4 m from the course. | N/A | Ingestible telemetry pills (BodyCap, Caen, France; ingested 4 to 6 hours pre-race) |
| 4: marathon, Racinais et al. (2021), male | A heat stress monitor (Kestrel 4400, Boothwyn, USA) was placed on a tripod approximately 1.5 m above the floor and 4 m from the course. | N/A | Ingestible telemetry pills (BodyCap, Caen, France; ingested 4 to 6 hours pre-race) |
| 5: football, Edwards and Clark (2006), recreational players | Unknown | A treadmill (Woodway PPS 55, Weil am Rhein, Germany) | Ingestible telemetry pills (CorTemp, Human Technologies International, USA; ingested approximately 4 hours before matches) |
| 6: football, Edwards and Clark (2006), professional players | Unknown | A treadmill (Woodway PPS 55, Weil am Rhein, Germany) | Ingestible telemetry pills (CorTemp, Human Technologies International, USA; ingested approximately 4 hours before matches) |
| 7: football, Özgünen et al. (2010), moderate heat | T_a_ and RH were recorded at the side of the playing field, every 10 min during each game, by a meteorologist from the Turkish Regional Meteorology Department. | A treadmill (T-15, Cosmed, Italy) | Ingestible telemetry pills (VitalSense, Mini Mitter Co. Inc., USA); ingested approximately 4 hours before matches) |
| 8: football, Özgünen et al. (2010), high heat | T_a_ and RH were recorded at the side of the playing field, every 10 min during each game, by a meteorologist from the Turkish Regional Meteorology Department. | A treadmill (T-15, Cosmed, Italy) | Ingestible telemetry pills (VitalSense, Mini Mitter Co. Inc., USA); ingested approximately 4 hours before matches) |
| 9: football, Chalmers et al. (2019), high heat | A heat stress monitor (Kestrel 4600, Boothwyn, USA) was placed next to the treadmill. | A treadmill (model unknown) | Rectal thermometer (Covidien^TM^ Mon-a-Therm^TM^ 400TM, USA; inserted 15 cm past the anal sphincter) |
| 10: rowing, Taylor et al. (2014), cool | Ta and RH were controlled in a chamber (Custom made; T. I. S. S, Hampshire, United Kingdom). | N/A | Rectal thermometer (400H and 4491H, Henleys Medical Supplies, UK; inserted 10 cm past the anal sphincter) |
| 11: rowing, Taylor et al. (2014), hot | Ta and RH were controlled in a chamber (Custom made; T. I. S. S, Hampshire, United Kingdom). | N/A | Rectal thermometer (400H and 4491H, Henleys Medical Supplies, UK; inserted 10 cm past the anal sphincter) |
| 12: rugby sevens, Fenemor et al. (2021), day 1 | A heat stress monitor (Kestrel 4200, Boothwyn, USA) was placed at the end of the playing field. | N/A | Ingestible telemetry pills (BodyCap, Caen, France; ingested at least 5 hours before the first game) |
| 13: rugby sevens, Fenemor et al. (2021), day 2 | A heat stress monitor (Kestrel 4200, Boothwyn, USA) was placed at the end of the playing field. | N/A | Ingestible telemetry pills (BodyCap, Caen, France; ingested at least 5 hours before the first game) |
| 14: tennis, Schranner et al. (2017), no cooling | T_a_ and RH were controlled in a chamber. | A treadmill (model unknown) was used. | Rectal thermometer (Covidien^TM^ Mon-a-Therm^TM^ 400TM, USA; inserted 15 cm past the anal sphincter) |
| 15: tennis, Lynch et al. (2018), no cooling | T_a_ and RH were controlled in a chamber. | A treadmill (h/p cosmos Saturn 300/125, Germany) was used. | Rectal thermometer (Covidien^TM^ Mon-a-Therm^TM^ 400TM, USA; inserted 15 cm past the anal sphincter) |
| 16: tennis, Naito et al. (2018), water trial | T_a_ and RH were controlled in a chamber. | A treadmill (BM-1200 Biomill, S & ME, Tokyo, Japan) was used. | Rectal thermometer (ITP010-11; Nikkiso-Therm CO., Ltd., Tokyo, Japan; inserted approximately 15 cm into the rectum) |
| 17: triathlon (only cycling and running), Chan et al. (2008), moderate | T_a_ and RH were controlled in a chamber. | A cycling ergometer (818E, Monark, Stockholm, Sweden) and a treadmill (LE 500C, Jaegar, Traustein, Germany) were used. | Rectal thermometer (Edale Instruments Ltd., Cambridge, UK; inserted 10 cm beyond the anal sphincter) |
| 18: triathlon (only cycling and running), Chan et al. (2008), hot | T_a_ and RH were controlled in a chamber. | A cycling ergometer (818E, Monark, Stockholm, Sweden) and a treadmill (LE 500C, Jaegar, Traustein, Germany) were used. | Rectal thermometer (Edale Instruments Ltd., Cambridge, UK; inserted 10 cm beyond the anal sphincter) |

(Abbreviations –RH: relative humidity, T_a_: ambient temperature, VO_2max_: maximal oxygen uptake)

1. Main parameter settings for six target sports in the joint system thermoregulation model [JOS-3]

| Sports  (guidelines’ upper thresholds) | T_a_ (℃) | RH (%) | T_r_ (℃) | v (m/s) | M (ml・　kg^-1^・min^-1^) | D (min) | W (kg) | H (m) | Age (year) | Sex |
| --- | --- | --- | --- | --- | --- | --- | --- | --- | --- | --- |
| Football  (WBGT  32℃) | 39  (HT:15) | 20  (HT:50) | 59  (HT:15) | 1  (HT: 5) | Game: 35.0 (SD:3.5)  HT: 8.8  (SD:0.9) | 105 (1^st^ half:45, HT:15, 2^nd^ half:45) | 75  (SD:10) | 1.75  (SD:  0.10) | 35  (SD:10) | Male |
|  | 32.5  (HT:15) | 50  (HT:50) | 52.5  (HT:15) |  |  |  |  |  |  |  |
|  | 28  (HT:15) | 80  (HT:50) | 48  (HT:15) |  |  |  |  |  |  |  |
| Marathon  (WBGT  28℃) | 35 | 20 | 55 | 1 | 0-36km: 44.0 (SD:4.4)  36-42.195km: 38.0 (SD:3.8) | 210 (SD:30) |  |  |  |  |
|  | 29 | 50 | 49 |  |  |  |  |  |  |  |
|  | 25 | 80 | 45 |  |  |  |  |  |  |  |
| Rowing  (Ta38℃/WBGT  32℃) | 38 | 20 | 38 | 1 | 43.8 (SD:4.4) | 9 |  |  |  |  |
|  | 32.5 | 50 | 32.5 |  |  |  |  |  |  |  |
|  | 28 | 80 | 28 |  |  |  |  |  |  |  |
| Seventh Rugby  (HSI150) | 40  (break:  15) | 40  (break:  50) | 60  (break:  15) | 1 | WU: 29.0 (SD:2.9)  PREP: 8.8 (SD:0.9)  Game: 39.0 (SD:3.9)  Break: 8.8 (SD:0.9) | 246 (WU:30, PREP:10, 1^st^ game: 16, break: 134, WU:30, PREP:10, 2^nd^ game:16) |  |  |  |  |
|  | 35  (break:  15) | 50  (break:  50) | 55  (break:  15) |  |  |  |  |  |  |  |
|  | 30  (break:  15) | 60  (break:  50) | 50  (break:  15) |  |  |  |  |  |  |  |
|  | 26  (break:  15) | 70  (break:  50) | 46  (break:  15) |  |  |  |  |  |  |  |
| Tennis  (WBGT  32.2℃) | 39 | 20 | 59  (break:  39) | 1 | Point:{16km/h run:57.0, 9km/h run:34.0, 2km/h run:7.0, Rest: 6.3},  Break: 5.3  (SD:10%) | 81 (Game:16s*6point*8games*4set, Break:90s*16+120s*3) |  |  |  |  |
|  | 32.5 | 50 | 52.5  (break:  32.5) |  |  |  |  |  |  |  |
|  | 28 | 80 | 48  (break:  28) |  |  |  |  |  |  |  |
| Triathlon  (WBGT  32.2℃) | 39 | 20 | 59 | 1 | Cycling: 45.5 (SD:4.6)  Running: 42.0 (SD:4.2) | 119 (SD:7) |  |  |  |  |
|  | 32.5 | 50 | 52.5 |  |  |  |  |  |  |  |
|  | 28 | 80 | 48 |  |  |  |  |  |  |  |

(Abbreviations – D: duration, H: height, HSI: Heat Stress Index, HT: halftime, PREP: preparation, RH: relative humidity, M: metabolic rate, SD: standard deviation, T_a_: ambient temperature, T_r_: mean radiative temperature, WBGT: Wet Bulb Globe Temperature, v: wind speed, W: weight, WU: warm-up)

1. Parameter settings of the joint system thermoregulation model [JOS-3] (case 1: common laboratory exercises, Ichinose-Kuwahara et al. (2010), untrained female, n=10)

| Parameter | Setting | Description |
| --- | --- | --- |
| Ambient temperature:  T_a_ (℃) | 30.0 | Same as the reference value |
| Relative humidity:  RH (%) | 45 | Same as the reference value |
| Mean radiative temperature: T_r_ (℃) | 30.0 | Measured values unknown; assumed to be same as T_a_ (Indoor) |
| Wind speed: v (m/s) | 0.2 | Measured values unknown; assumed as 0.2 |
| Metabolic rate:  M (ml・kg^-1^・min^-1^) | Rest: 3.5  Exercise: 42.9±0.8×0.35,  42.9±0.8×0.50, and  42.9±0.8×0.65  (35%, 50%, and 65% of VO_2max_,  VO_2max_: 42.9±1.2) | Avg of measured values ± SD |
| Duration: D (min) | Rest: 5  Exercise: 60 | Same as the reference value |
| Weight: W (kg) | 52±2.8 | Avg of measured values ± SD |
| Height: H (m) | 1.62±0.013 | Avg of measured values ± SD |
| Age (year) | 21±0.2 | Avg of measured values ± SD |
| Sex | Female | Same as the reference value |
| Clo | 0.1 | Measured values unknown; assumed as 0.1 |


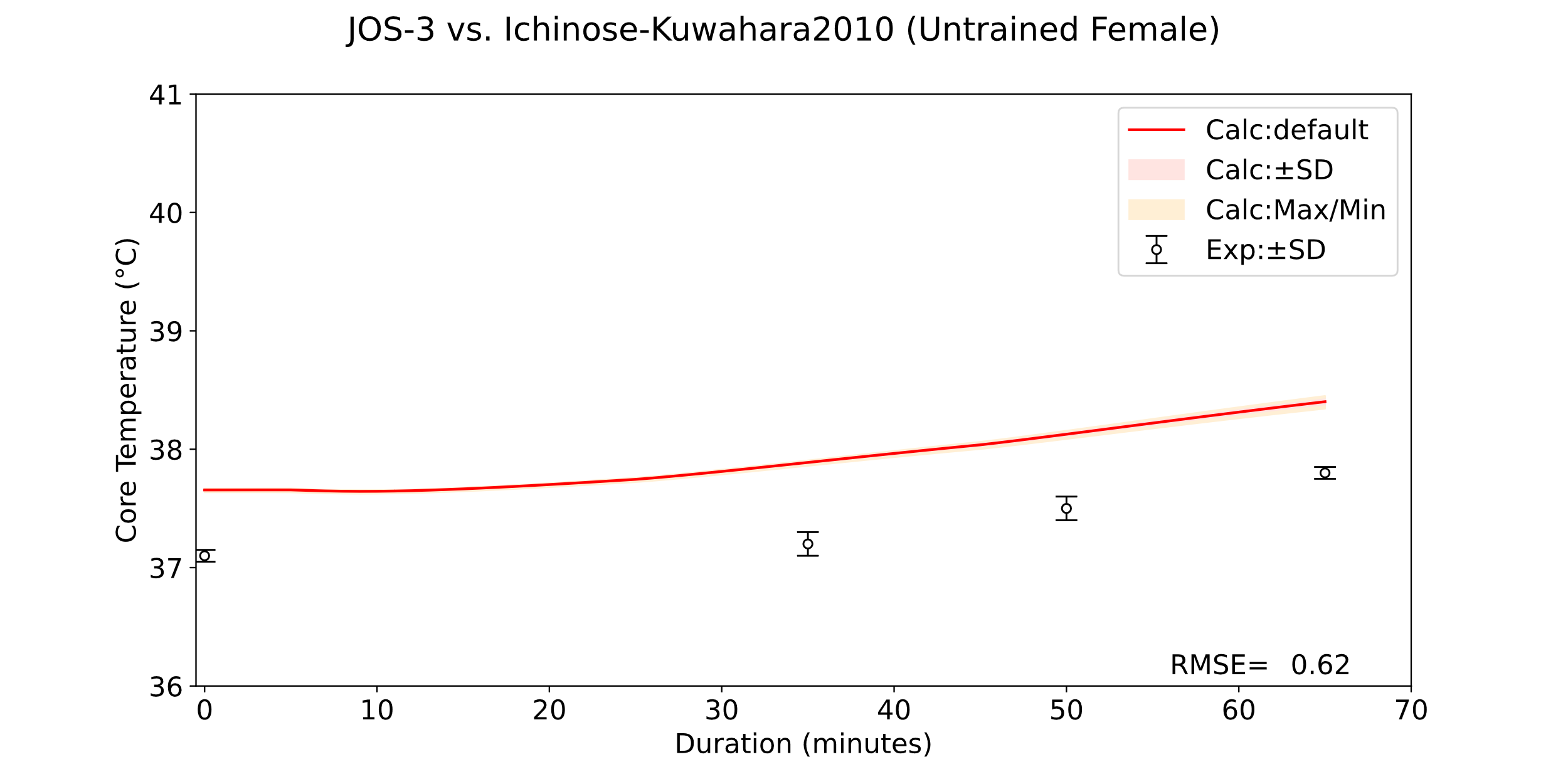


Supplementary Fig. 1 Core temperature reproduced by the joint system thermoregulation model [JOS-3] (case 1: common laboratory exercises, Ichinose-Kuwahara et al. (2010), untrained female, n=10); For the four parameters of height, weight, age, and metabolic rate, three patterns of mean values and mean ± standard deviation were set and exhaustively combined, resulting in 81 calculation patterns.

1. Parameter settings of the joint system thermoregulation model [JOS-3] (case 2: common laboratory exercises, Ichinose-Kuwahara et al. (2010), trained female, n=10)

| Parameter | Setting | Description |
| --- | --- | --- |
| Ambient temperature:  Ta (℃) | 30.0 | Same as the reference value |
| Relative humidity:  RH (%) | 45 | Same as the reference value |
| Mean radiative temperature: Tr (℃) | 30.0 | Measured values unknown; assumed to be same as Ta (Indoor) |
| Wind speed: v (m/s) | 0.2 | Measured values unknown; assumed as 0.2 |
| Metabolic rate:  M (ml・kg^-1^・min^-1^) | Rest: 3.5  Exercise: 53.9±1.9×0.35, 53.9±1.9×0.50, and  53.9±1.9×0.65  (35%, 50%, and 65% of VO_2max_,  VO_2max_: 53.9±1.9) | Avg of measured values ± SD |
| Duration: D (min) | Rest: 5  Exercise: 60 | Same as the reference value |
| Weight: W (kg) | 54.2±2.2 | Avg of measured values ± SD |
| Height: H (m) | 1.602±0.018 | Avg of measured values ± SD |
| Age (year) | 20.4±0.3 | Avg of measured values ± SD |
| Sex | Female | Same as the reference value |
| Clo | 0.1 | Measured values unknown; assumed as 0.1 |


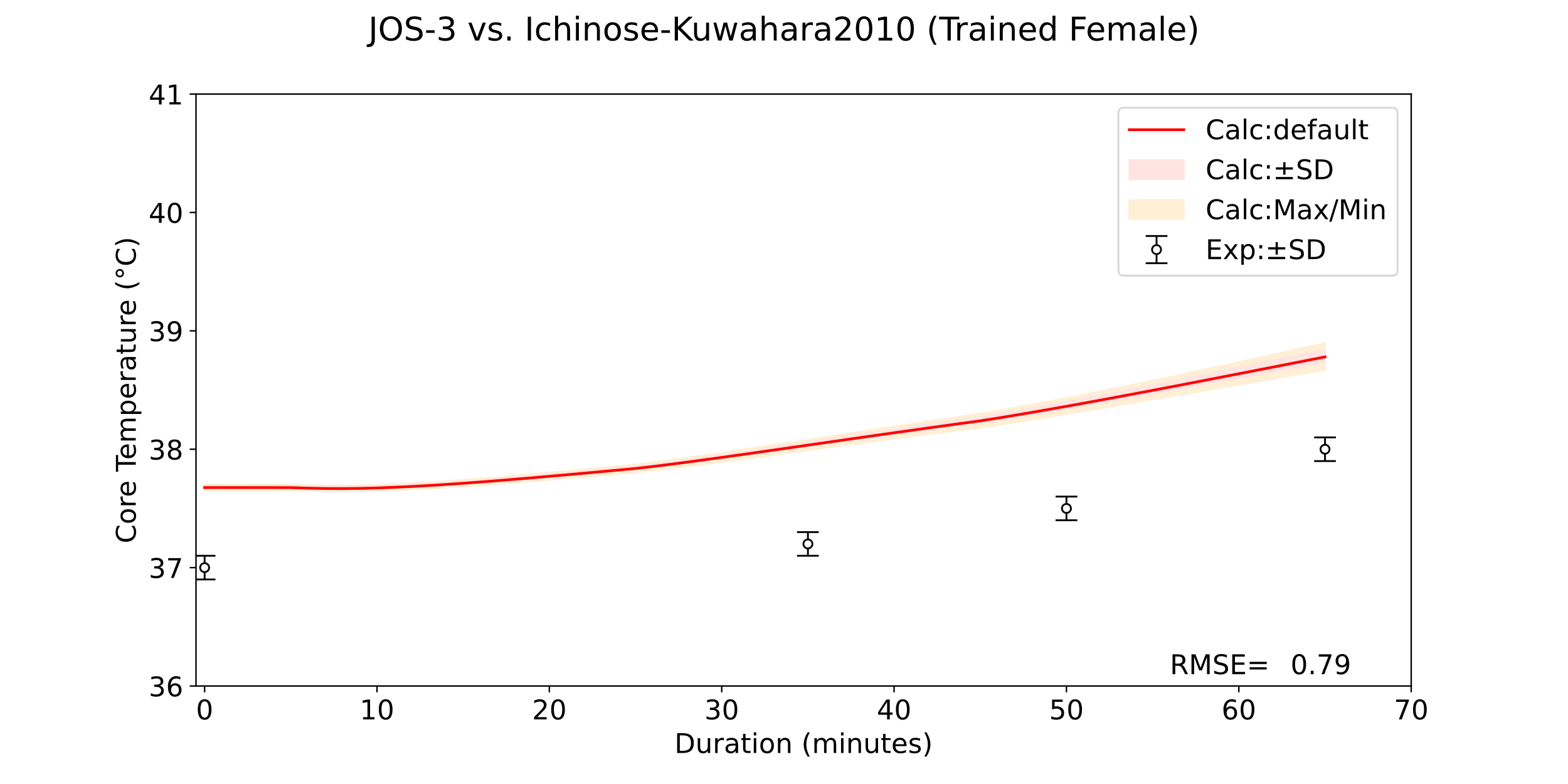


Supplementary Fig. 2 Core temperature reproduced by the joint system thermoregulation model [JOS-3] (case 2: common laboratory exercises, Ichinose-Kuwahara et al. (2010), trained female, n=10); For the four parameters of height, weight, age, and metabolic rate, three patterns of mean values and mean ± standard deviation were set and exhaustively combined, resulting in 81 calculation patterns.

1. Parameter settings of the joint system thermoregulation model [JOS-3] (case 3: common laboratory exercises, Ichinose-Kuwahara et al. (2010), untrained male, n=9)

| Parameter | Setting | Description |
| --- | --- | --- |
| Ambient temperature:  Ta (℃) | 30.0 | Same as the reference value |
| Relative humidity:  RH (%) | 45 | Same as the reference value |
| Mean radiative temperature: Tr (℃) | 30.0 | Measured values unknown; assumed to be same as Ta (Indoor) |
| Wind speed: v (m/s) | 0.2 | Measured values unknown; assumed as 0.2 |
| Metabolic rate:  M (ml・kg^-1^・min^-1^) | Rest: 3.5  Exercise: 47.2±1.4×0.35, 47.2±1.4×0.50, and  47.2±1.4×0.65  (35%, 50%, and 65% of VO_2max_,  VO_2max_: 47.2±1.4) | Avg of measured values ± SD |
| Duration: D (min) | Rest: 5  Exercise: 60 | Same as the reference value |
| Weight: W (kg) | 64.3±3.3 | Avg of measured values ± SD |
| Height: H (m) | 1.742±0.017 | Avg of measured values ± SD |
| Age (year) | 21.1±0.4 | Avg of measured values ± SD |
| Sex | Male | Same as the reference value |
| Clo | 0.1 | Measured values unknown; assumed as 0.1 |


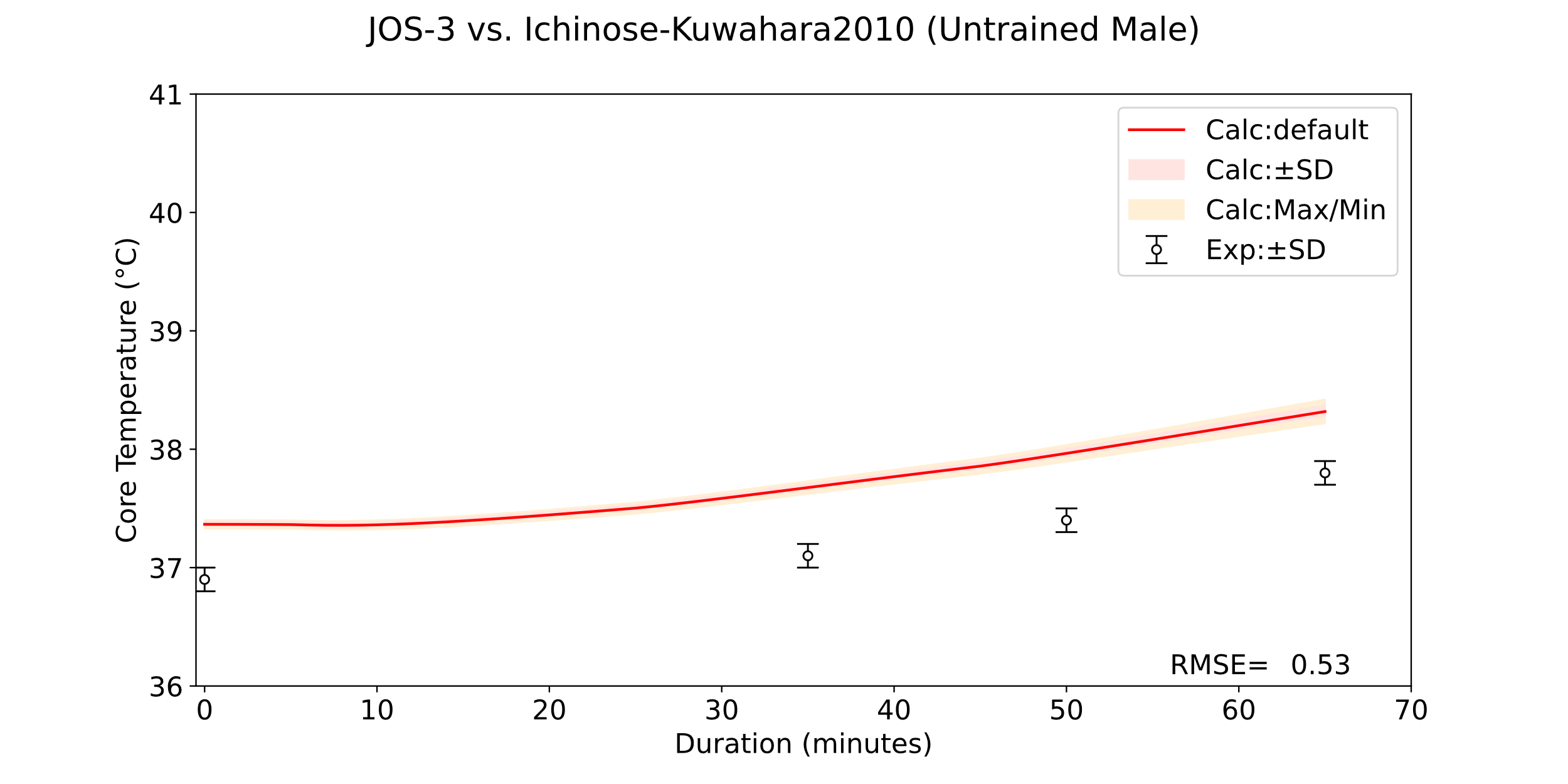


Supplementary Fig. 3 Core temperature reproduced by the joint system thermoregulation model [JOS-3] (case 3: common laboratory exercises, Ichinose-Kuwahara et al. (2010), untrained male, n=9); For the four parameters of height, weight, age, and metabolic rate, three patterns of mean values and mean ± standard deviation were set and exhaustively combined, resulting in 81 calculation patterns.

1. Parameter settings of the joint system thermoregulation model [JOS-3] (case 4: common laboratory exercises, Ichinose-Kuwahara et al. (2010), trained male, n=8)

| Parameter | Setting | Description |
| --- | --- | --- |
| Ambient temperature:  Ta (℃) | 30.0 | Same as the reference value |
| Relative humidity: RH (%) | 45 | Same as the reference value |
| Mean radiative temperature: Tr (℃) | 30.0 | Measured values unknown; assumed to be same as Ta (Indoor) |
| Wind speed: v (m/s) | 0.2 | Measured values unknown; assumed as 0.2 |
| Metabolic rate:  M (ml・kg^-1^・min^-1^) | Rest: 3.5  Exercise: 56.6±2.2×0.35, 56.6±2.2×0.50, and  56.6±2.2×0.65  (35%, 50%, and 65% of VO_2max_,  VO_2max_: 56.6±2.2) | Avg of measured values ± SD |
| Duration: D (min) | Rest: 5  Exercise: 60 | Same as the reference value |
| Weight: W (kg) | 64.3±2.1 | Avg of measured values ± SD |
| Height: H (m) | 1.712±0.012 | Avg of measured values ± SD |
| Age (year) | 20.3±0.5 | Avg of measured values ± SD |
| Sex | Male | Same as the reference value |
| Clo | 0.1 | Measured values unknown; assumed as 0.1 |


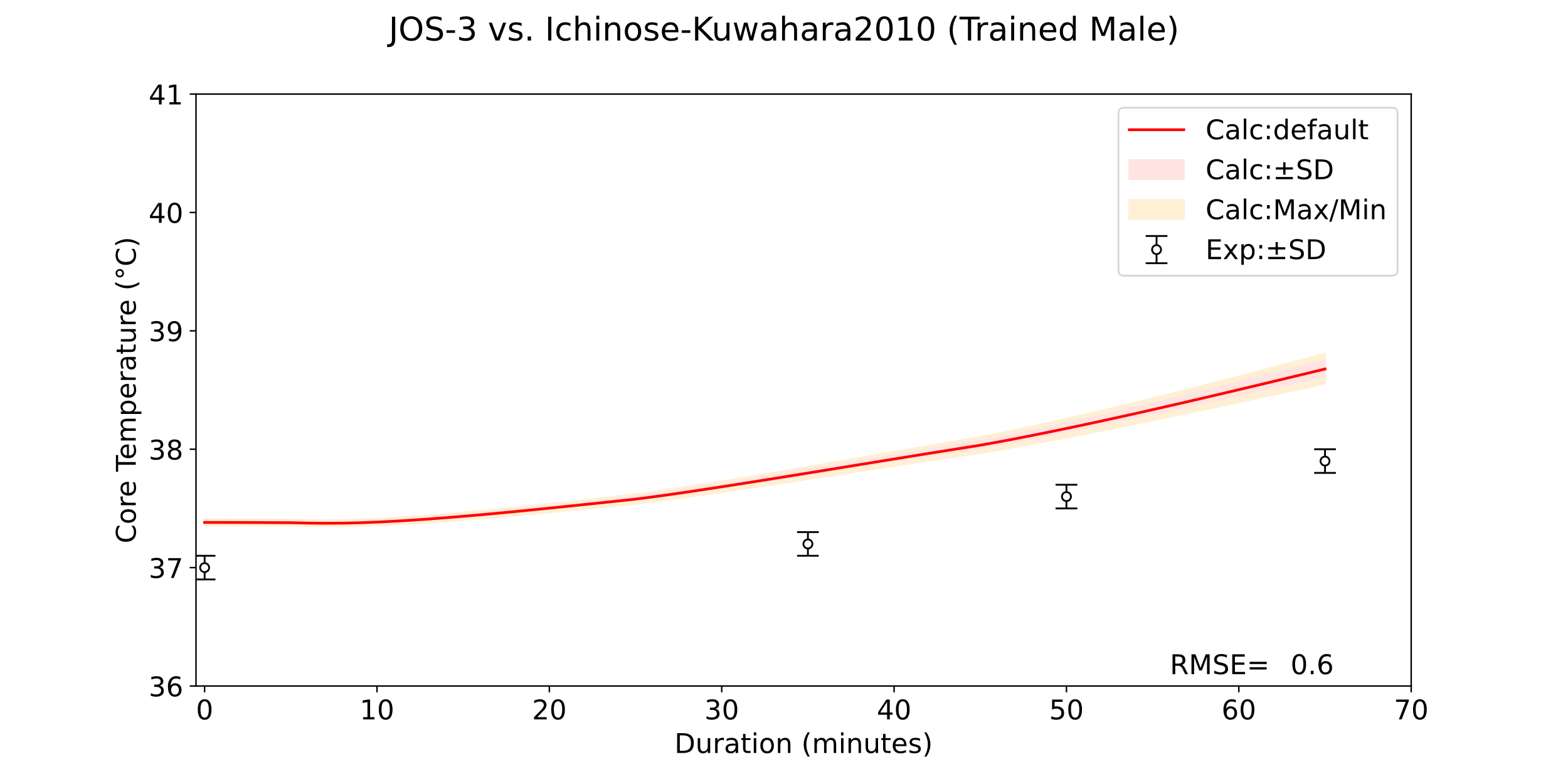


Supplementary Fig. 4 Core temperature reproduced by the joint system thermoregulation model [JOS-3] (case 4: common laboratory exercises, Ichinose-Kuwahara et al. (2010), trained male, n=8); For the four parameters of height, weight, age, and metabolic rate, three patterns of mean values and mean ± standard deviation were set and exhaustively combined, resulting in 81 calculation patterns.

1. Parameter settings of the joint system thermoregulation model [JOS-3] (case 5: common laboratory exercises, Muhamed et al. (2016), RH=23%, n=11)

| Parameter | Setting | Description |
| --- | --- | --- |
| Ambient temperature:  Ta (℃) | 31.0±0.2 | Avg of measured values ± SD |
| Relative humidity: RH (%) | 23 | Same as the reference value |
| Mean radiative temperature: Tr (℃) | 31.0±0.2 | Measured values unknown; assumed to be same as Ta (Indoor) |
| Wind speed: v (m/s) | 3.30±0.35 | Measured values unknown. Calculated from M using ACSM (2012) equation |
| Metabolic rate:  M (ml・kg^-1^・min^-1^) | 42.7±4.2  (VO_2max_: 61±6) | Avg of measured values ± SD |
| Duration: D (min) | 60 | Same as the reference value |
| Weight: W (kg) | 72±6 | Avg of measured values ± SD |
| Height: H (m) | 1.80±0.06 | Avg of measured values ± SD |
| Age (year) | 30±4 | Avg of measured values ± SD |
| Sex | Male | Same as the reference value |
| Clo | 0.2 | Measured values unknown; assumed as 0.2 |


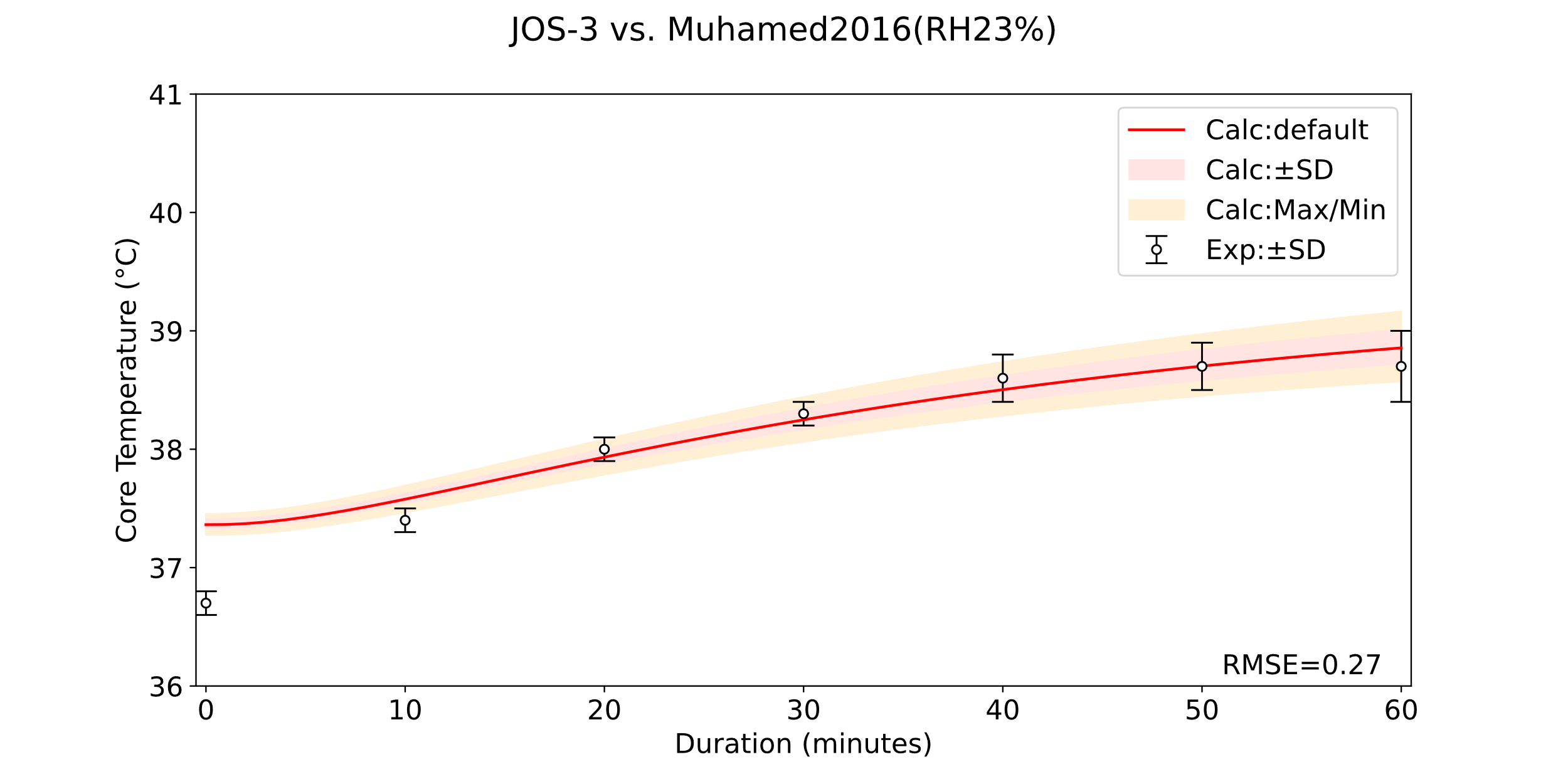


Supplementary Fig. 5 Core temperature reproduced by the joint system thermoregulation model [JOS-3] (case 5: common laboratory exercises, Muhamed et al. (2016), RH=23%, n=12); Three patterns of mean and mean ± standard deviation were set for the four parameters of height, weight, age, and metabolic rate, and three patterns for temperature trends, which were exhaustively combined, resulting in 243 calculation patterns.

1. Parameter settings of the joint system thermoregulation model [JOS-3] (case 6: common laboratory exercises, Muhamed et al. (2016), RH=43%, n=11)

| Parameter | Setting | Description |
| --- | --- | --- |
| Ambient temperature:  Ta (℃) | 31.0±0.2 | Avg of measured values ± SD |
| Relative humidity: RH (%) | 43 | Same as the reference value |
| Mean radiative temperature: Tr (℃) | 31.0±0.2 | Measured values unknown; assumed to be same as Ta (Indoor) |
| Wind speed: v (m/s) | 3.30±0.35 | Measured values unknown; Calculated from M using ACSM (2012) equation |
| Metabolic rate:  M (ml・kg^-1^・min^-1^) | 42.7±4.2  (VO_2max_: 61±6) | Avg of measured values ± SD |
| Duration: D (min) | 60 | Same as the reference value |
| Weight: W (kg) | 72±6 | Avg of measured values ± SD |
| Height: H (m) | 1.80±0.06 | Avg of measured values ± SD |
| Age (year) | 30±4 | Avg of measured values ± SD |
| Sex | Male | Same as the reference value |
| Clo | 0.2 | Measured values unknown; assumed as 0.2 |


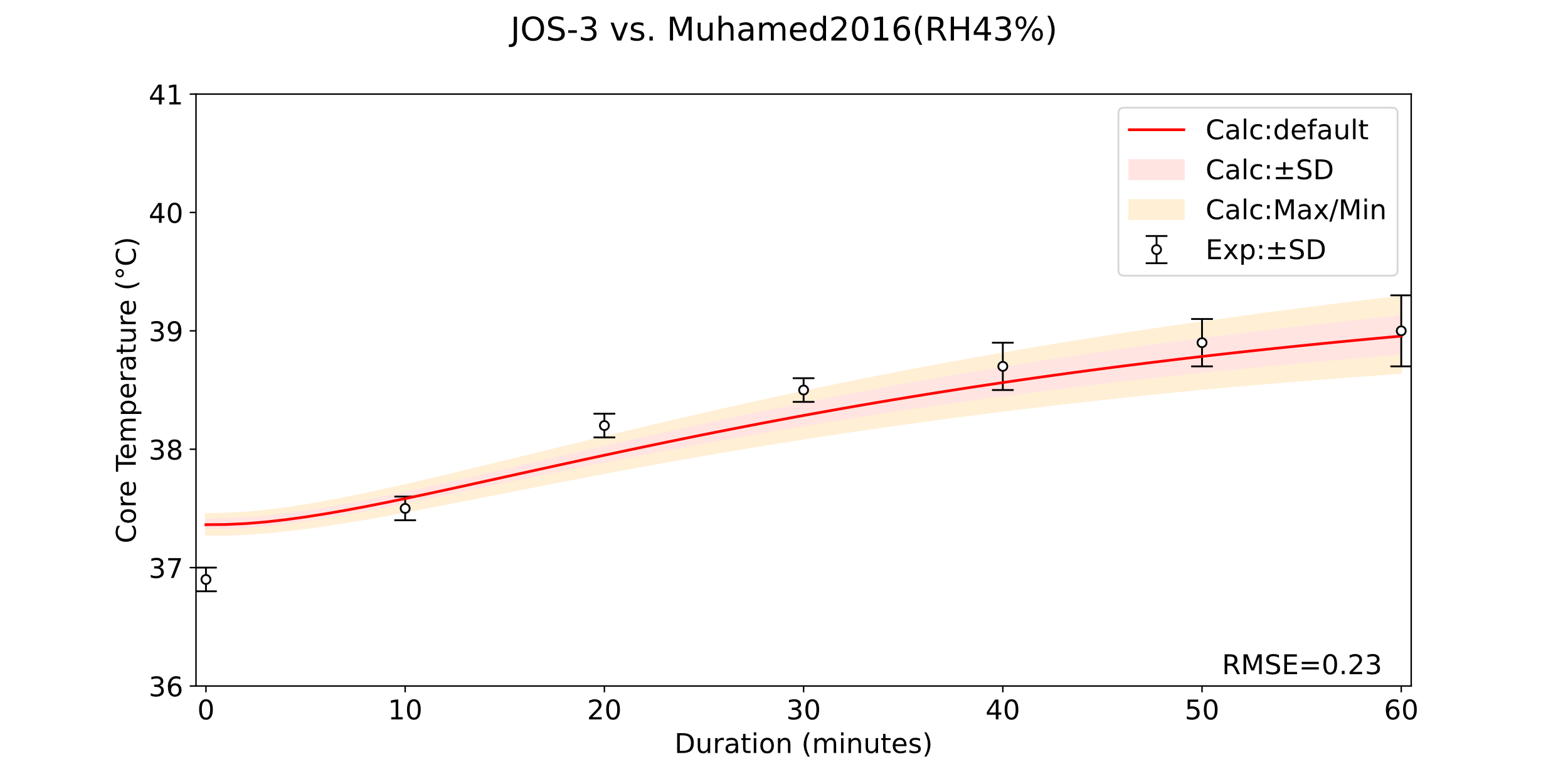


Supplementary Fig. 6 Core temperature reproduced by the joint system thermoregulation model [JOS-3] (case 6: common laboratory exercises, Muhamed et al. (2016), RH=43%, n=12); Three patterns of mean and mean ± standard deviation were set for the four parameters of height, weight, age, and metabolic rate, and three patterns for temperature trends, which were exhaustively combined, resulting in 243 calculation patterns.

1. Parameter settings of the joint system thermoregulation model [JOS-3] (case 7: common laboratory exercises, Muhamed et al. (2016), RH=52%, n=11)

| Parameter | Setting | Description |
| --- | --- | --- |
| Ambient temperature:  Ta (℃) | 31.0±0.2 | Avg of measured values ± SD |
| Relative humidity: RH (%) | 52 | Same as the reference value |
| Mean radiative temperature: Tr (℃) | 31.0±0.2 | Measured values unknown; assumed to be same as Ta (Indoor) |
| Wind speed: v (m/s) | 3.30±0.35 | Measured values unknown; calculated from M using ACSM (2012) equation |
| Metabolic rate: M (ml・kg^-1^・min^-1^) | 42.7±4.2  (VO_2max_: 61±6) | Avg of measured values ± SD |
| Duration: D (min) | 60 | Same as the reference value |
| Weight: W (kg) | 72±6 | Avg of measured values ± SD |
| Height: H (m) | 1.80±0.06 | Avg of measured values ± SD |
| Age (year) | 30±4 | Avg of measured values ± SD |
| Sex | Male | Same as the reference value |
| Clo | 0.2 | Measured values unknown; assumed as 0.2 |


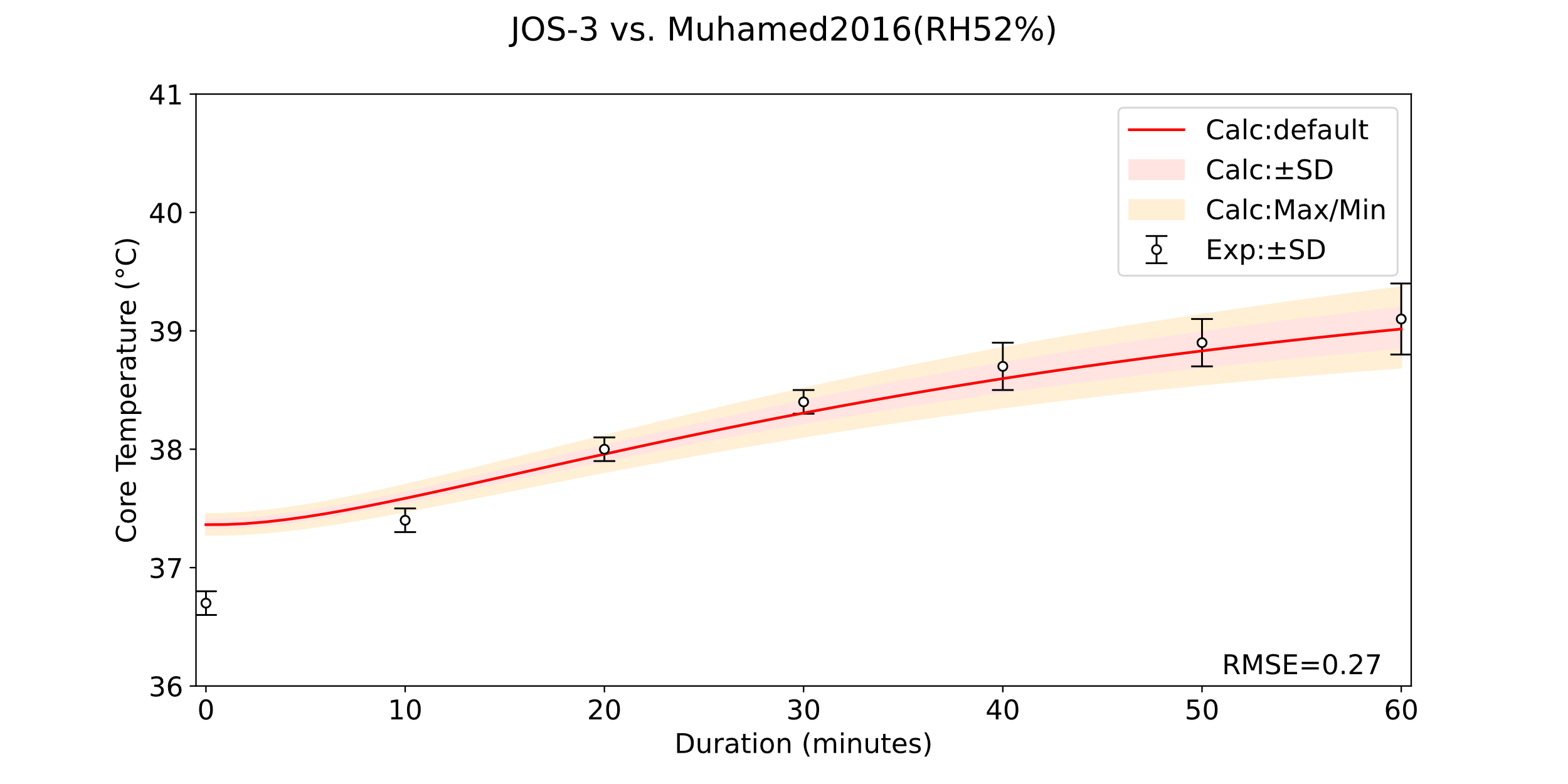


Supplementary Fig. 7 Core temperature reproduced by the joint system thermoregulation model [JOS-3] (case 7: common laboratory exercises, Muhamed et al. (2016), RH=52%, n=12); Three patterns of mean and mean ± standard deviation were set for the four parameters of height, weight, age, and metabolic rate, and three patterns for temperature trends, which were exhaustively combined, resulting in 243 calculation patterns.

1. Parameter settings of the joint system thermoregulation model [JOS-3] (case 8: common laboratory exercises, Muhamed et al. (2016), RH=61%, n=11)

| Parameter | Setting | Description |
| --- | --- | --- |
| Ambient temperature:  Ta (℃) | 31.0±0.2 | Avg of measured values ± SD |
| Relative humidity: RH (%) | 61 | Same as the reference value |
| Mean radiative temperature: Tr (℃) | 31.0±0.2 | Measured values unknown; assumed to be same as Ta (Indoor) |
| Wind speed: v (m/s) | 3.30±0.35 | Measured values unknown; calculated from M using ACSM (2012) equation |
| Metabolic rate:  M (ml・kg^-1^・min^-1^) | 42.7±4.2  (VO_2max_: 61±6) | Avg of measured values ± SD |
| Duration: D (min) | 60 | Same as the reference value |
| Weight: W (kg) | 72±6 | Avg of measured values ± SD |
| Height: H (m) | 1.80±0.06 | Avg of measured values ± SD |
| Age (year) | 30±4 | Avg of measured values ± SD |
| Sex | Male | Same as the reference value |
| Clo | 0.2 | Measured values unknown; assumed as 0.2 |


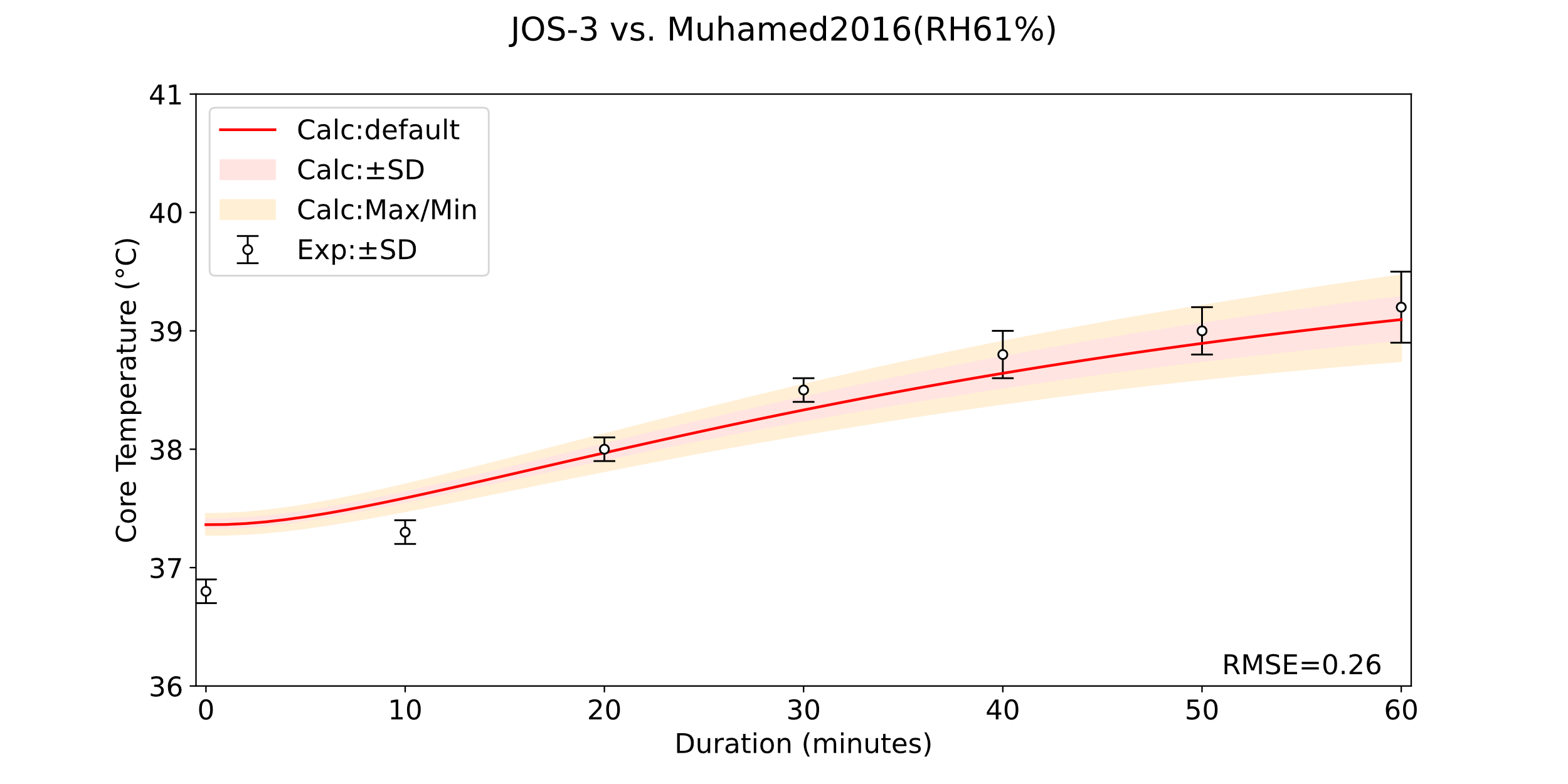


Supplementary Fig. 8 Core temperature reproduced by the joint system thermoregulation model [JOS-3] (case 8: common laboratory exercises, Muhamed et al. (2016), RH=61%, n=12); Three patterns of mean and mean ± standard deviation were set for the four parameters of height, weight, age, and metabolic rate, and three patterns for temperature trends, which were exhaustively combined, resulting in 243 calculation patterns.

1. Parameter settings of the joint system thermoregulation model [JOS-3] (case 9: common laboratory exercises, Muhamed et al. (2016), RH=71%, n=11)

| Parameter | Setting | Description |
| --- | --- | --- |
| Ambient temperature:  Ta (℃) | 31.0±0.2 | Avg of measured values ± SD |
| Relative humidity: RH (%) | 71 | Same as the reference value |
| Mean radiative temperature: Tr (℃) | 31.0±0.2 | Measured values unknown; assumed to be same as Ta (Indoor) |
| Wind speed: v (m/s) | 3.30±0.35 | Measured values unknown; calculated from M using ACSM (2012) equation |
| Metabolic rate:  M (ml・kg^-1^・min^-1^) | 42.7±4.2  (VO_2max_: 61±6) | Avg of measured values ± SD |
| Duration: D (min) | 60 | Same as the reference value |
| Weight: W (kg) | 72±6 | Avg of measured values ± SD |
| Height: H (m) | 1.80±0.06 | Avg of measured values ± SD |
| Age (year) | 30±4 | Avg of measured values ± SD |
| Sex | Male | Same as the reference value |
| Clo | 0.2 | Measured values unknown; assumed as 0.2 |


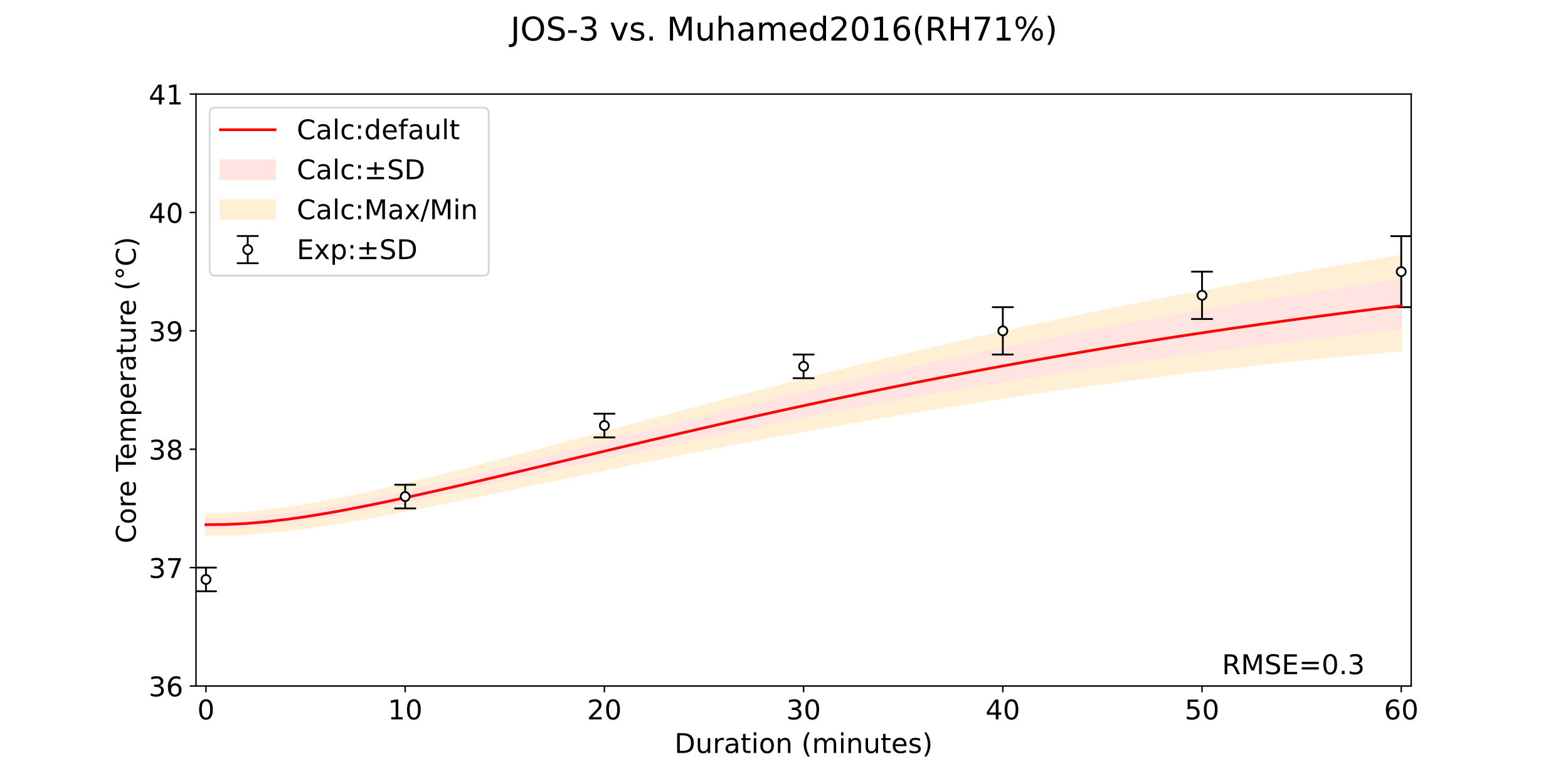


Supplementary Fig. 9 Core temperature reproduced by the joint system thermoregulation model [JOS-3] (case 9: common laboratory exercises, Muhamed et al. (2016), RH=71%, n=12); Three patterns of mean and mean ± standard deviation were set for the four parameters of height, weight, age, and metabolic rate, and three patterns for temperature trends, which were exhaustively combined, resulting in 243 calculation patterns.

1. Parameter settings of the joint system thermoregulation model [JOS-3] (case 10: common laboratory exercises, Lei et al. (2021), winter, n=12)

| Parameter | Setting | Description |
| --- | --- | --- |
| Ambient temperature: Ta (℃) | 32 | Same as the reference value |
| Relative humidity: RH (%) | 75 | Same as the reference value |
| Mean radiative temperature: Tr (℃) | 32 | Measured values unknown; assumed to be same as Ta (Indoor) |
| Wind speed: v (m/s) | 0-40min: 0.2  40-60min: 1.1 | Same as the reference value |
| Metabolic rate:  M (ml・kg^-1^・min^-1^) | 17.8±3.1  (VO_2max_: 44.4±7.8) | Avg of measured values ± SD |
| Duration: D (min) | 60 | Same as the reference value |
| Weight: W (kg) | 64.5±7.3 | Avg of measured values ± SD |
| Height: H (m) | 1.727±0.069 | Avg of measured values ± SD |
| Age (year) | 23±3 | Avg of measured values ± SD |
| Sex | Male | Same as the reference value |
| Clo | 0.2 | Measured values unknown; assumed as 0.2 |


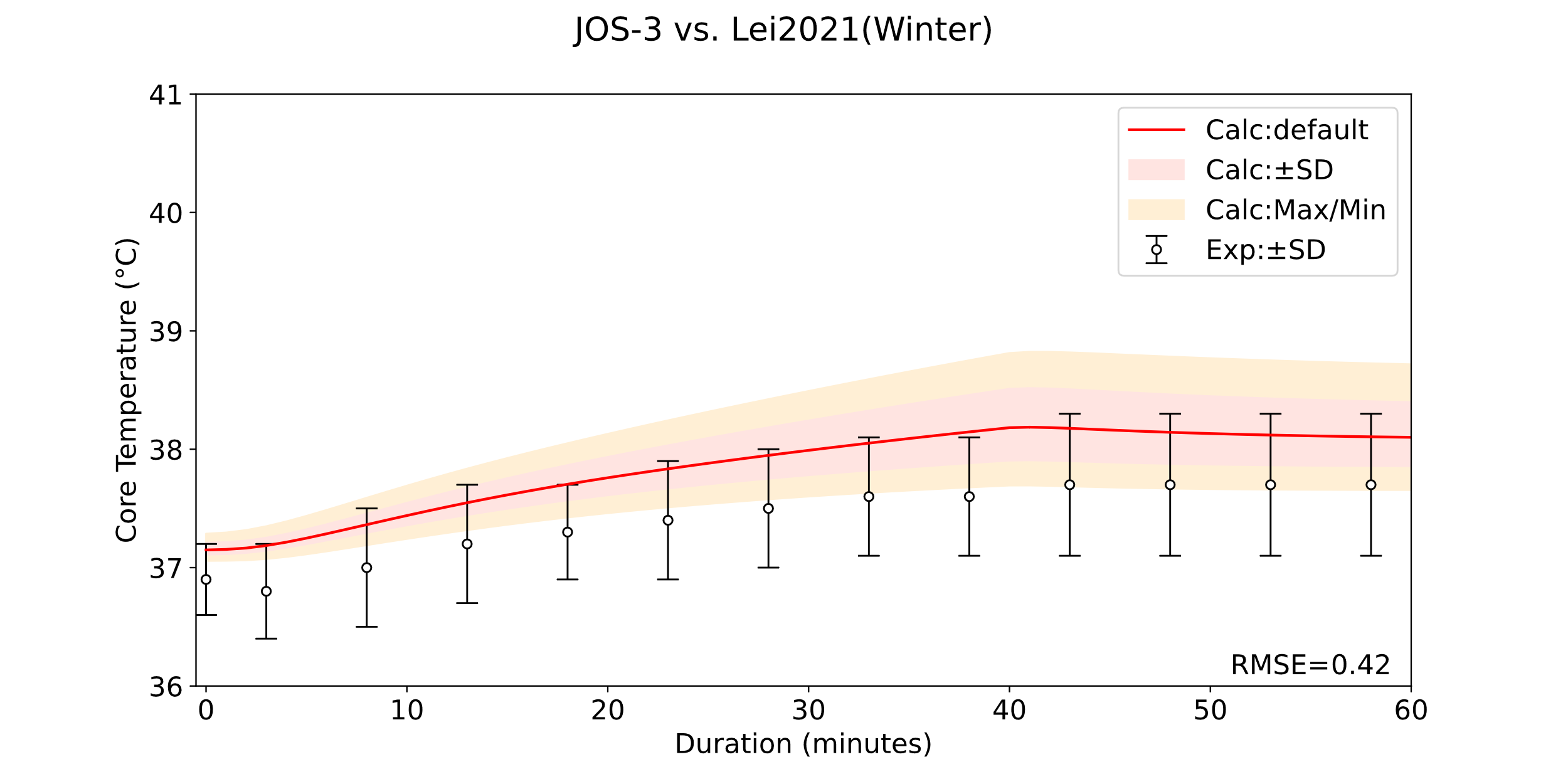


Supplementary Fig. 10 Core temperature reproduced by the joint system thermoregulation model [JOS-3] (case 10: common laboratory exercises, Lei et al. (2021), winter, n=12); For the four parameters of height, weight, age, and metabolic rate, three patterns of mean values and mean ± standard deviation were set and exhaustively combined, resulting in 81 calculation patterns.

1. Parameter settings of the joint system thermoregulation model [JOS-3] (case 11: common laboratory exercises, Lei et al. (2021), summer, n=12)

| Parameter | Setting | Description |
| --- | --- | --- |
| Ambient temperature: Ta (℃) | 32 | Same as the reference value |
| Relative humidity: RH (%) | 75 | Same as the reference value |
| Mean radiative temperature: Tr (℃) | 32 | Measured values unknown; assumed to be same as Ta (Indoor) |
| Wind speed: v (m/s) | 0-40min: 0.2  40-60min: 1.1 | Same as the reference value |
| Metabolic rate:  M (ml・kg^-1^・min^-1^) | 16.0±1.8  (VO_2max_: 39.9±4.6) | Avg of measured values ± SD |
| Duration: D (min) | 60 | Same as the reference value |
| Weight: W (kg) | 64.2±7.4 | Avg of measured values ± SD |
| Height: H (m) | 1.726±0.069 | Avg of measured values ± SD |
| Age (year) | 24±3 | Avg of measured values ± SD |
| Sex | Male | Same as the reference value |
| Clo | 0.2 | Measured values unknown; assumed as 0.2 |


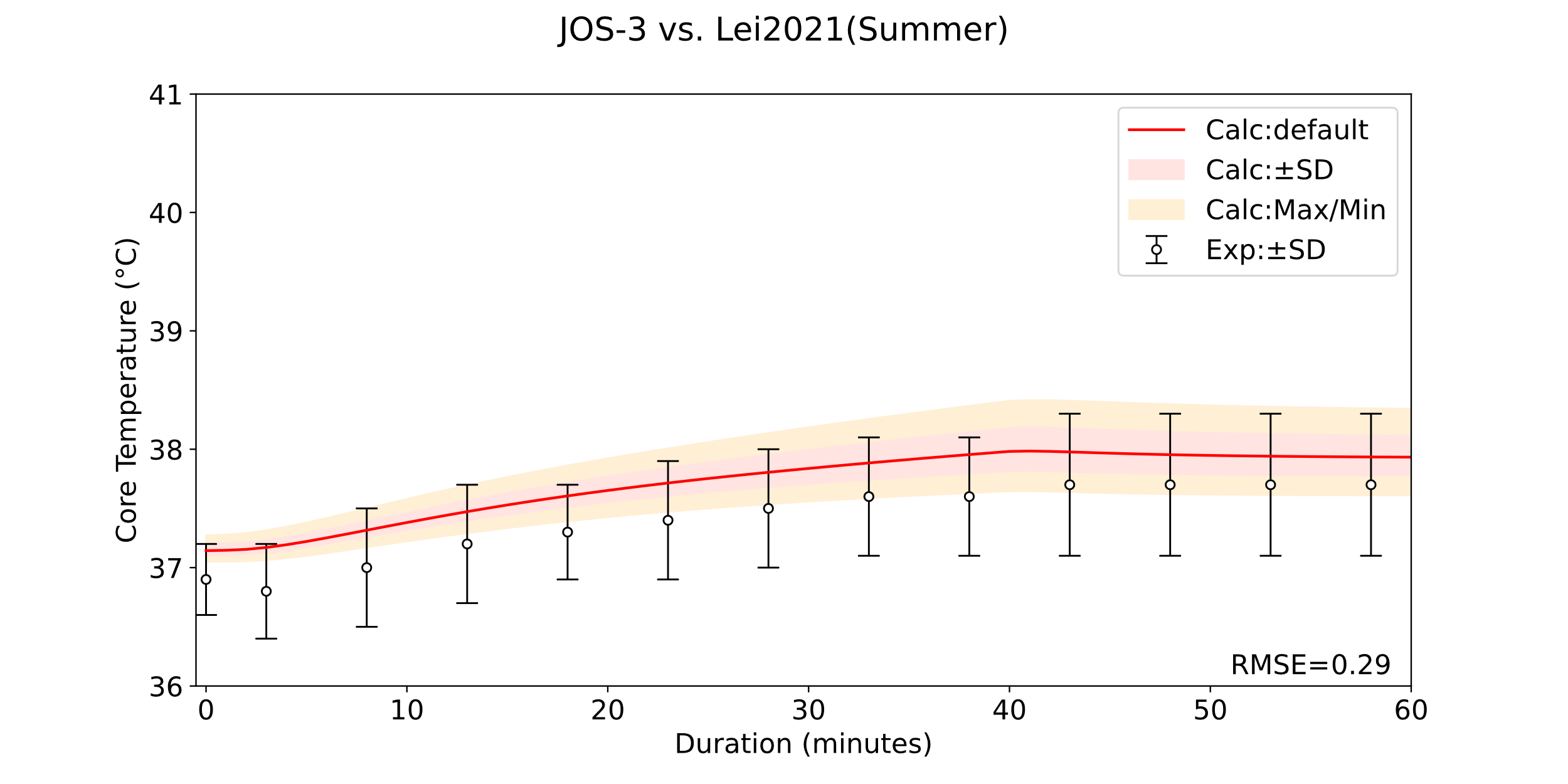


Supplementary Fig. 11 Core temperature reproduced by the joint system thermoregulation model [JOS-3] (case 11: common laboratory exercises, Lei et al. (2021), summer, n=12); For the four parameters of height, weight, age, and metabolic rate, three patterns of mean values and mean ± standard deviation were set and exhaustively combined, resulting in 81 calculation patterns.

1. Parameter settings of the joint system thermoregulation model [JOS-3] (case 12: marathon, Noakes et al. (1991), n=30)

| Parameter | Setting | Description |
| --- | --- | --- |
| Ambient temperature: Ta (℃) | [Pattern 1] 20.5  [Pattern 2] 1st half: 19, 2nd half: 22  [Pattern 3] 1st half: 22, 2nd half: 19 | [Pattern 1] Constant at the avg of measured values  [Pattern 2] Avg-SD in the 1st half, Avg+SD in the 2nd half  [Pattern 3] Avg+SD in the 1st half, Avg-SD in the 2nd half |
| Relative humidity:  RH (%) | 68.0 | Avg of measured values |
| Mean radiative temperature: Tr (℃) | [Pattern 1] 40.5  [Pattern 2] 1st half: 39, 2nd half: 42  [Pattern 3] 1st half: 42, 2nd half: 39 | Measured values unknown; assumed as Ta+20℃ (Daytime) |
| Wind speed: v (m/s) | 0-36km: 3.4±0.4+ 0 or 1 or 3  36km-fin: 2.9±0.5+ 0 or 1 or 3 | Measured values unknown; assumed as average running speed ± SD + ambient wind speed of 0 or 1 or 3 m/s |
| Metabolic rate:  M (ml・kg^-1^・min^-1^) | 0-36km: 44.1±6.0  36km-fin: 38.2±5.9  (VO_2max_: 58.3±5.9) | Avg of measured values ± SD |
| Duration:  D (min) | 207±26 | Avg of measured values ± SD  (Set in conjunction with M) |
| Weight:  W (kg) | 75.7±10.9 | Avg of measured values ± SD |
| Height: H (m) | 1.78±0.07 | Avg of measured values ± SD |
| Age (year) | 36.7±7.5 | Avg of measured values ± SD |
| Sex | Male | Same as the reference value |


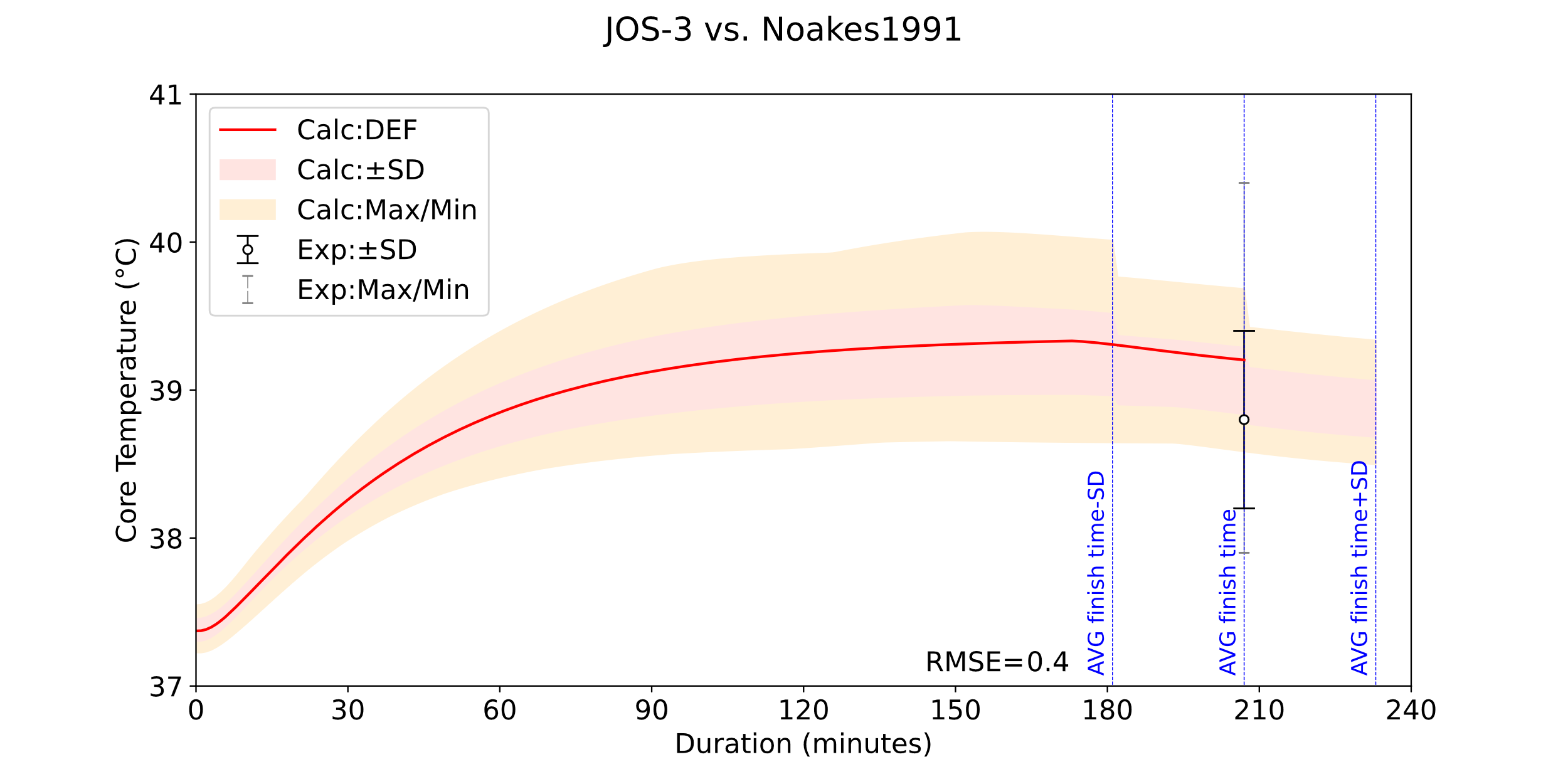


Supplementary Fig. 12 Core temperature reproduced by the joint system thermoregulation model [JOS-3] (case 12: marathon, Noakes et al. (1991), n=30); AVG finish time - SD: finish time for cases with high metabolic rate; AVG finish time: finish time for participants with average metabolic rate; AVG finish time + SD: finish time for participants with low metabolic rate; For the four parameters of height, weight, age, and metabolic rate, three patterns of mean value and mean value ± standard deviation were set, three patterns of temperature trend were set, and three patterns of wind speed (0, 1 and 3 (m/s)) were set, and these have been exhaustively combined, and 729 patterns of calculation were performed.

1. Parameter settings of the joint system thermoregulation model [JOS-3] (case 13: marathon, Coso et al. (2013), n=40)

| Parameter | Setting | Description |
| --- | --- | --- |
| Ambient temperature: Ta (℃) | [Pattern 1] 27  [Pattern 2] 1st half: 24, 2nd half: 30  [Pattern 3] 1st half: 30, 2nd half: 24 | [Pattern 1] Constant at the avg of measured values  [Pattern 2] Avg-SD in the 1st half, Avg+SD in the 2nd half  [Pattern 3] Avg+SD in the 1st half, Avg-SD in the 2nd half |
| Relative humidity: RH (%) | 27 | Avg of measured values |
| Mean radiative temperature: Tr (℃) | [Pattern 1] 47  [Pattern 2] 1st half: 44, 2nd half: 50  [Pattern 3] 1st half: 50, 2nd half: 44 | Measured values unknown; assumed as Ta+20℃ (Daytime) |
| Wind speed: v (m/s) | (2.8~3.5) +0 or 1 or 3  SD+: (3.5~3.9) + 0 or 1 or 3  SD-: (2.1~3.1) + 0 or 1 or 3 | Measured values unknown; assumed as avg of running speed ± SD + ambient wind speed of 0 or 1 or 3 m/s (set approx. every 5 km) |
| Metabolic rate:  M (ml・kg^-1^・min^-1^) | 37.1~45.5  SD+: 45.5~50.4  SD-: 28.7~40.6  (VO_2max_: unknown) | Calculated using the ACSM (2012) formula from the avg speed ± SD, approx. every 5 km |
| Duration: D (min) | 192±33 | Avg of measured values ± SD  (Set in conjunction with M) |
| Weight: W (kg) | 70±9 | Avg of measured values ± SD |
| Height: H (m) | 1.72±0.07 | Avg of measured values ± SD |
| Age (year) | 41±8 | Avg of measured values ± SD |
| Sex | Male | Assumed as male since 85% of the runners were male |


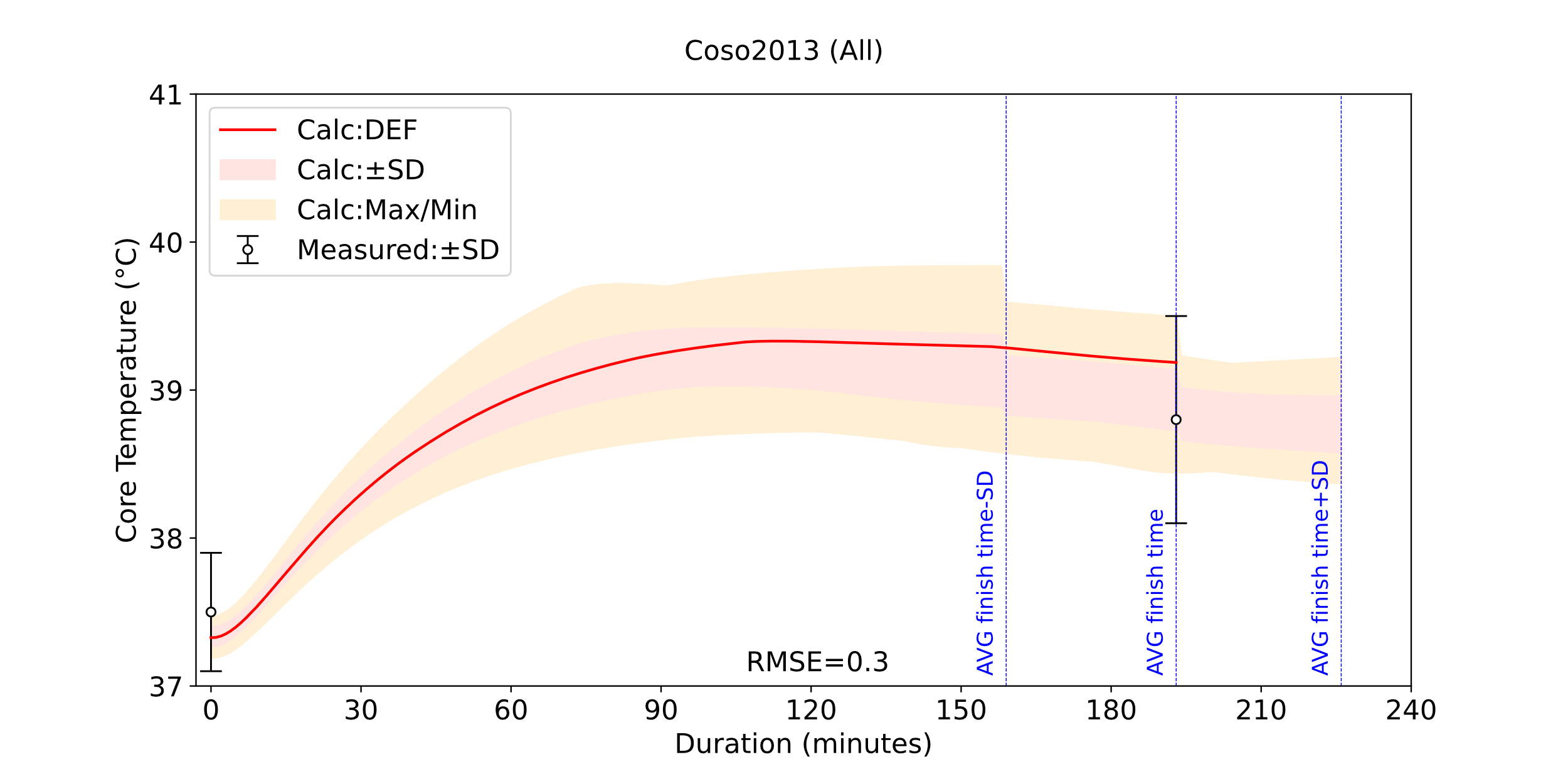


Supplementary Fig. 13 Core temperature reproduced by the joint system thermoregulation model [JOS-3] (case 13: marathon, Coso et al. (2013), n=40); AVG finish time - SD: finish time for participants with high metabolic rate; AVG finish time: finish time for participants with average metabolic rate; AVG finish time + SD: finish time for participants with low metabolic rate; For the four parameters of height, weight, age, and metabolic rate, three patterns of mean value and mean value ± standard deviation were set, three patterns of temperature trend were set, and three patterns of wind speed (0, 1 and 3 (m/s)) were set, and these have been exhaustively combined, and 729 patterns of calculation were performed.

1. Parameter settings of the joint system thermoregulation model [JOS-3] (case 14: marathon, Racinais et al. (2021), female, n=15)

| Parameter | Setting | Description |
| --- | --- | --- |
| Ambient temperature: Ta (℃) | [Pattern 1] 32  [Pattern 2] 1st half: 31.3, 2nd half: 32.7  [Pattern 3] 1st half: 32.7, 2nd half: 31.3 | [Pattern 1] Constant at the avg of measured values  [Pattern 2] Avg-SD in the 1st half, Avg+SD in the 2nd half  [Pattern 3] Avg+SD in the 1st half, Avg-SD in the 2nd half |
| Relative humidity:  RH (%) | 77.9 | Avg of measured values |
| Mean radiative temperature: Tr (℃) | [Pattern 1] 32  [Pattern 2] 1st half: 31.3, 2nd half: 32.7  [Pattern 3] 1st half: 32.7, 2nd half: 31.3 | Measured values unknown; assumed to be same as Ta (Night) |
| Wind speed: v (m/s) | 4.2±0.2+0 or 1 or 3 | Measured direction unknown; assumed as avg of running speed ± SD + ambient wind speed of 0 or 1 or 3 m/s |
| Metabolic rate:  M (ml・kg^-1^・min^-1^) | 50.7 or 53.5 or 56.5  (VO_2max_: unknown) | Calculated using the ACSM (2012) formula from the avg speed ± SD |
| Duration: D (min) | 169±10 | Avg of measured values ± SD  (Set in conjunction with M) |
| Weight: W (kg) | 48.3±4.3 | Avg of measured values ± SD |
| Height: H (m) | 1.62±0.05 | Avg of measured values ± SD |
| Age (year) | 32.6±6.0 | Avg of measured values ± SD |
| Sex | Female | Same as the reference value |


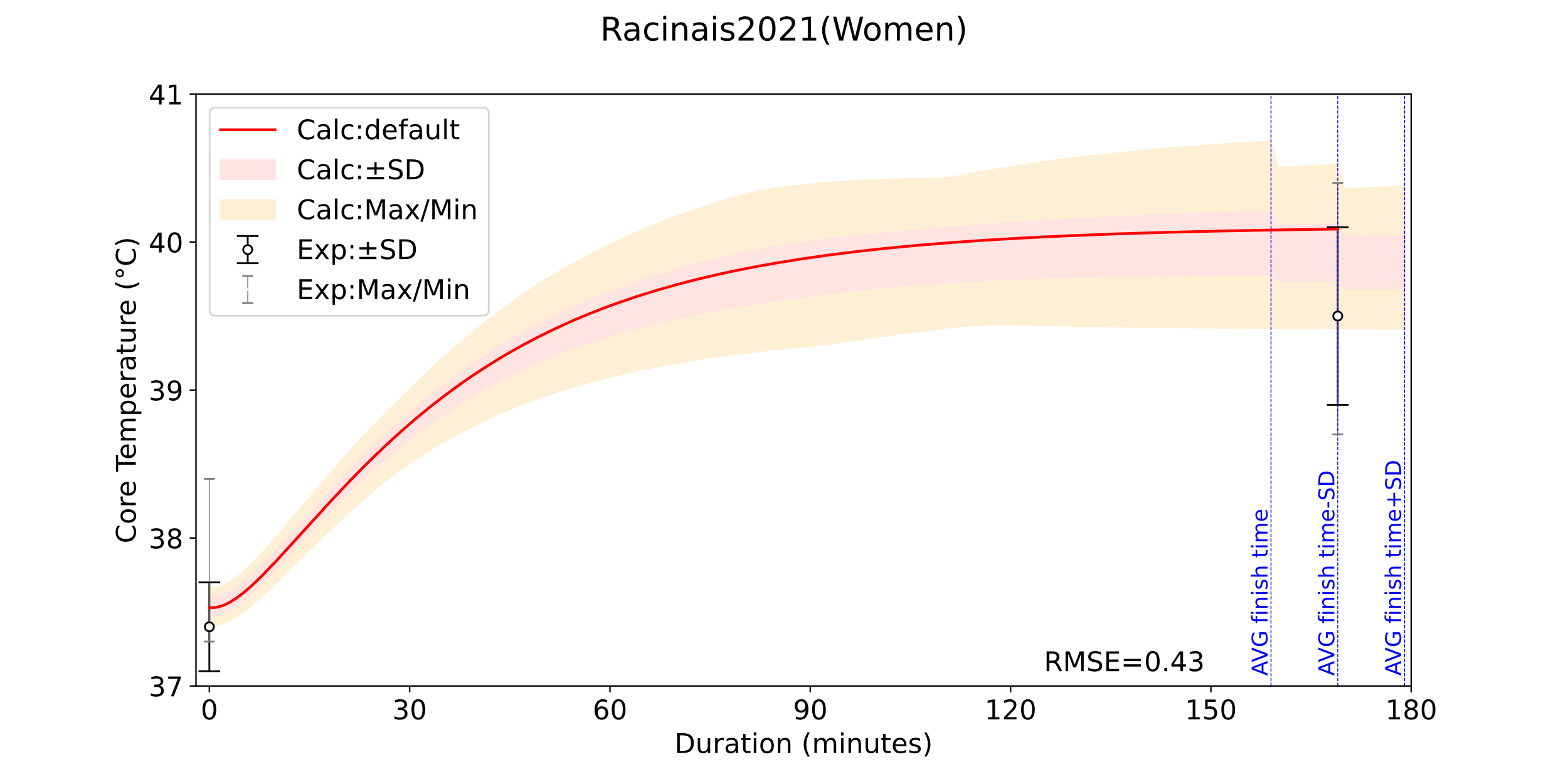


Supplementary Fig. 14 Core temperature reproduced by the joint system thermoregulation model [JOS-3] (case 14: marathon, Racinais et al. (2021), female, n=15); AVG finish time - SD: finish time for participants with high metabolic rate; AVG finish time: finish time for participants with average metabolic rate; AVG finish time + SD: finish time for participants with low metabolic rate; For the four parameters of height, weight, age, and metabolic rate, three patterns of mean value and mean value ± standard deviation were set, three patterns of temperature trend were set, and three patterns of wind speed (0, 1 and 3 (m/s)) were set, and these have been exhaustively combined, and 729 patterns of calculation were performed.

1. Parameter settings of the joint system thermoregulation model [JOS-3] (case 15: marathon, Racinais et al. (2021), male, n=14)

| Parameter | Setting | Description |
| --- | --- | --- |
| Ambient temperature: Ta (℃) | [Pattern 1] 29.3  [Pattern 2] 1st half: 28.8, 2nd half: 29.8  [Pattern 3] 1st half: 29.8, 2nd half: 28.8 | [Pattern 1] Constant at the avg of measured values  [Pattern 2] Avg-SD in the 1st half, Avg+SD in the 2nd half  [Pattern 3] Avg+SD in the 1st half, Avg-SD in the 2nd half |
| Relative humidity:  RH (%) | 46.3 | Avg of measured values |
| Mean radiative temperature: Tr (℃) | [Pattern 1] 29.3  [Pattern 2] 1st half: 28.8, 2nd half: 29.8  [Pattern 3] 1st half: 29.8, 2nd half: 28.8 | Measured values unknown; assumed to be same as Ta (Night) |
| Wind speed: v (m/s) | 5.0±0.2+0 or 1 or 3 | Measured direction unknown; assumed as avg of running speed ± SD + ambient wind speed of 0 or 1 or 3 m/s |
| Metabolic rate:  M (ml・kg^-1^・min^-1^) | 60.5 or 63.3 or 66.5  (VO_2max_: unknown) | Calculated using the ACSM (2012) formula from the avg speed ± SD |
| Duration: D (min) | 141±7 | Avg of measured values ± SD  (Set in conjunction with M) |
| Weight: W (kg) | 60.8±4.5 | Avg of measured values ± SD |
| Height: H (m) | 1.76±0.07 | Avg of measured values ± SD |
| Age (year) | 30.6±3.3 | Avg of measured values ± SD |
| Sex | Male | Same as the reference value |


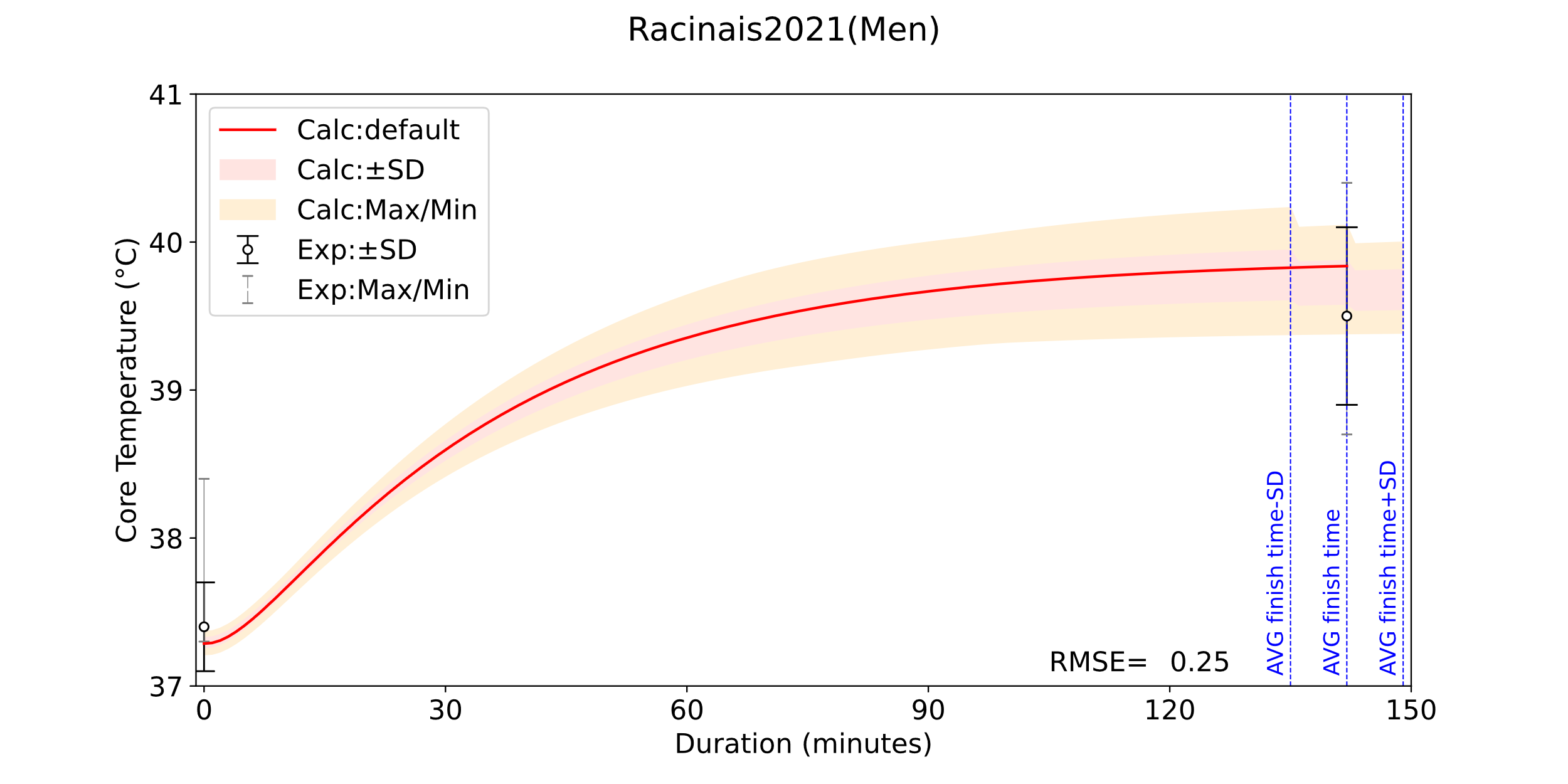


Supplementary Fig. 15 Core temperature reproduced by the joint system thermoregulation model [JOS-3] (case 15: marathon, Racinais et al. (2021), male, n=14); AVG finish time - SD: finish time for participants with high metabolic rate; AVG finish time: finish time for participants with average metabolic rate; AVG finish time + SD: finish time for participants with low metabolic rate; For the four parameters of height, weight, age, and metabolic rate, three patterns of mean value and mean value ± standard deviation were set, three patterns of temperature trend were set, and three patterns of wind speed (0, 1 and 3 (m/s)) were set, and these have been exhaustively combined, and 729 patterns of calculation were performed.

1. Parameter settings of the joint system thermoregulation model [JOS-3] (case 16: football, Edwards and Clark (2006), recreational players, n=8)

| Parameter | Setting | Description |
| --- | --- | --- |
| Ambient temperature:  Ta (℃) | [Pattern 1] 16  [Pattern 2] 1st half: 15, 2nd half: 17  [Pattern 3] 1st half: 17, 2nd half:15  HT: 15 | [Pattern 1] Constant at the avg of measured values  [Pattern 2] Avg-1* in the 1st half, Avg+1* in the 2nd half  [Pattern 3] Avg+1* in the 1st half, Avg-1* in the 2nd half  Assumed as 15 in HT in all patterns  *1 is the SD of Ta of Ozgunen 2010 (Moderate heat) and assumed to be same in this case. |
| Relative humidity:  RH (%) | 47  HT: 50 | Avg of measured values; assumed as 50 in HT |
| Mean radiative temperature:  Tr (℃) | [Pattern 1] 36  [Pattern 2] 1st half: 35, 2nd half: 37  [Pattern 3] 1st half: 37, 2nd half:35  HT: 15 | Measured values unknown; assumed as Ta+20 (Daytime);  assumed as 15 in HT |
| Wind speed:  v (m/s) | 1.8+0 or 1 or 3  HT: 5 | Assumed as typical speed of movement during the match (10 km/90 min) + wind speeds of 0 or 1 or 3 m/s; assumed as 5 in HT |
| Metabolic rate: M (ml・kg^-1^・min^-1^) | 35±3.5  (VO_2max_: 52.73±4.1) | Measured values unknown; set to METs for football in Ainsworth et al. (2011) with a range of ±10% |
| Duration: D (min) | 1st half: 45  HT: 15  2nd half: 45 | Same as the reference value |
| Weight: W (kg) | 81.1±3.9 | Avg of measured values ± SD |
| Height: H (m) | 1.79±0.02 | Avg of measured values ± SD |
| Age (year) | 20.0±2.2 | Avg of measured values ± SD |
| Sex | Male | Same as the reference value |


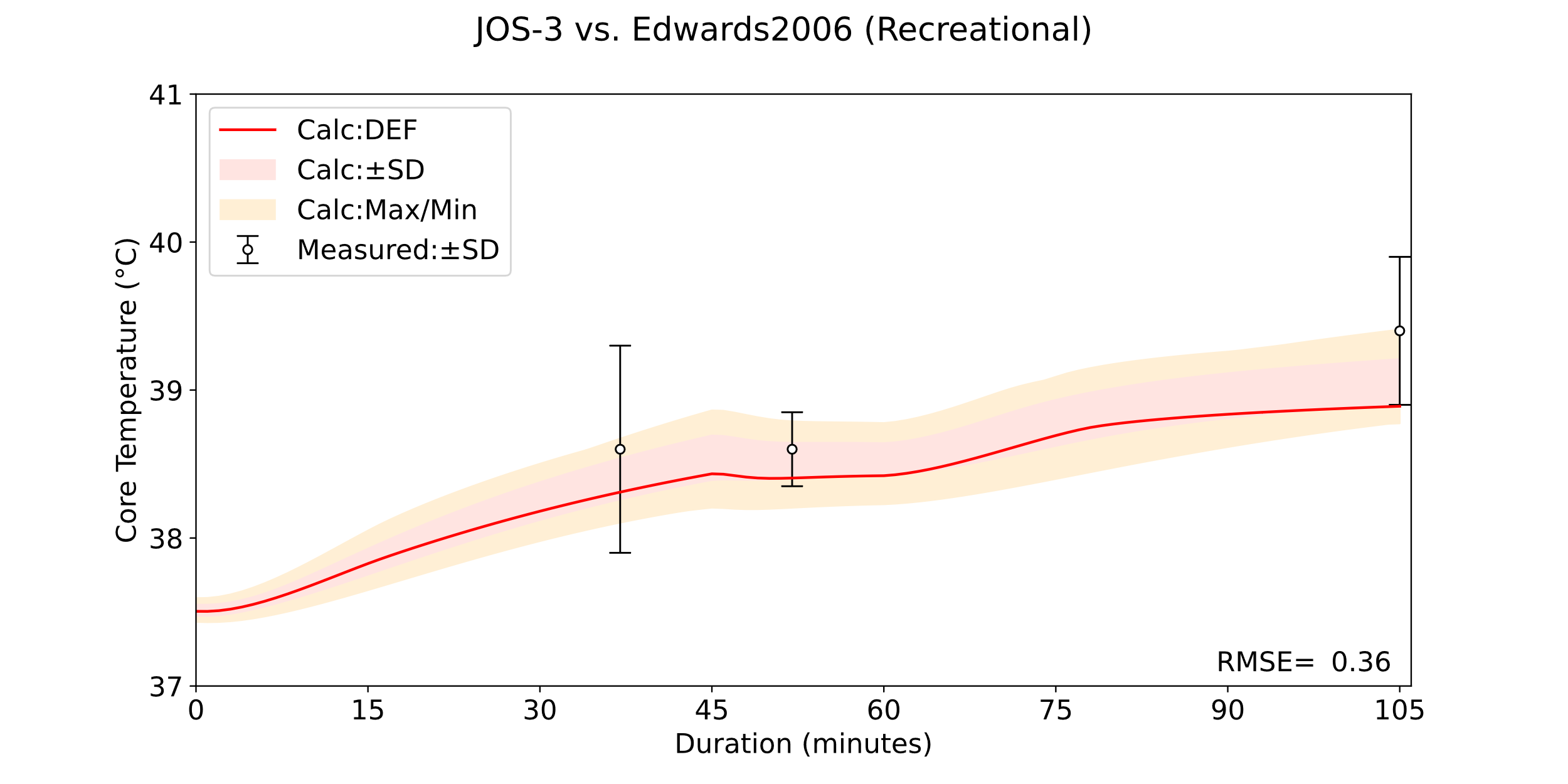


Supplementary Fig. 16 Core temperature reproduced by the joint system thermoregulation model [JOS-3] (case 16: football, Edwards and Clark (2006), recreational players, n=8); For the four parameters of height, weight, age, and metabolic rate, three patterns of mean value and mean value ± standard deviation were set, three patterns of temperature trend were set, and three patterns of wind speed (0, 1 and 3 (m/s)) were set, and these have been exhaustively combined, and 729 patterns of calculation were performed.

1. Parameter settings of the joint system thermoregulation model [JOS-3] (case 17: football, Edwards and Clark (2006), professional players, n=7)

| Parameter | Setting | Description |
| --- | --- | --- |
| Ambient temperature: Ta (℃) | [Pattern 1] 19  [Pattern 2] 1st half:18, 2nd half:20  [Pattern 3] 1st half: 20, 2nd half: 18  HT: 15 | [Pattern 1] Constant at the avg of measured values  [Pattern 2] Avg-1* in the 1st half, Avg+1* in the 2nd half  [Pattern 3] Avg+1* in the 1st half, Avg-1* in the 2nd half  Assumed as 15 in HT in all patterns  *1 is the SD of Ta of Ozgunen 2010 (Moderate heat) and assumed to be same in this case. |
| Relative humidity:  RH (%) | 53  HT: 50 | Avg of measured values; Assumed as 50 in HT |
| Mean radiative temperature: Tr (℃) | [Pattern 1] 19  [Pattern 2] 1st half 18, 2nd half 20  [Pattern 3] 1st half 20, 2nd half 18  HT: 15 | Measured values unknown; assumed to be same as Ta (Night) |
| Wind speed: v (m/s) | 1.8+0 or 1 or 3  HT: 5 | Assumed as typical speed of movement during the match (10 km/90 min) + wind speeds of 0 or 1 or 3 m/s  Assumed as 5 in HT |
| Metabolic rate:  M (ml・kg^-1^・min^-1^) | 35±3.5  (VO_2max_: 65.62±4.9) | Measured values unknown. Set to METs for football in Ainsworth et al. (2011) with a range of ±10% |
| Duration:  D (min) | 1st half: 45  HT: 15  2nd half: 45 | Same as the reference value |
| Weight:  W (kg) | 79.5±2.6 | Avg of measured values ± SD |
| Height: H (m) | 1.79±0.04 | Avg of measured values ± SD |
| Age (year) | 24±3 | Avg of measured values ± SD |
| Sex | Male | Same as the reference value |


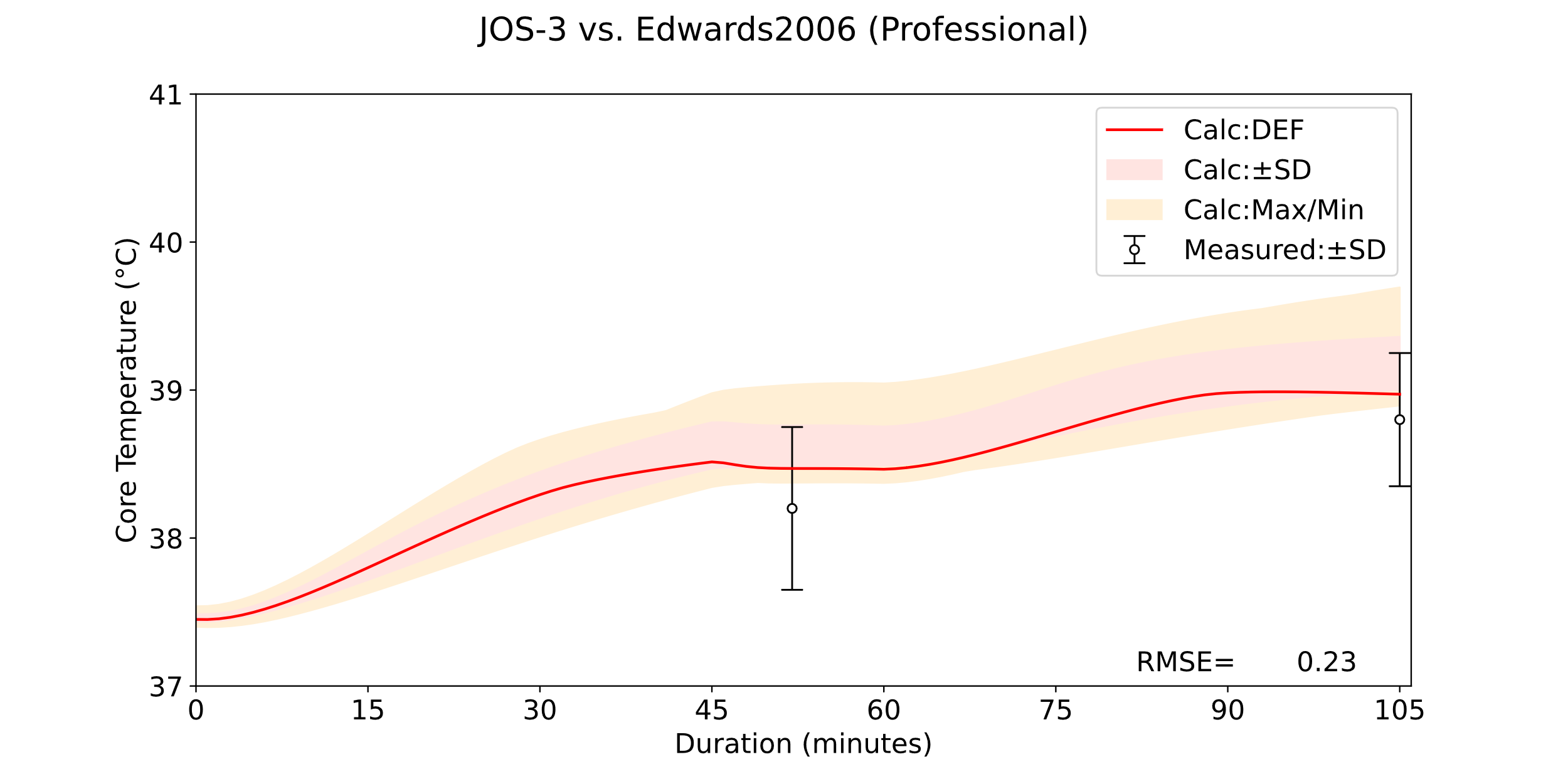


Supplementary Fig. 17 Core temperature reproduced by the joint system thermoregulation model [JOS-3] (case 17: football, Edwards and Clark (2006), professional players, n=7); For the four parameters of height, weight, age, and metabolic rate, three patterns of mean value and mean value ± standard deviation were set, three patterns of temperature trend were set, and three patterns of wind speed (0, 1 and 3 (m/s)) were set, and these have been exhaustively combined, and 729 patterns of calculation were performed.

1. Parameter settings of the joint system thermoregulation model [JOS-3] (case 18: football, Özgünen et al. (2010), moderate heat, n=11)

| Parameter | Setting | Description |
| --- | --- | --- |
| Ambient temperature: Ta (℃) | [Pattern 1] 34  [Pattern 2] 1st half:33, 2nd half:35  [Pattern 3] 1st half: 35, 2nd half: 33  HT: 15 | [Pattern 1] Constant at the avg of measured values  [Pattern 2] Avg-SD in the 1st half, Avg+SD in the 2nd half  [Pattern 3] Avg+SD in the 1st half, Avg-SD in the 2nd half  Assumed as 15 in HT in all patterns |
| Relative humidity: RH (%) | 38 | Avg of measured values |
| Mean radiative temperature: Tr (℃) | [Pattern 1] 54  [Pattern 2] 1st half:53, 2nd half:55  [Pattern 3] 1st half: 55, 2nd half: 53  HT: 15 | Measured values unknown; assumed as Ta+20 (Daytime); assumed as 15 in HT in all patterns |
| Wind speed: v (m/s) | 1.8+0 or 1 or 3  HT: 5 | Assumed as typical speed of movement during the match (10 km/90 min) + wind speeds of 0 or 1 or 3 m/s; assumed as 5 in HT |
| Metabolic rate:  M (ml・kg^-1^・min^-1^) | 35±3.5  (VO_2max_: 62.6±6.8) | Measured values unknown. Set to METs for football in Ainsworth et al. (2011) with a range of ±10% |
| Duration: D (min) | 1st half: 45  HT: 15  2nd half: 45 | Same as the reference value |
| Weight: W (kg) | 68.5±5.3 | Avg of measured values ± SD |
| Height: H (m) | 1.77±0.05 | Avg of measured values ± SD |
| Age (year) | 20.4±2.1 | Avg of measured values ± SD |
| Sex | Male | Same as the reference value |


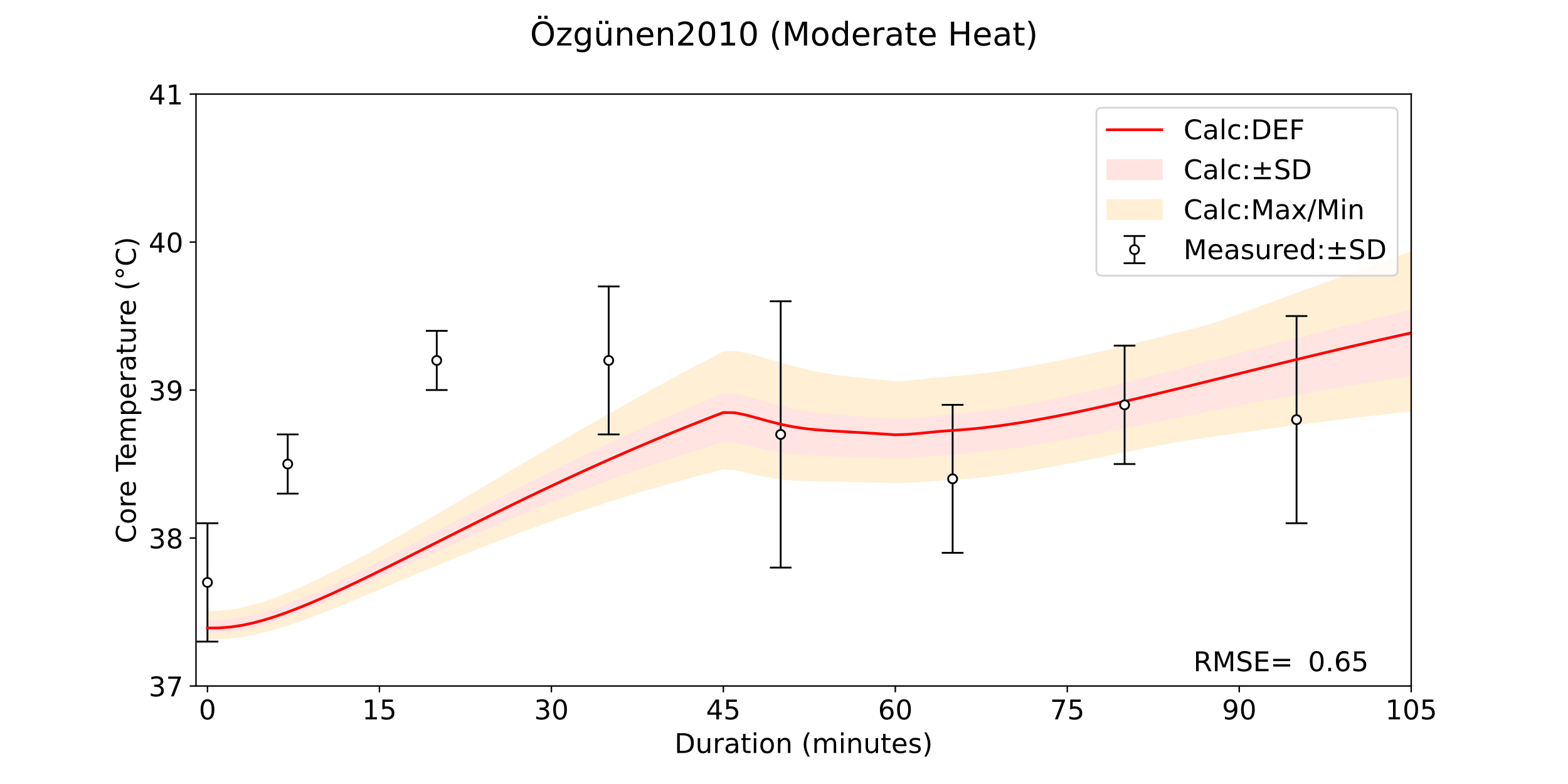


Supplementary Fig. 18 Core temperature reproduced by the joint system thermoregulation model [JOS-3] (case 18: football, Özgünen et al. (2010), moderate heat, n=11); For the four parameters of height, weight, age, and metabolic rate, three patterns of mean value and mean value ± standard deviation were set, three patterns of temperature trend were set, and three patterns of wind speed (0, 1 and 3 (m/s)) were set, and these have been exhaustively combined, and 729 patterns of calculation were performed.

1. Parameter settings of the joint system thermoregulation model [JOS-3] (case 19: football, Özgünen et al. (2010), high heat, n=11)

| Parameter | Setting | Description |
| --- | --- | --- |
| Ambient temperature:  Ta (℃) | 36  HT: 15 | Avg of measured values (SD=0); assumed as 15 in HT |
| Relative humidity: RH (%) | 61 | Avg of measured values |
| Mean radiative temperature:  Tr (℃) | 56  HT: 15 | Measured values unknown; assumed as Ta+20 (Daytime);  assumed as 15 in HT |
| Wind speed:  v (m/s) | 1.8+0 or 1 or 3 | Assumed as typical speed of movement during the match (10 km/90 min) + wind speeds of 0 or 1 or 3 m/s |
| Metabolic rate:  M (ml・kg^-1^・min^-1^) | 1st half: 35.0±3.5  2nd half:  11.7±1.2  (VO_2max_: 62.6±6.8) | Measured values unknown; 1st half: Set to METs for football in Ainsworth et al. (2011) with a range of ±10%. 2nd half: Set to one third of the 1st half so that calculated values match measured values |
| Duration: D (min) | 1st half: 45  HT: 15  2nd half: 45 | Same as the reference value |
| Weight: W (kg) | 68.5±5.3 | Avg of measured values ± SD |
| Height: H (m) | 1.77±0.05 | Avg of measured values ± SD |
| Age (year) | 20.4±2.1 | Avg of measured values ± SD |
| Sex | Male | Same as the reference value |


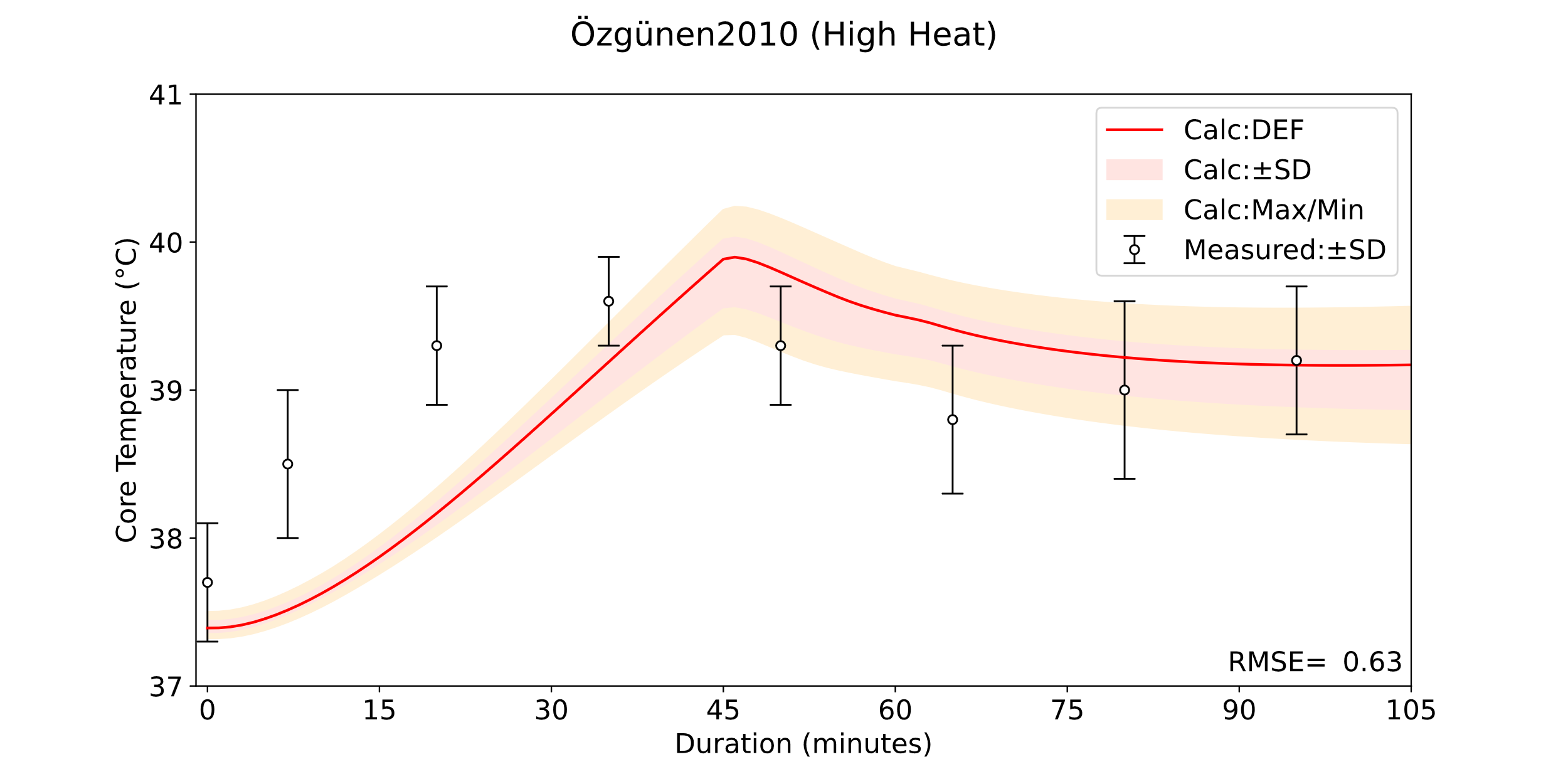


Supplementary Fig. 19 Core temperature reproduced by the joint system thermoregulation model [JOS-3] (case 19: football, Özgünen et al. (2010), high heat, n=11); For the four parameters of height, weight, age, and metabolic rate, three patterns of mean value and mean value ± standard deviation were set, three patterns of temperature trend were set, and three patterns of wind speed (0, 1 and 3 (m/s)) were set, and these have been exhaustively combined, and 729 patterns of calculation were performed.

1. Parameter settings of the joint system thermoregulation model [JOS-3] (case 20: football, Chalmers et al. (2019), high heat, n=12)

| Parameter | Setting | Description |
| --- | --- | --- |
| Ambient temperature: Ta (℃) | [Pattern 1] 35.7  [Pattern 2] 1st half:35.6, 2nd half:35.8  [Pattern 3] 1st half: 35.8, 2nd half: 35.6  HT: 15 | [Pattern 1] Constant at the avg of measured values  [Pattern 2] Avg-SD in the 1st half, Avg+SD in the 2nd half  [Pattern 3] Avg+SD in the 1st half, Avg-SD in the 2nd half  Assumed as 15 in HT in all patterns |
| Relative humidity:  RH (%) | 52.5 | Avg of measured values |
| Mean radiative temperature: Tr (℃) | [Pattern 1] 35.7  [Pattern 2] 1st half:35.6, 2nd half:35.8  [Pattern 3] 1st half: 35.8, 2nd half: 35.6  HT: 15 | Measured values unknown; assumed to be same as Ta (Indoor); Assumed as 15 in HT in all patterns |
| Wind speed: v (m/s) | 1.8+0 or 1 or 3 | Assumed as typical speed of movement during the match (10 km/90 min) + wind speeds of 0 or 1 or 3 m/s |
| Metabolic rate:  M (ml・kg^-1^・min^-1^) | 35±3.5  (VO_2max_: 58±3) | Measured values unknown; set to METs for football in Ainsworth et al. (2011) with a range of ±10% |
| Duration: D (min) | 1st half: 45  HT: 20  2nd half: 45 | Same as the reference value |
| Weight: W (kg) | 75±6 | Avg of measured values ± SD |
| Height: H (m) | 1.79±0.05 | Avg of measured values ± SD |
| Age (year) | 24±7 | Avg of measured values ± SD |
| Sex | Male | Same as the reference value |


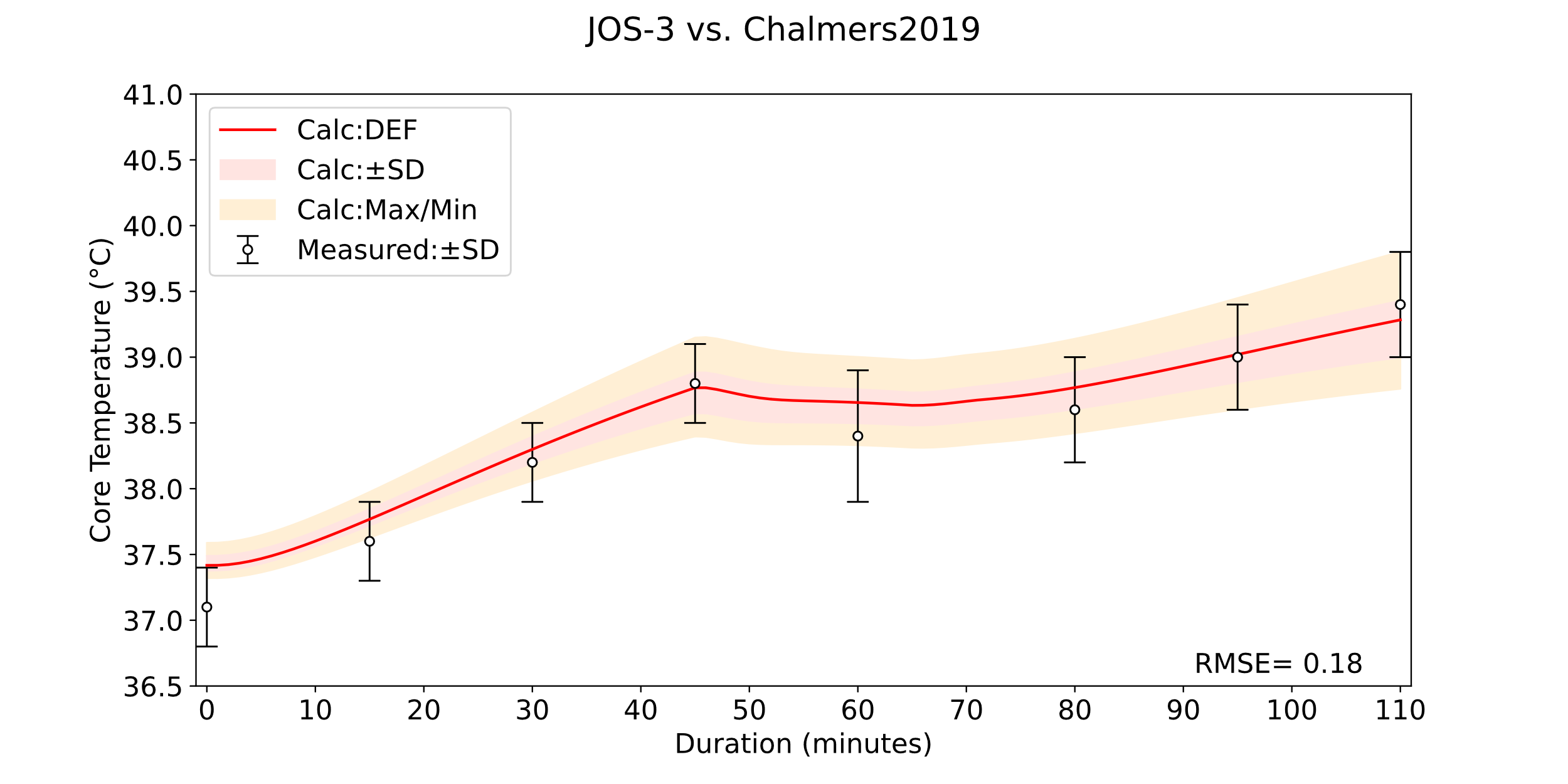


Supplementary Fig. 20 Core temperature reproduced by the joint system thermoregulation model [JOS-3] (case 20: football, Chalmers et al. (2019), high heat, n=12); For the four parameters of height, weight, age, and metabolic rate, three patterns of mean value and mean value ± standard deviation were set, three patterns of temperature trend were set, and three patterns of wind speed (0, 1 and 3 (m/s)) were set, and these have been exhaustively combined, and 729 patterns of calculation were performed.

1. Parameter settings of the joint system thermoregulation model [JOS-3] (case 21: rowing, Taylor et al. (2014), cool, n=8)

| Parameter | Setting | Description |
| --- | --- | --- |
| Ambient temperature: Ta (℃) | 20 | Avg of measured values |
| Relative humidity:  RH (%) | 40 | Avg of measured values |
| Mean radiative temperature: Tr (℃) | 20 | Measured values unknown; assumed to be same as Ta (Indoor) |
| Wind speed: v (m/s) | 0 | Measured values unknown; assumed to be 0 |
| Metabolic rate:  M (ml・kg^-1^・min^-1^) | 43.8±4.4  (VO_2max_: unknown) | Measured values unknown; Set to METs for competitive rowing in Ainsworth et al. (2011) with a range of ±10% |
| Duration: D (min) | 9 | Same as the reference value |
| Weight: W (kg) | 66.8±3.1 | Avg of measured values ± SD |
| Height: H (m) | 1.66±0.04 | Avg of measured values ± SD |
| Age (year) | 19.9±1.5 | Avg of measured values ± SD |
| Sex | Female | Same as the reference value |


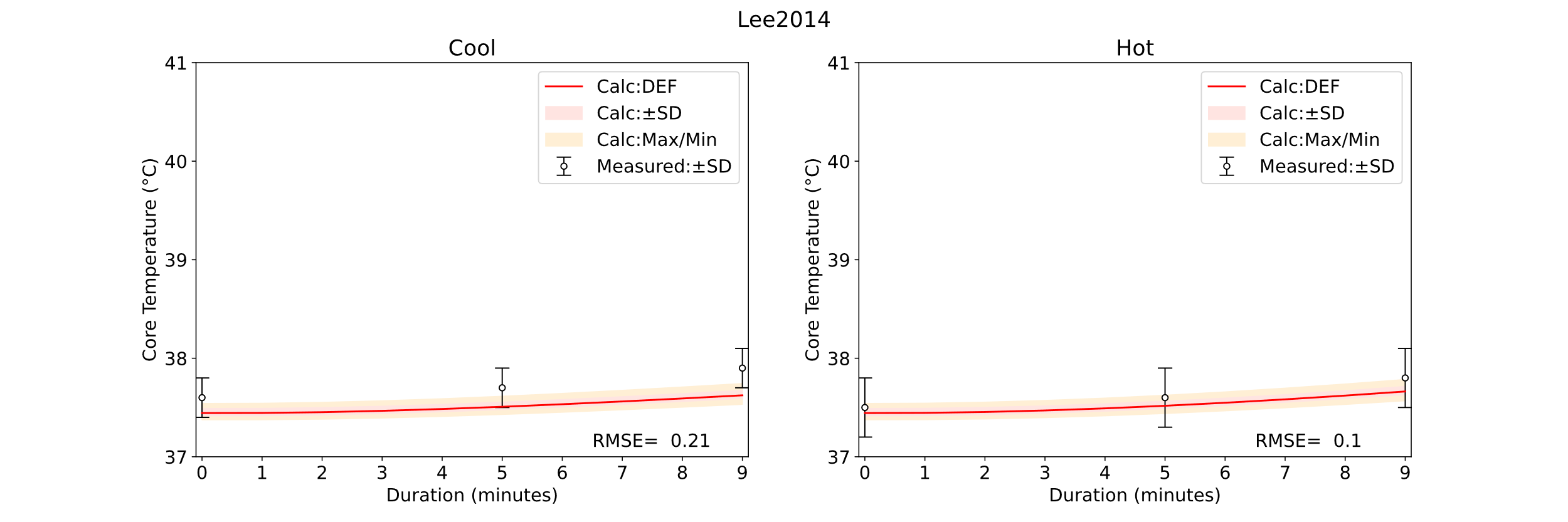


Supplementary Fig. 21 Core temperature reproduced by the joint system thermoregulation model [JOS-3] (case 21: rowing, Taylor et al. (2014), cool, n=8); For the four parameters of height, weight, age, and metabolic rate, three patterns of mean values and mean ± standard deviation were set and exhaustively combined, resulting in 81 calculation patterns.

1. Parameter settings of the joint system thermoregulation model [JOS-3] (case 22: rowing, Taylor et al. (2014), hot, n=8)

| Parameter | Setting | Description |
| --- | --- | --- |
| Ambient temperature: Ta (℃) | 35 | Avg of measured values |
| Relative humidity:  RH (%) | 60 | Avg of measured values |
| Mean radiative temperature: Tr (℃) | 35 | Measured values unknown; assumed to be same as Ta (Indoor) |
| Wind speed: v (m/s) | 0 | Measured values unknown; assumed to be 0 |
| Metabolic rate:  M (ml・kg^-1^・min^-1^) | 43.8±4.4  (VO_2max_: unknown) | Measured values unknown; set to METs for competitive rowing in Ainsworth et al. (2011) with a range of ±10% |
| Duration: D (min) | 9 | Same as the reference value |
| Weight: W (kg) | 66.8±3.1 | Avg of measured values ± SD |
| Height: H (m) | 1.66±0.04 | Avg of measured values ± SD |
| Age (year) | 19.9±1.5 | Avg of measured values ± SD |
| Sex | Female | Same as the reference value |


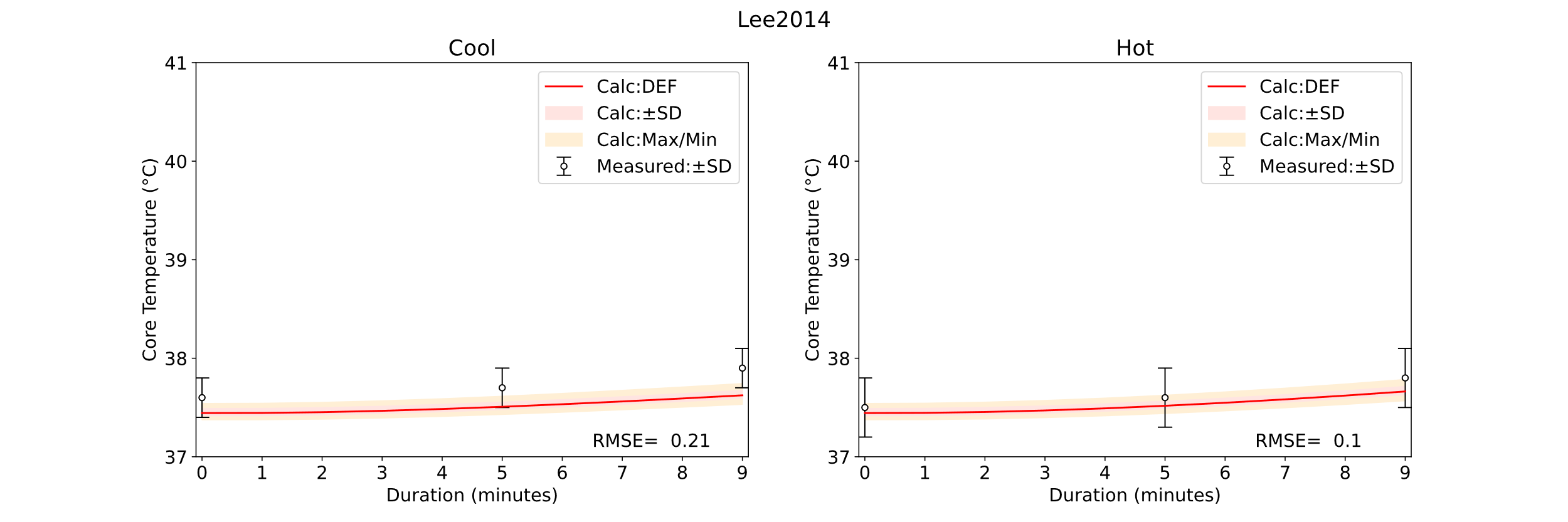


Supplementary Fig. 22 Core temperature reproduced by the joint system thermoregulation model [JOS-3] (case 22: rowing, Taylor et al. (2014), hot, n=8); For the four parameters of height, weight, age, and metabolic rate, three patterns of mean values and mean ± standard deviation were set and exhaustively combined, resulting in 81 calculation patterns.

1. Parameter settings of the joint system thermoregulation model [JOS-3] (case 23: rugby sevens, Fenemor et al. (2021), day 1, n=11)

| Parameter | Setting | Description |
| --- | --- | --- |
| Ambient temperature: Ta (℃) | Game1 (incl. warm-up): 31.1  Game2 (incl. warm-up): 29.0  Preparation: 15.0 | Avg of measured values; assumed as 15.0 in preparation time |
| Relative humidity:  RH (%) | Game1 (incl. warm-up): 71  Game2 (incl. warm-up): 73  Preparation: 50 | Avg of measured values; assumed as 50 in preparation time |
| Mean radiative temperature: Tr (℃) | Daytime: Ta+20  Night: Ta  Preparation: 15.0 | Measured values unknown; assumed as Ta+20 (daytime), or same as Ta (night) |
| Wind speed:  v (m/s) | Warm-up: 1.2+0 or 1 or 3  Game: 1.7+ 0 or 1 or 3  Preparation: 1 | Measured values unknown; assumed as typical speed of movement during the warm-up (70m/min), game (1400m/14min) + wind speeds of 0 or 1 or 3 m/s; assumed as 1 in preparation time |
| Metabolic rate: M (ml・kg^-1^・min^-1^) | Warm-up: 29.1±2.9  Game: 38.7±3.9  Other: 8.8±0.9  (VO_2max_: unknown) | Warm-up: set to METs of rugby from Ainsworth et al. (2011) with a range of ±10%; assuming a load equivalent to 15-man rugby from Taylor et al. (2019)  Game: set to 133% of the METs for 15-man rugby with a range of ±10% as the typical 7-man Rugby movement speed is 133% of that of 15-man Rugby |
| Duration:  D (min) | 243 | Same as the reference value |
| Weight: W (kg) | 94.3±7.5 | Avg of measured values ± SD |
| Height: H (m) | 1.87±0.05 | Avg of measured values ± SD |
| Age (year) | 24±3 | Avg of measured values ± SD |
| Sex | male | Same as the reference value |


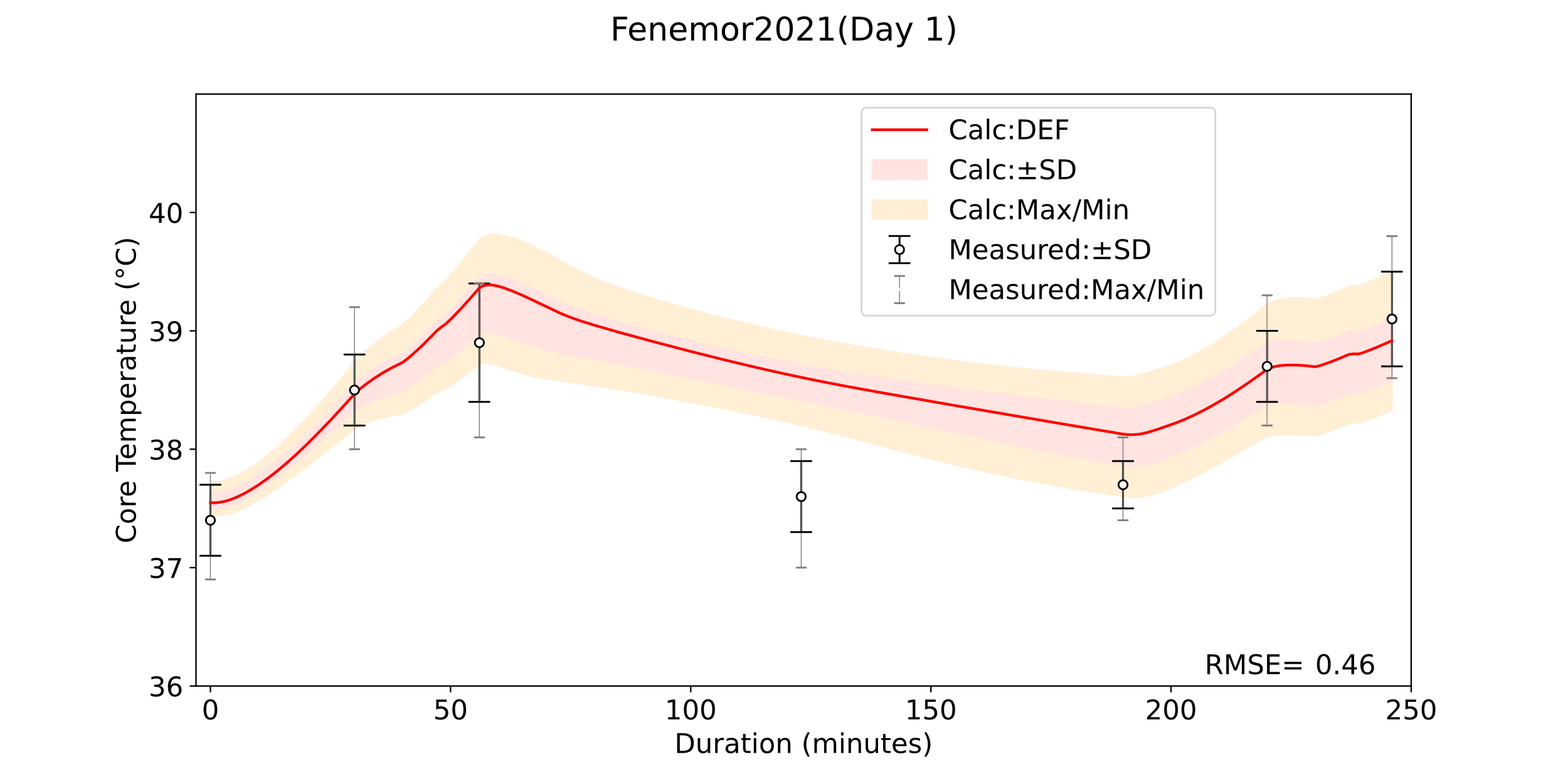


Supplementary Fig. 23 Core temperature reproduced by the joint system thermoregulation model [JOS-3] (case 23: rugby sevens, Fenemor et al. (2021), day 1, n=11); For the four parameters of height, weight, age, and metabolic rate, three patterns of mean value and mean value ± standard deviation were set, and three patterns of wind speed (0, 1 and 3 (m/s)) were set, and these have been exhaustively combined, and 243 patterns of calculation were performed.

1. Parameter settings of the joint system thermoregulation model [JOS-3] (case 24: rugby sevens, Fenemor et al. (2021), day 2, n=11)

| Parameter | Setting | Description |
| --- | --- | --- |
| Ambient temperature:  Ta (℃) | Game 3 (incl. warm-up): 30.4  Game 4 (incl. warm-up): 29.9  Game 5 (incl. warm-up): 26.0  Preparation: 15.0 | Avg of measured values; assumed as 15.0 in preparation time |
| Relative humidity: RH (%) | Game 3 (incl. warm-up): 73  Game 4 (incl. warm-up): 75  Game 5 (incl. warm-up): 81  Preparation: 50 | Avg of measured values; assumed as 50 in preparation time |
| Mean radiative temperature:  Tr (℃) | Daytime: Ta+20  Night: Ta  Preparation: 15.0 | Measured values unknown; assumed as Ta + 20 (daytime), or same as Ta (night) |
| Wind speed:  v (m/s) | Warm-up: 1.2+0 or 1 or 3  Game: 1.7+0 or 1 or 3  Preparation: 1 | Measured values unknown; assumed as typical speed of movement during the warm-up (70m/min), game (1400m/14min) + wind speeds of 0 or 1 or 3 m/s; assumed as 1 in preparation time |
| Metabolic rate: M (ml・kg^-1^・min^-1^) | Warm-up: 29.1±2.9  Game: 38.7±3.9  Other: 8.8±0.9  (VO_2max_: unknown) | Warm-up: set to METs of rugby from Ainsworth et al. (2011) with a range of ±10%; assuming a load equivalent to 15-man rugby from Taylor et al. (2019)  Game: set to 133% of the METs for 15-man rugby with a range of ±10%, as the typical 7-man Rugby movement speed is 133% of that of 15-man Rugby |
| Duration: D (min) | 534 | Same as the reference value |
| Weight: W (kg) | 94.3±7.5 | Avg of measured values ± SD |
| Height: H (m) | 1.87±0.05 | Avg of measured values ± SD |
| Age (year) | 24±3 | Avg of measured values ± SD |
| Sex | Male | Same as the reference value |


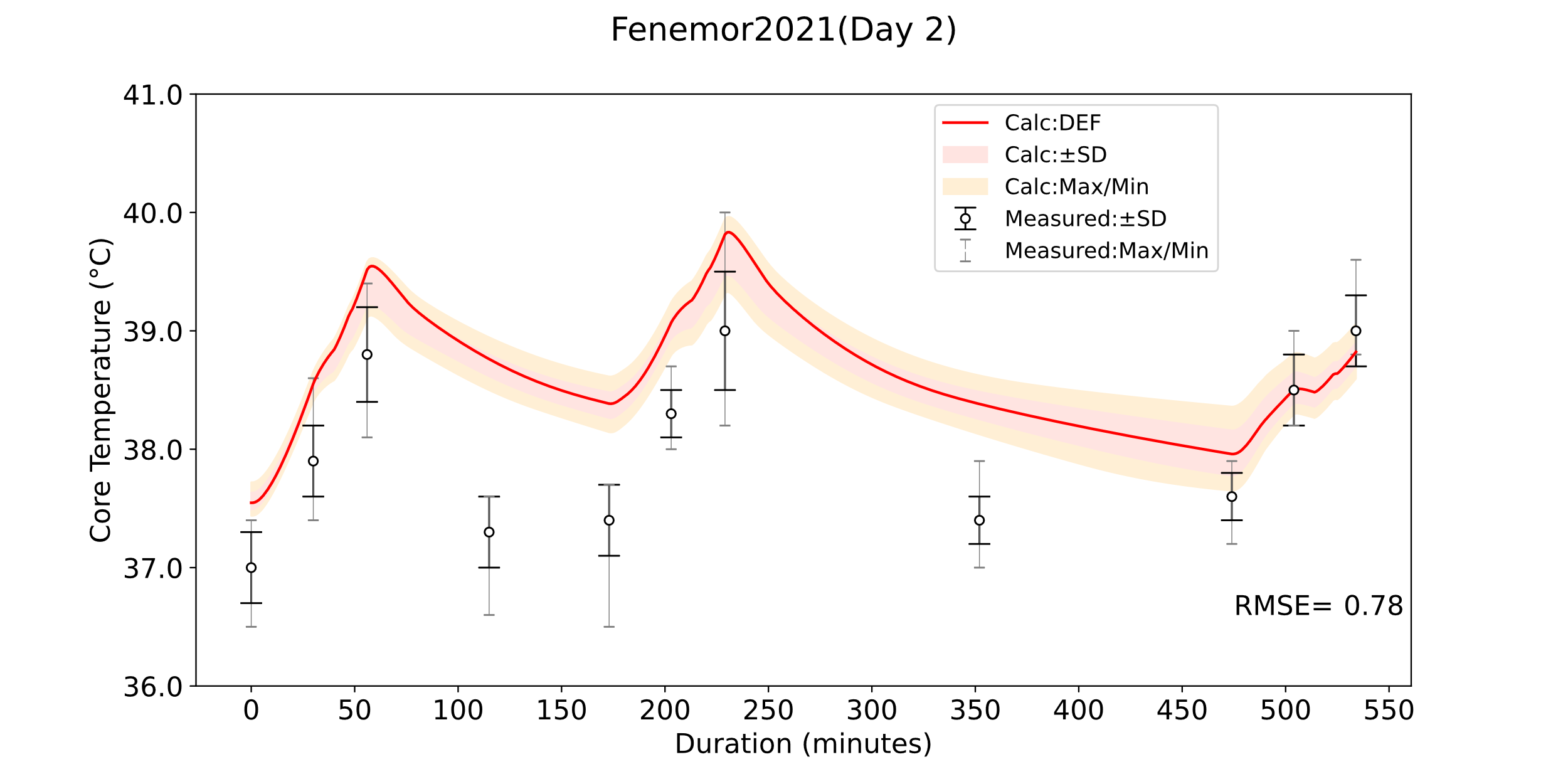


Supplementary Fig. 24 Core temperature reproduced by the joint system thermoregulation model [JOS-3] (case 24: rugby sevens, Fenemor et al. (2021), day 2, n=11); For the four parameters of height, weight, age, and metabolic rate, three patterns of mean value and mean value ± standard deviation were set, and three patterns of wind speed (0, 1 and 3 (m/s)) were set, and these have been exhaustively combined, and 243 patterns of calculation were performed.

1. Parameter settings of the joint system thermoregulation model [JOS-3] (case 25: tennis, Schranner et al. (2017), n=9)

| Parameter | Setting | Description |
| --- | --- | --- |
| Ambient temperature: Ta (℃) | [Pattern 1] 36.5  [Pattern 2] 1st half:36.1, 2nd half:36.9  [Pattern 3] 1st half: 36.9, 2nd half: 36.1 | [Pattern 1] Constant at the avg of measured values  [Pattern 2] Avg-SD in the 1st half, Avg+SD in the 2nd half  [Pattern 3] Avg+SD in the 1st half, Avg-SD in the 2nd half |
| Relative humidity:  RH (%) | 51 | Avg of measured values |
| Mean radiative temperature: Tr (℃) | [Pattern 1] 36.5  [Pattern 2] 1st half:36.1, 2nd half:36.9  [Pattern 3] 1st half: 36.9, 2nd half: 36.1 | Measured values unknown; assumed to be same as Ta (indoor) |
| Wind speed: v (m/s) | 0 | Measured values unknown; assumed to be 0 |
| Metabolic rate:  M (ml・kg^-1^・min^-1^) | Running, acceleration,8km/h: 30.1±2.1  Running, 16km/h: 56.7±4.0  Running, deceleration, 9km/h: 33.6±2.4  Walking, 2km/h: 7.0±0.7  Standing: 6.3±0.6  Break: 5.3±0.5  (VO_2max_: 50.6±6.3) | Running: calculated from the avg. speed using the ACSM (2012) formula with a range of ±7%  Walking, Standing and Break: set to the relevant METs of Ainsworth et al. (2011) with a range of ±10% |
| Duration: D (min) | 94 | Same as the reference value |
| Weight: W (kg) | 77.3±6.7 | Avg of measured values ± SD |
| Height: H (m) | 1.79±0.06 | Avg of measured values ± SD |
| Age (year) | 25±4 | Avg of measured values ± SD |
| Sex | Male | Same as the reference value |


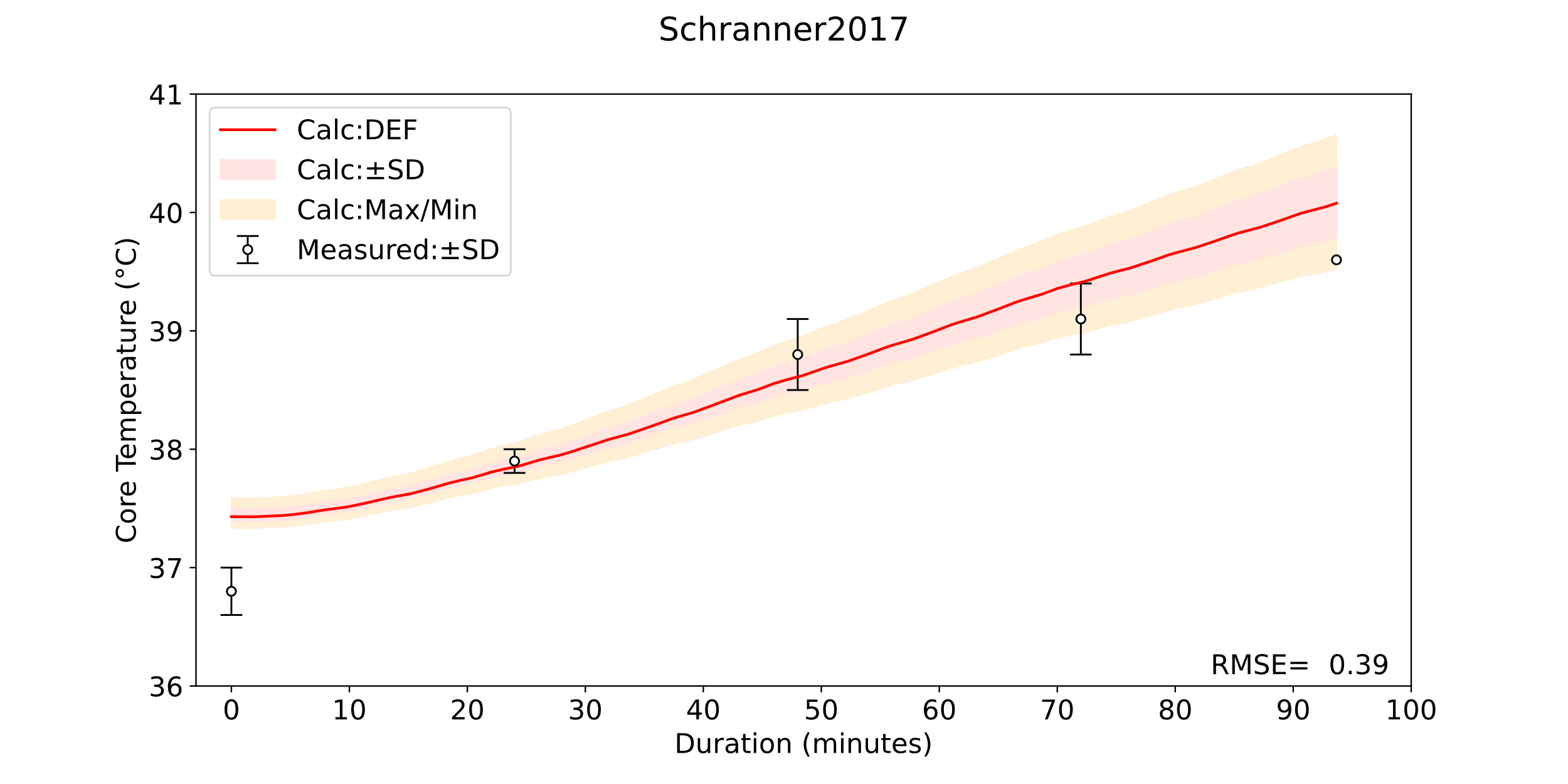


Supplementary Fig. 25 Core temperature reproduced by the joint system thermoregulation model [JOS-3] (case 25: tennis, Schranner et al. (2017), n=9); Three patterns of mean and mean ± standard deviation were set for the four parameters of height, weight, age, and metabolic rate, and three patterns for temperature trends, which were exhaustively combined, resulting in 243 calculation patterns.

1. Parameter settings of the joint system thermoregulation model [JOS-3] (case 26: tennis, Lynch et al. (2018), n=9)

| Parameter | Setting | Description |
| --- | --- | --- |
| Ambient temperature:  Ta (℃) | [Pattern 1] 45  [Pattern 2] 1st half:44, 2nd half:46  [Pattern 3] 1st half: 46, 2nd half: 44 | [Pattern 1] Constant at the avg of measured values  [Pattern 2] Avg-SD in the 1st half, Avg+SD in the 2nd half  [Pattern 3] Avg+SD in the 1st half, Avg-SD in the 2nd half |
| Relative humidity:  RH (%) | 9 | Avg of measured values |
| Mean radiative temperature:  Tr (℃) | [Pattern 1] 45  [Pattern 2] 1st half:44, 2nd half:46  [Pattern 3] 1st half: 46, 2nd half: 44 | Measured values unknown; assumed to be same as Ta (indoor) |
| Wind speed:  v (m/s) | 0.2 | Same as the reference value |
| Metabolic rate: M (ml・kg^-1^・min^-1^) | Exercise: 26.7±1.7  Recovery: 17.5 ±1.8  Break: 5.3 ±0.5  (VO_2max_: 51.7±4.6) | Exercise: Avg of measured values ± SD  Recovery and Break: set to the relevant METs of Ainsworth et al. (2011) with a ±10% range |
| Duration:  D (min) | 113 | Same as the reference value |
| Weight: W (kg) | 73.9±5.0 | Avg of measured values ± SD |
| Height: H (m) | 1.79±0.05 | Avg of measured values ± SD |
| Age (year) | 22±3 | Avg of measured values ± SD |
| Sex | Male | Same as the reference value |


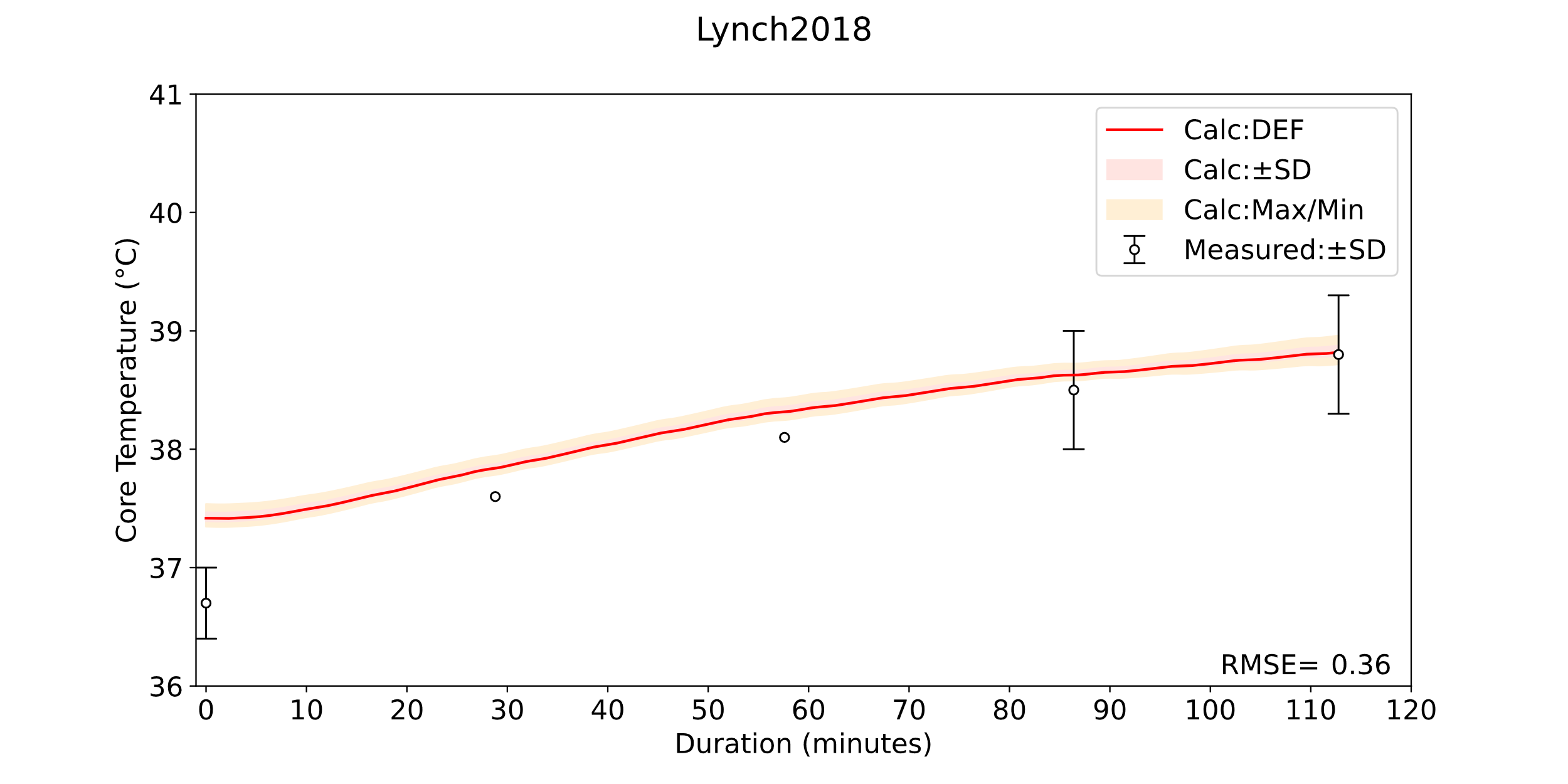


Supplementary Fig. 26 Core temperature reproduced by the joint system thermoregulation model [JOS-3] (case 26: tennis, Lynch et al. (2018), n=9); Three patterns of mean and mean ± standard deviation were set for the four parameters of height, weight, age, and metabolic rate, and three patterns for temperature trends, which were exhaustively combined, resulting in 243 calculation patterns.

1. Parameter settings of the joint system thermoregulation model [JOS-3] (case 27: tennis, Naito et al. (2018), n=7)

| Parameter | Setting | Description |
| --- | --- | --- |
| Ambient temperature: Ta (℃) | [Pattern 1] 36.5  [Pattern 2] 1st half:36, 2nd half:37  [Pattern 3] 1st half: 37, 2nd half: 36 | [Pattern 1] Constant at the avg of measured values  [Pattern 2] Avg-SD in the 1st half, Avg+SD in the 2nd half  [Pattern 3] Avg+SD in the 1st half, Avg-SD in the 2nd half |
| Relative humidity:  RH (%) | 50 | Avg of measured values |
| Mean radiative temperature: Tr (℃) | [Pattern 1] 36.5  [Pattern 2] 1st half:36, 2nd half:37  [Pattern 3] 1st half: 37, 2nd half: 36 | Measured values unknown; assumed to be same as Ta (indoor) |
| Wind speed:  v (m/s) | 0 | Measured values unknown; assumed to be 0 |
| Metabolic rate: M (ml・kg^-1^・min^-1^) | Running, 16km/h: 56.7±4.0  Running, deceleration, 9km/h: 33.6±2.4  Walking, 2km/h: 7.0±0.7  Rest: 6.3±0.6  Break: 5.3±0.5  (VO_2max_: unknown) | Running: calculated from the avg. speed using the ACSM (2012) formula with a range of ±7%  Walking, Standing and Break: set to the relevant METs of Ainsworth et al. (2011) with a range of ±10% |
| Duration:  D (min) | 81 | Same as the reference value |
| Weight: W (kg) | 64.8±6.8 | Avg of measured values ± SD |
| Height: H (m) | 1.72±0.08 | Avg of measured values ± SD |
| Age (year) | 22±2 | Avg of measured values ± SD |
| Sex | male | Same as the reference value |


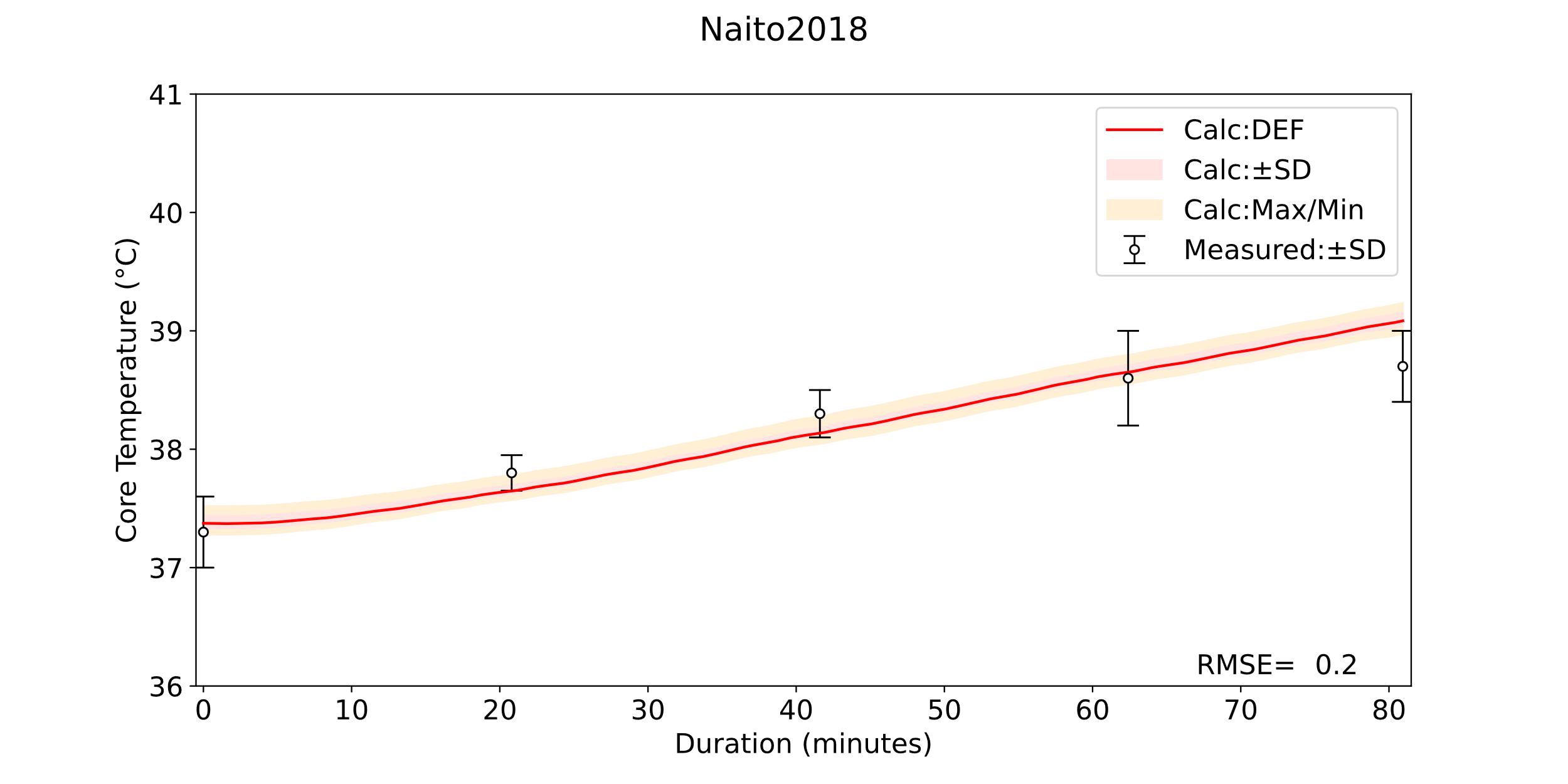


Supplementary Fig. 27 Core temperature reproduced by the joint system thermoregulation model [JOS-3] (case 27: tennis, Naito et al. (2018), n=7); Three patterns of mean and mean ± standard deviation were set for the four parameters of height, weight, age, and metabolic rate, and three patterns for temperature trends, which were exhaustively combined, resulting in 243 calculation patterns.

1. Parameter settings of the joint system thermoregulation model [JOS-3] (case 28: triathlon (only cycling and running), Chan et al. (2008), moderate, n=7)

| Parameter | Setting | Description |
| --- | --- | --- |
| Ambient temperature: Ta (℃) | [Pattern 1] 22.0  [Pattern 2] Cycling:21.8, Running:22.2  [Pattern 3] Cycling: 22.2, Running: 21.8 | [Pattern 1] Constant at the avg of measured values  [Pattern 2] Avg-SD in cycling, Avg+SD in running  [Pattern 3] Avg+SD in cycling, Avg-SD in running |
| Relative humidity: RH (%) | 76.0 | Avg of measured values |
| Mean radiative temperature: Tr (℃) | [Pattern 1] 22.0  [Pattern 2] Cycling:21.8, Running:22.2  [Pattern 3] Cycling: 22.2, Running: 21.8 | Measured values unknown; assumed to be same as Ta (indoor) |
| Wind speed: v (m/s) | Cycling: 9.8  Running: 3.3 | Measured values unknown; Wind speed reproduced with a fan; assumed to be the same as avg. running speed |
| Metabolic rate:  M (ml・kg^-1^・min^-1^) | Cycling: 45.5±4.6  Running: 42.0±4.2  (VO_2max_: 62.2±2.2) | Measured values unknown; Set based on METs for cycling and running of Ainsworth et al. (2011) adjusted, so that calculated core temperature match measured temperature with a range of ±10% |
| Duration:  D (min) | Cycling: 68±3  Running: 51±4 | Avg of measured values ± SD  (Set in conjunction with M) |
| Weight:  W (kg) | 63.7±2.3 | Avg of measured values ± SD |
| Height:  H (m) | 1.65±0.02 | Measured values unknown; estimated from height |
| Age (year) | 26.1±1.6 | Avg of measured values ± SD |
| Sex | Male | Same as the reference value |


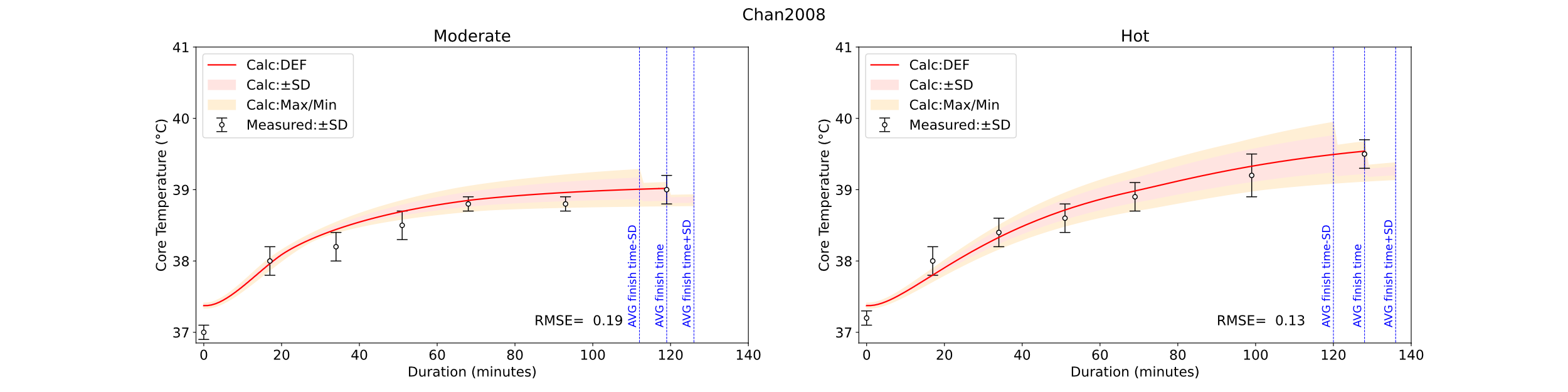


Supplementary Fig. 28 Core temperature reproduced by the joint system thermoregulation model [JOS-3] (case 28: triathlon (only cycling and running), Chan et al. (2008), moderate, n=7); AVG finish time - SD: finish time for participants with high metabolic rate; AVG finish time: finish time for participants with average metabolic rate; AVG finish time + SD: finish time for participants with low metabolic rate. Three patterns of mean and mean ± standard deviation were set for the four parameters of height, weight, age, and metabolic rate, and three patterns for temperature trends, which were exhaustively combined, resulting in 243 calculation patterns.

1. Parameter settings of the joint system thermoregulation model [JOS-3] (case 29: triathlon (only cycling and running), Chan et al. (2008), hot, n=7)

| Parameter | Setting | Description |
| --- | --- | --- |
| Ambient temperature: Ta (℃) | [Pattern 1] 31.2  [Pattern 2] Cycling: 31.0, Running: 31.4  [Pattern 3] Cycling: 31.4, Running: 31.0 | [Pattern 1] Constant at the avg of measured values  [Pattern 2] Avg-SD in cycling, Avg+SD in running  [Pattern 3] Avg+SD in cycling, Avg-SD in running |
| Relative humidity: RH (%) | 76.4 | Avg of measured values |
| Mean radiative temperature:  Tr (℃) | [Pattern 1] 31.2  [Pattern 2] Cycling: 31.0, Running: 31.4  [Pattern 3] Cycling: 31.4, Running: 31.0 | Measured values unknown; assumed to be same as Ta (indoor) |
| Wind speed:  v (m/s) | Cycling: 9.7  Running: 2.8 | Measured values unknown; wind speed reproduced with a fan. Assumed to be the same as avg. running speed |
| Metabolic rate:  M (ml・kg^-1^・min^-1^) | Cycling: 45.5±4.6  Running: 42.0±4.2  (VO_2max_: 62.2±2.2) | Measured values unknown; set based on METs for cycling and running of Ainsworth et al. (2011) adjusted with a range of ±10%, so that calculated core temperature match measured temperature |
| Duration: D (min) | Cycling: 69±3  Running: 59±5 | Avg of measured values ± SD  (Set in conjunction with M) |
| Weight: W (kg) | 63.7±2.3 | Avg of measured values ± SD |
| Height: H (m) | 1.65±0.02 | Measured values unknown; estimated from height |
| Age (year) | 26.1±1.6 | Avg of measured values ± SD |
| Sex | Male | Same as the reference value |


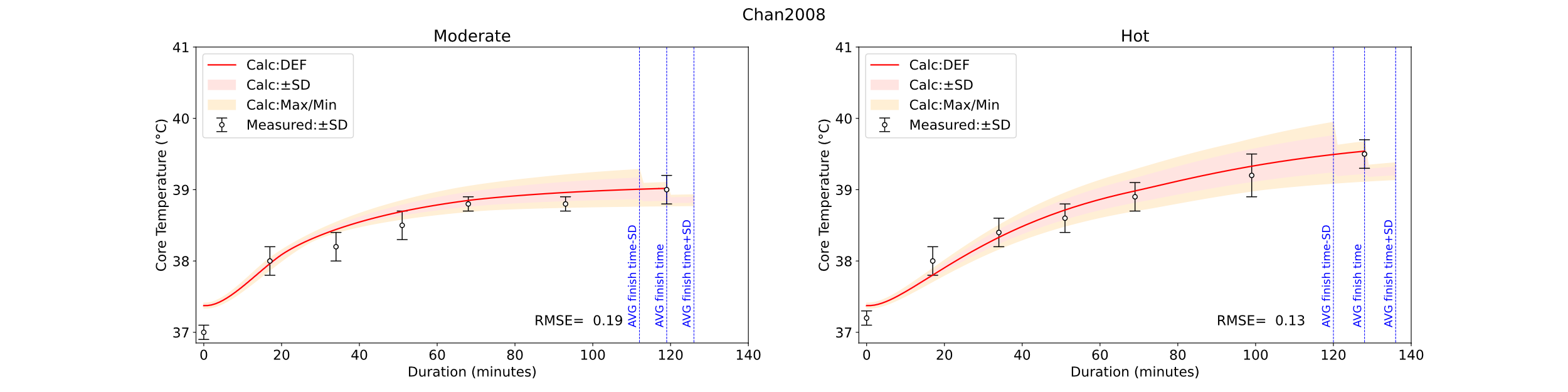


Supplementary Fig. 29 Core temperature reproduced by the joint system thermoregulation model [JOS-3] (case 29: triathlon (only cycling and running), Chan et al. (2008), hot, n=7); AVG finish time - SD: finish time for participants with high metabolic rate; AVG finish time: finish time for participants with average metabolic rate; AVG finish time + SD: finish time for participants with low metabolic rate. Three patterns of mean and mean ± standard deviation were set for the four parameters of height, weight, age, and metabolic rate, and three patterns for temperature trends, which were exhaustively combined, resulting in 243 calculation patterns.


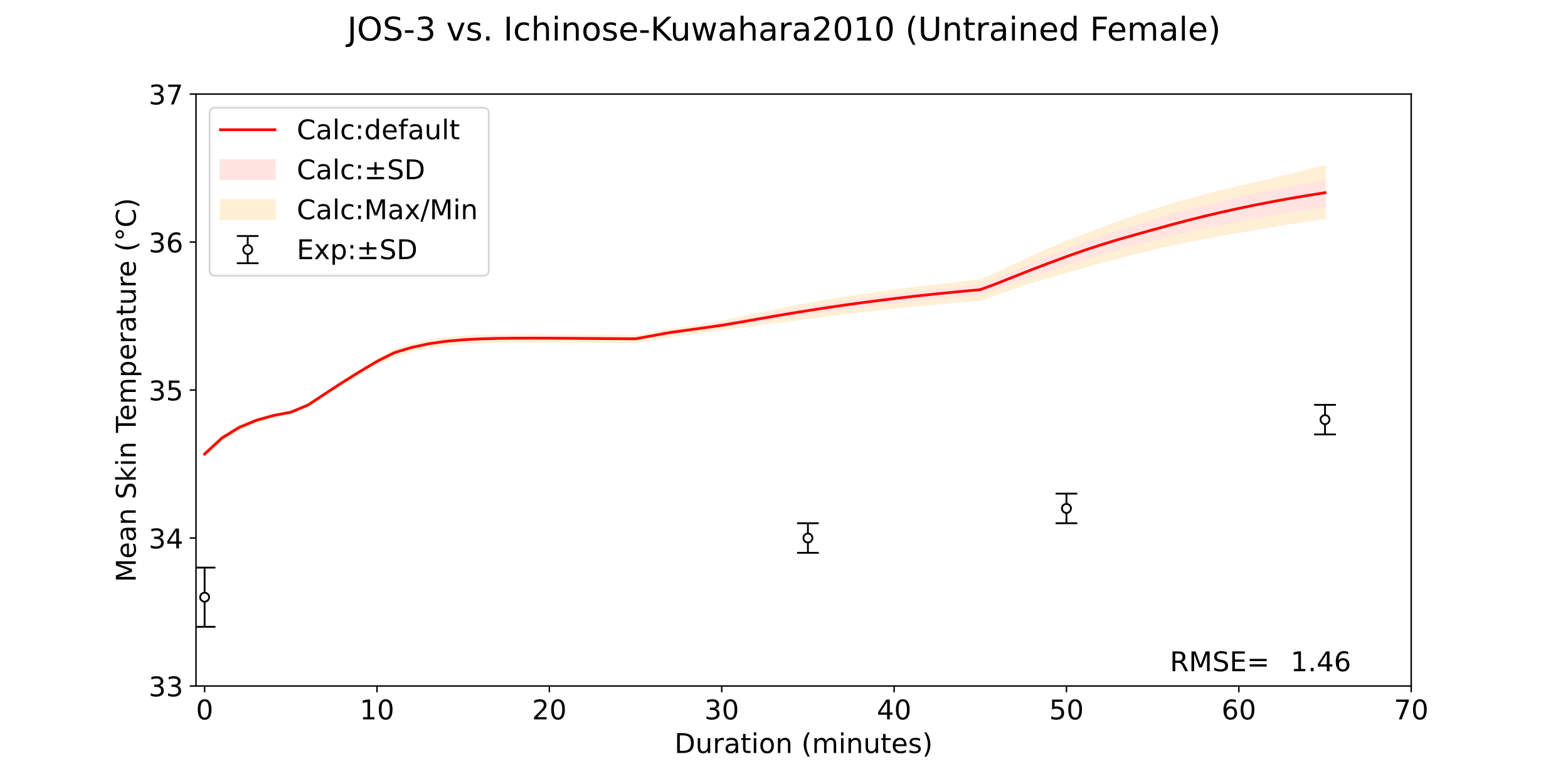


Supplementary Fig. 30 Mean skin temperature reproduced by the joint system thermoregulation model [JOS-3] (case 1: common laboratory exercises, Ichinose-Kuwahara et al. (2010), untrained female, n=10); For the four parameters of height, weight, age, and metabolic rate, three patterns of mean values and mean ± standard deviation were set and exhaustively combined, resulting in 81 calculation patterns.


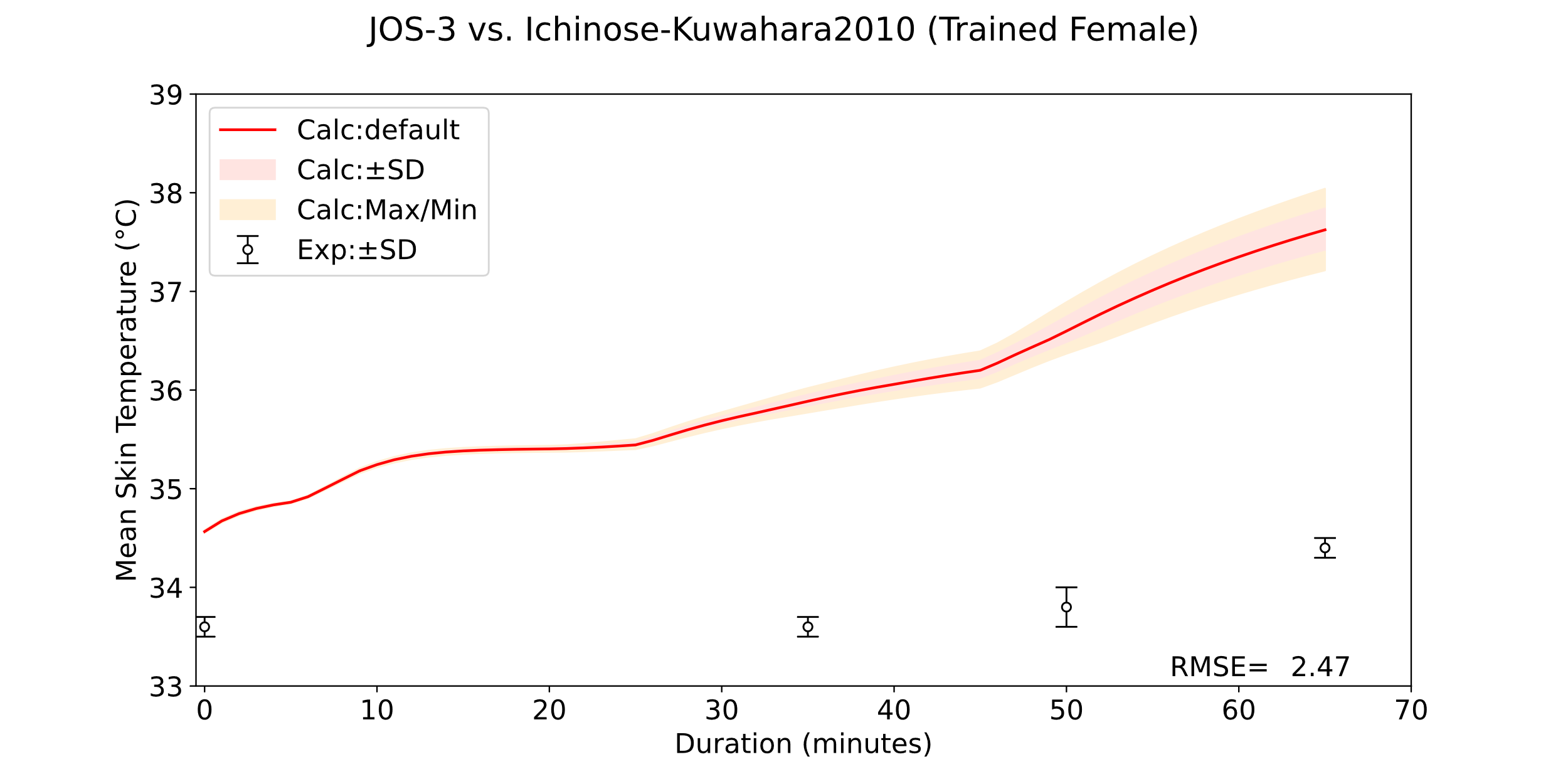


Supplementary Fig. 31 Mean skin temperature reproduced by the joint system thermoregulation model [JOS-3] (case 2: common laboratory exercises, Ichinose-Kuwahara et al. (2010), trained female, n=10); For the four parameters of height, weight, age, and metabolic rate, three patterns of mean values and mean ± standard deviation were set and exhaustively combined, resulting in 81 calculation patterns.


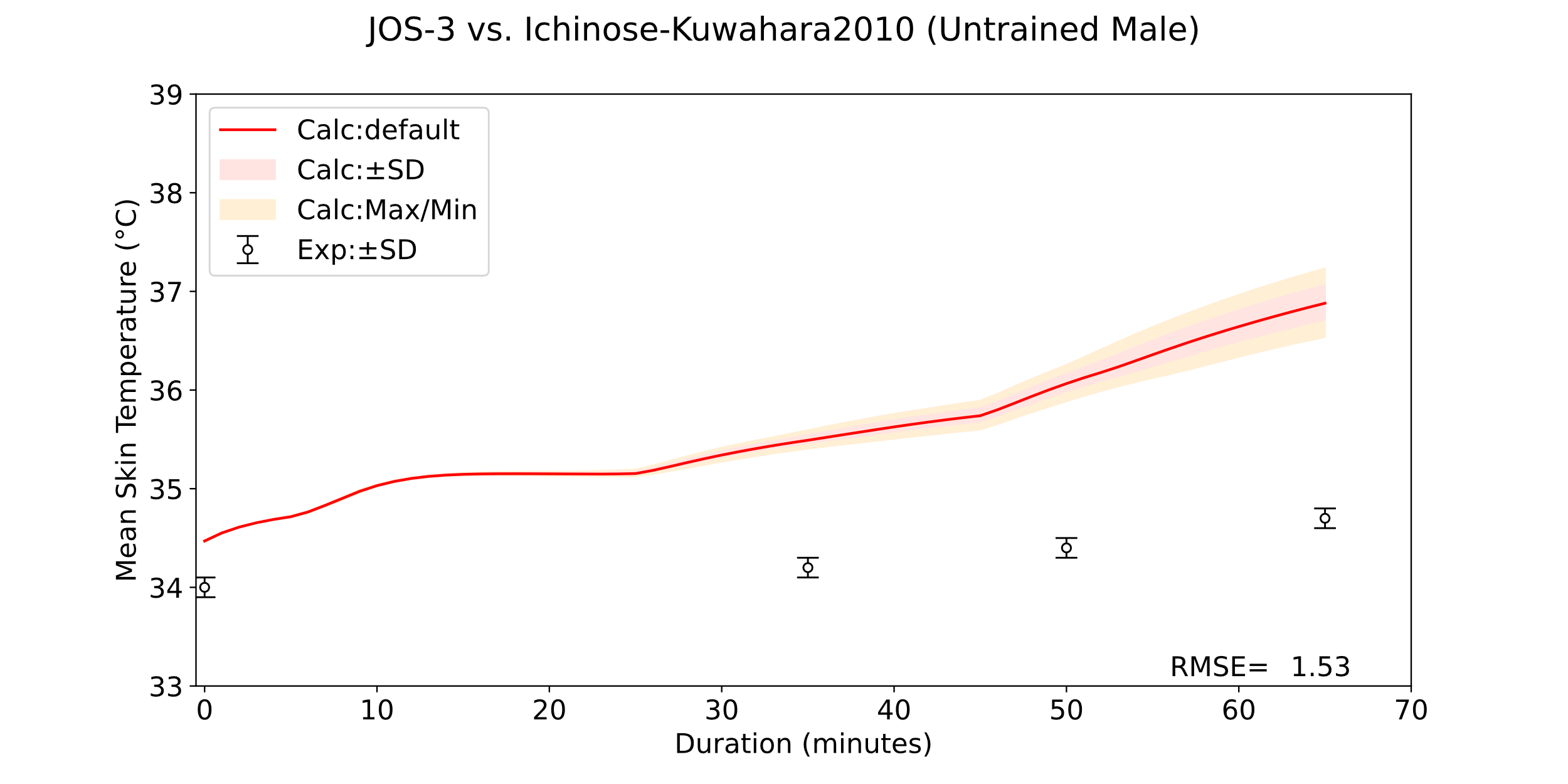


Supplementary Fig. 32 Mean skin temperature reproduced by the joint system thermoregulation model [JOS-3] (case 3: common laboratory exercises, Ichinose-Kuwahara et al. (2010), untrained male, n=9); For the four parameters of height, weight, age, and metabolic rate, three patterns of mean values and mean ± standard deviation were set and exhaustively combined, resulting in 81 calculation patterns.


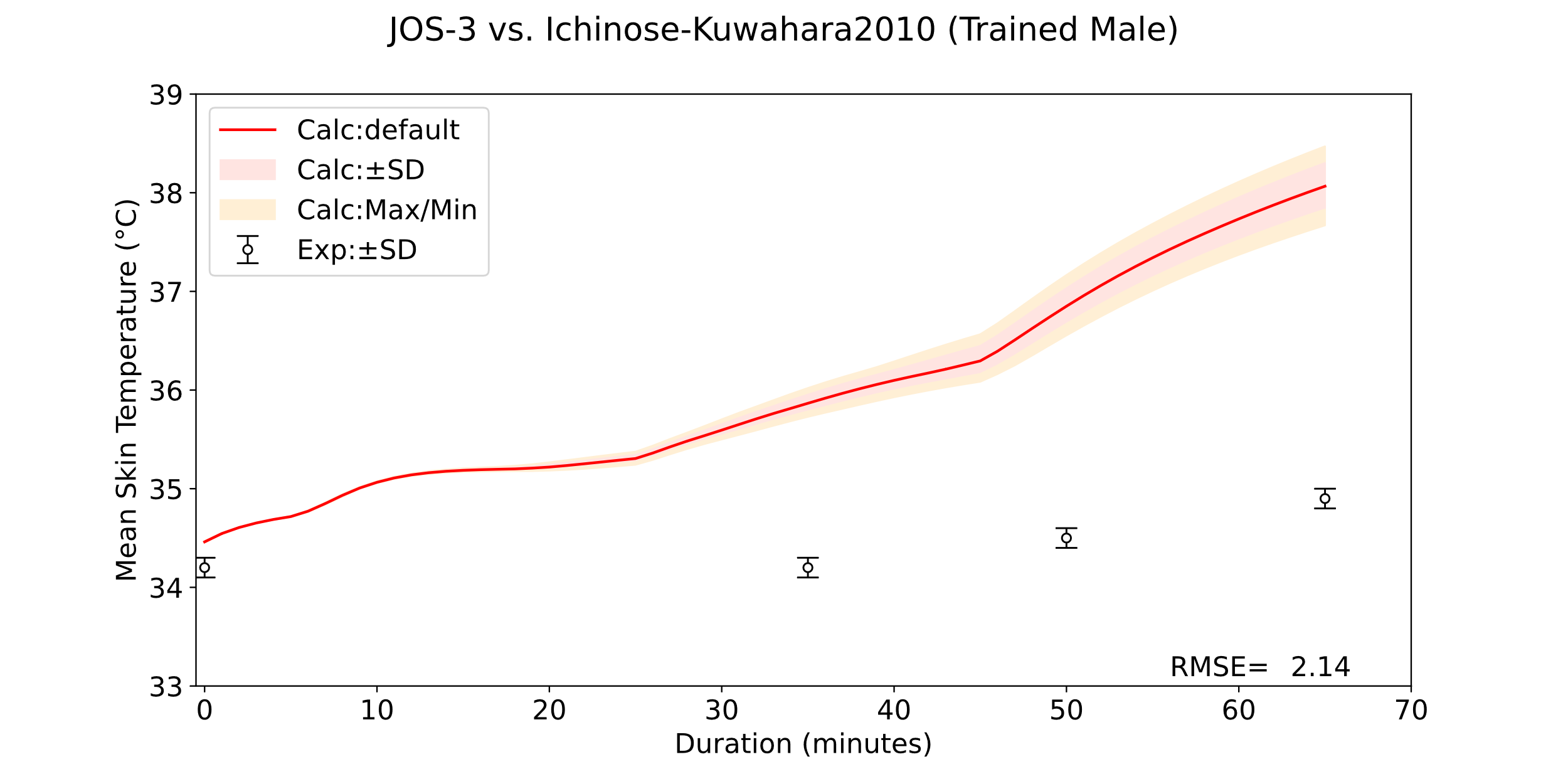


Supplementary Fig. 33 Mean skin temperature reproduced by the joint system thermoregulation model [JOS-3] (case 4: common laboratory exercises, Ichinose-Kuwahara et al. (2010), trained male, n=8); For the four parameters of height, weight, age, and metabolic rate, three patterns of mean values and mean ± standard deviation were set and exhaustively combined, resulting in 81 calculation patterns.


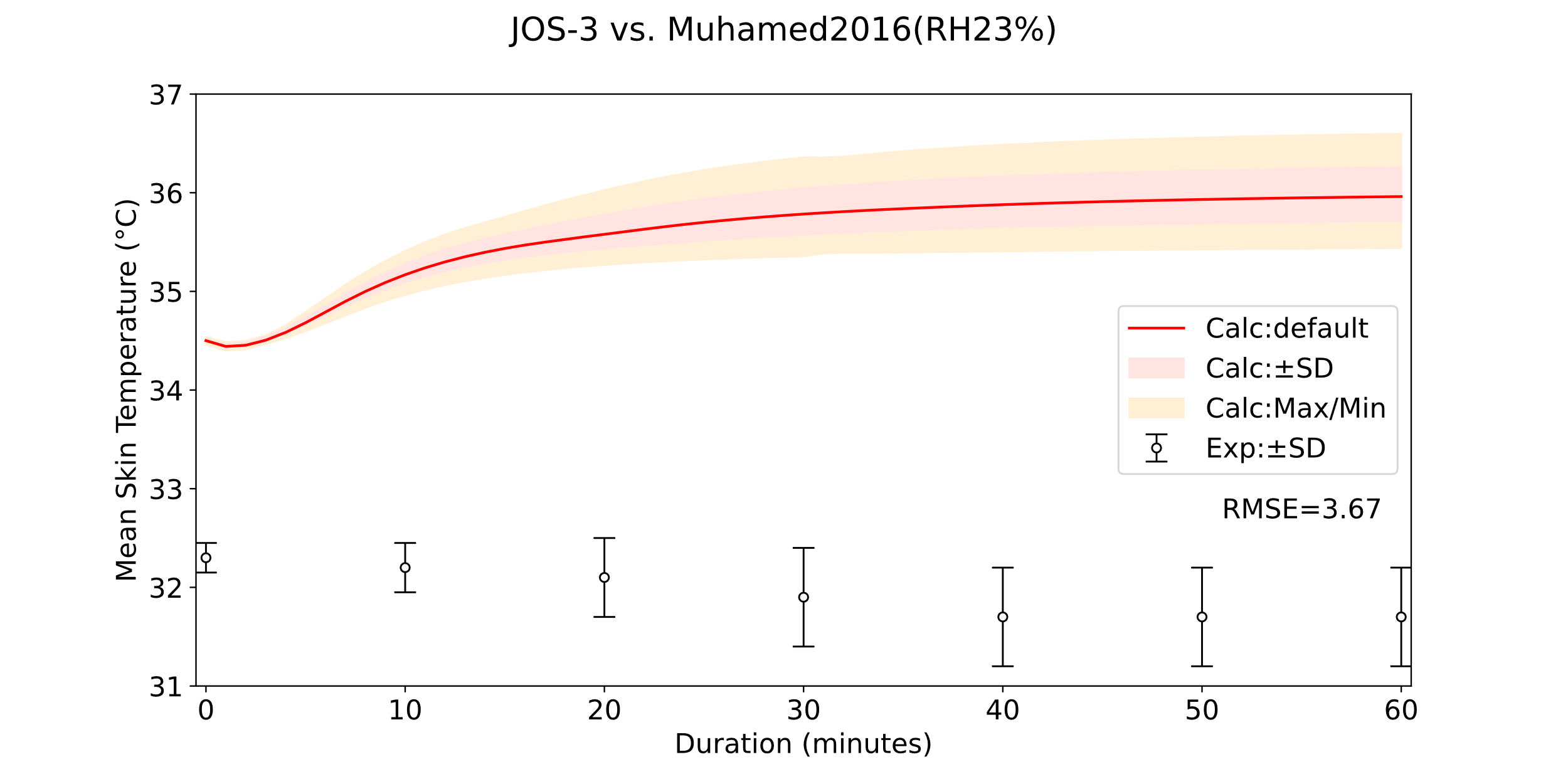


Supplementary Fig. 34 Mean skin temperature reproduced by the joint system thermoregulation model [JOS-3] (case 5: common laboratory exercises, Muhamed et al. (2016), RH=23%, n=12); Three patterns of mean and mean ± standard deviation were set for the four parameters of height, weight, age, and metabolic rate, and three patterns for temperature trends, which were exhaustively combined, resulting in 243 calculation patterns.


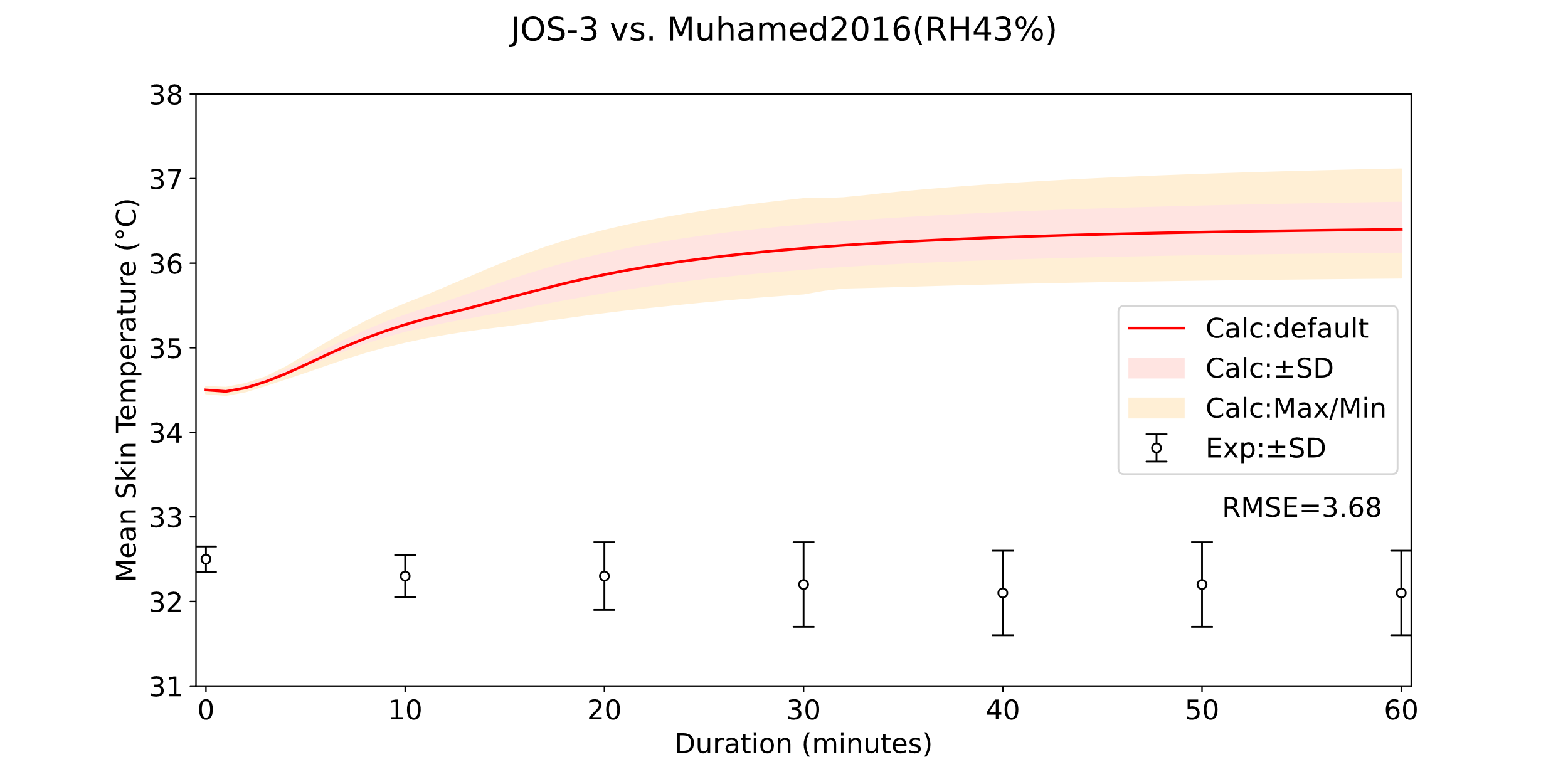


Supplementary Fig. 35 Mean skin temperature reproduced by the joint system thermoregulation model [JOS-3] (case 6: common laboratory exercises, Muhamed et al. (2016), RH=43%, n=12); Three patterns of mean and mean ± standard deviation were set for the four parameters of height, weight, age, and metabolic rate, and three patterns for temperature trends, which were exhaustively combined, resulting in 243 calculation patterns.


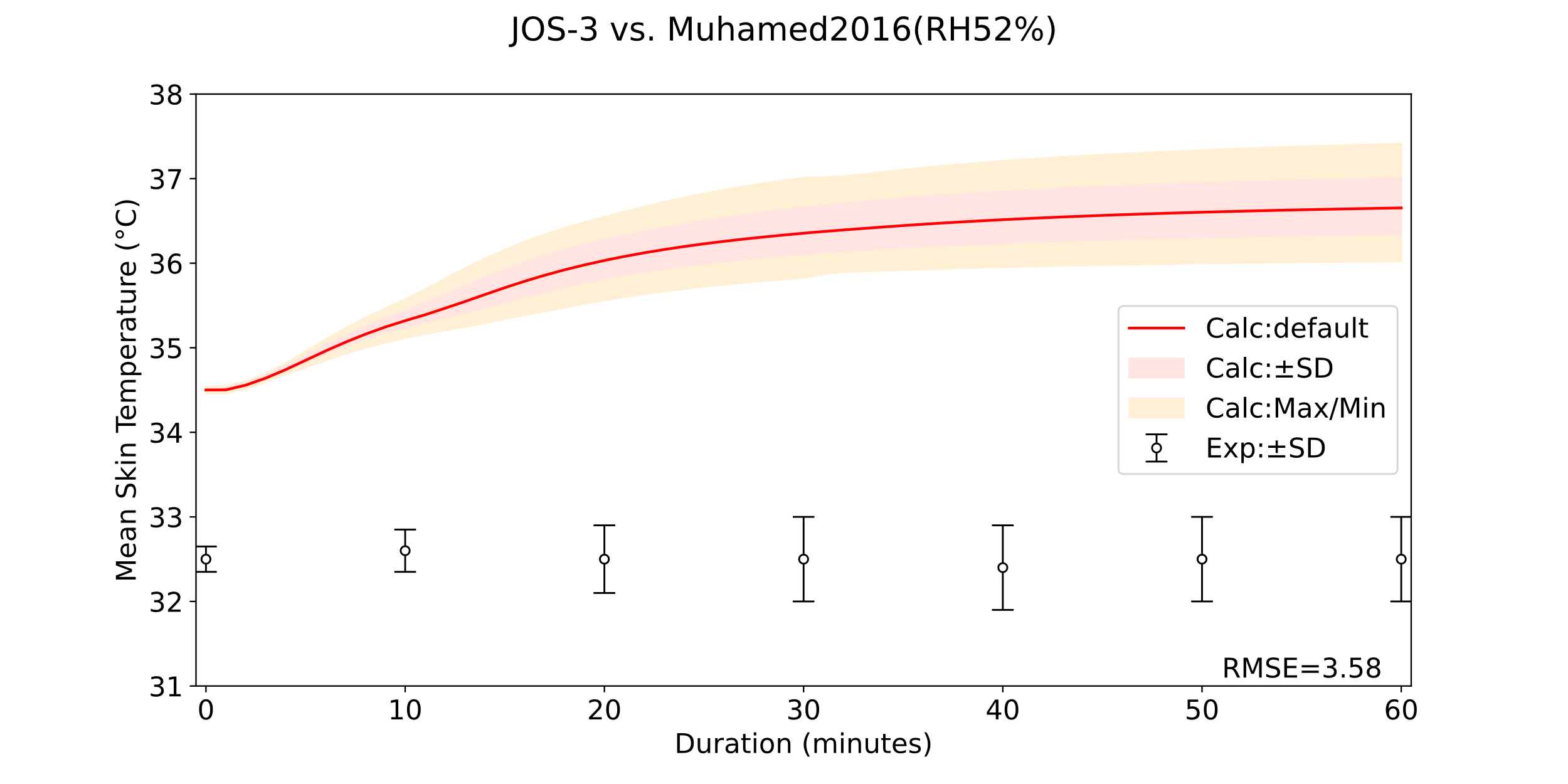


Supplementary Fig. 36 Mean skin temperature reproduced by the joint system thermoregulation model [JOS-3] (case 7: common laboratory exercises, Muhamed et al. (2016), RH=52%, n=12); Three patterns of mean and mean ± standard deviation were set for the four parameters of height, weight, age, and metabolic rate, and three patterns for temperature trends, which were exhaustively combined, resulting in 243 calculation patterns.


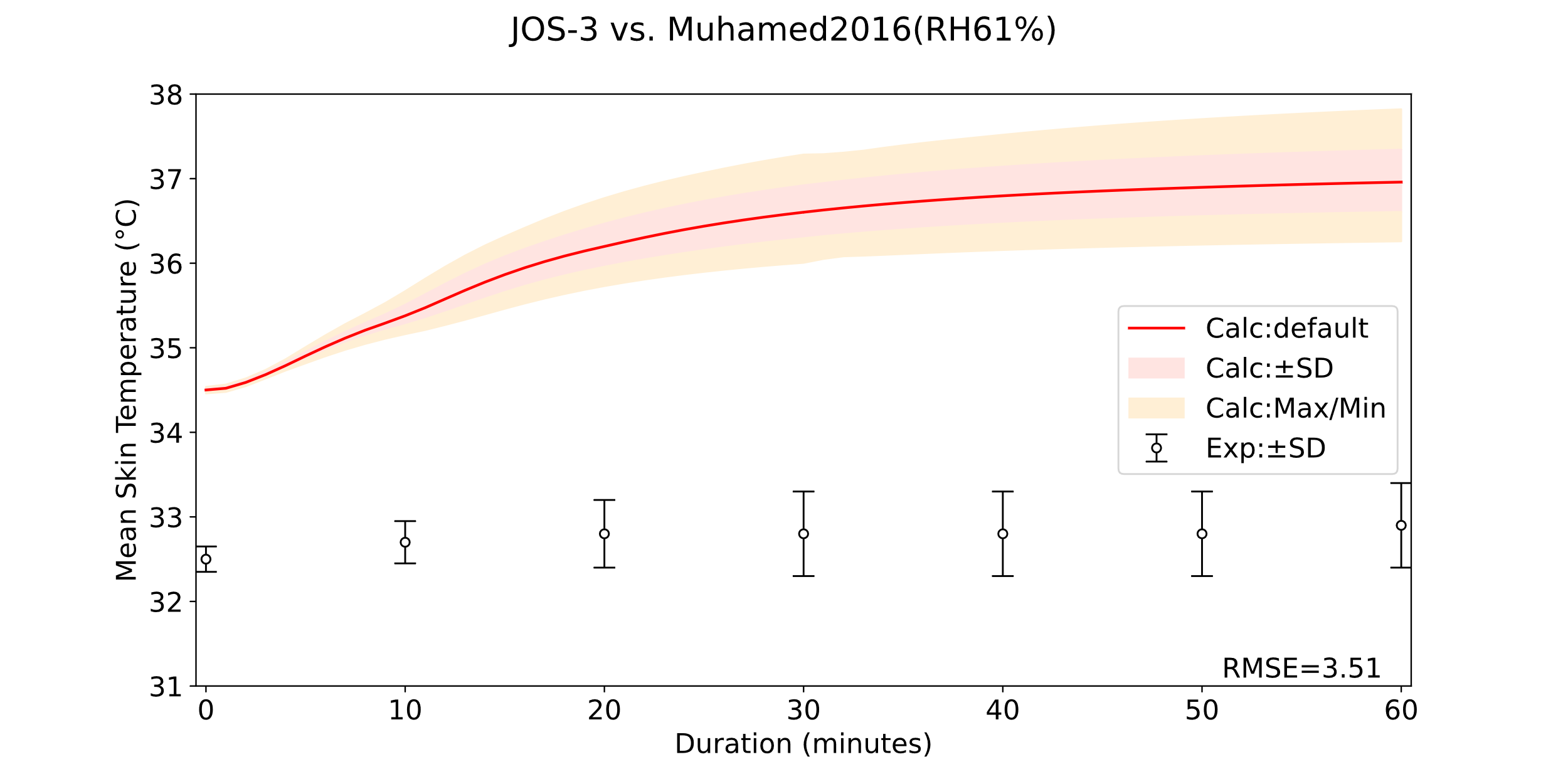


Supplementary Fig. 37 Mean skin temperature reproduced by the joint system thermoregulation model [JOS-3] (case 8: common laboratory exercises, Muhamed et al. (2016), RH=61%, n=12); Three patterns of mean and mean ± standard deviation were set for the four parameters of height, weight, age, and metabolic rate, and three patterns for temperature trends, which were exhaustively combined, resulting in 243 calculation patterns.


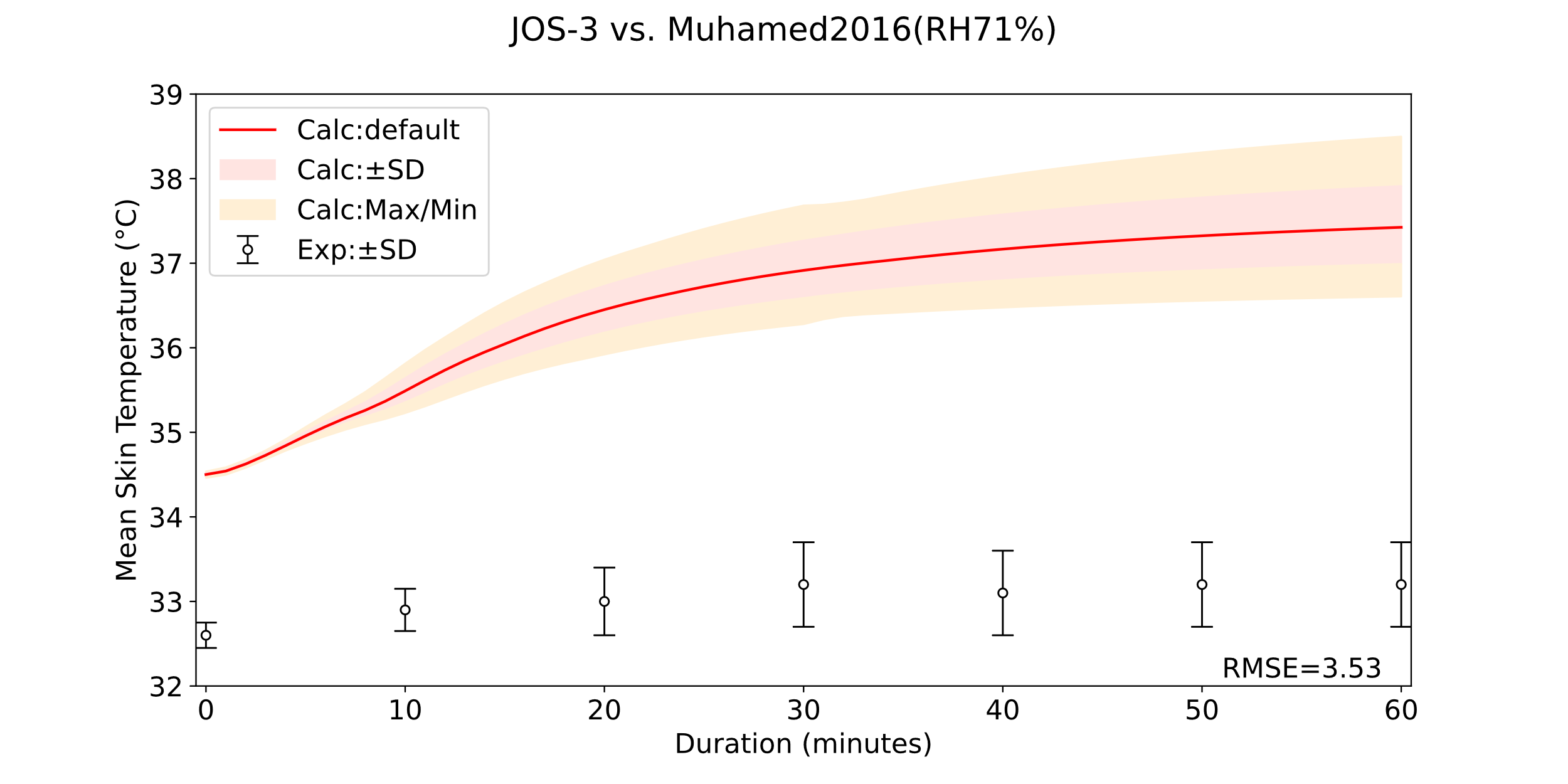


Supplementary Fig. 38 Mean skin temperature reproduced by the joint system thermoregulation model [JOS-3] (case 9: common laboratory exercises, Muhamed et al. (2016), RH=71%, n=12); Three patterns of mean and mean ± standard deviation were set for the four parameters of height, weight, age, and metabolic rate, and three patterns for temperature trends, which were exhaustively combined, resulting in 243 calculation patterns.


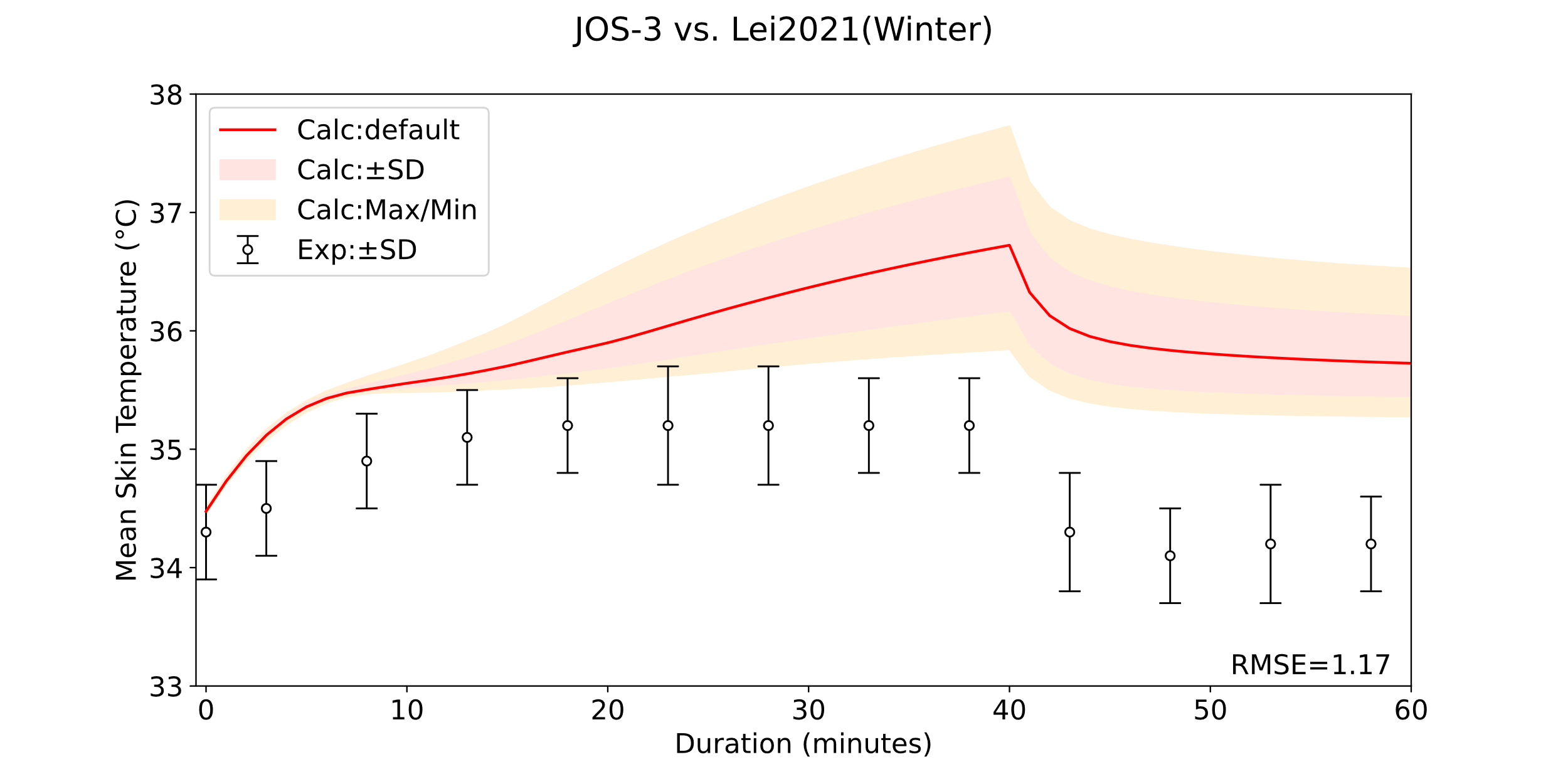


Supplementary Fig. 39 Mean skin temperature reproduced by the joint system thermoregulation model [JOS-3] (case 10: common laboratory exercises, Lei et al. (2021), winter, n=12); For the four parameters of height, weight, age, and metabolic rate, three patterns of mean values and mean ± standard deviation were set and exhaustively combined, resulting in 81 calculation patterns.


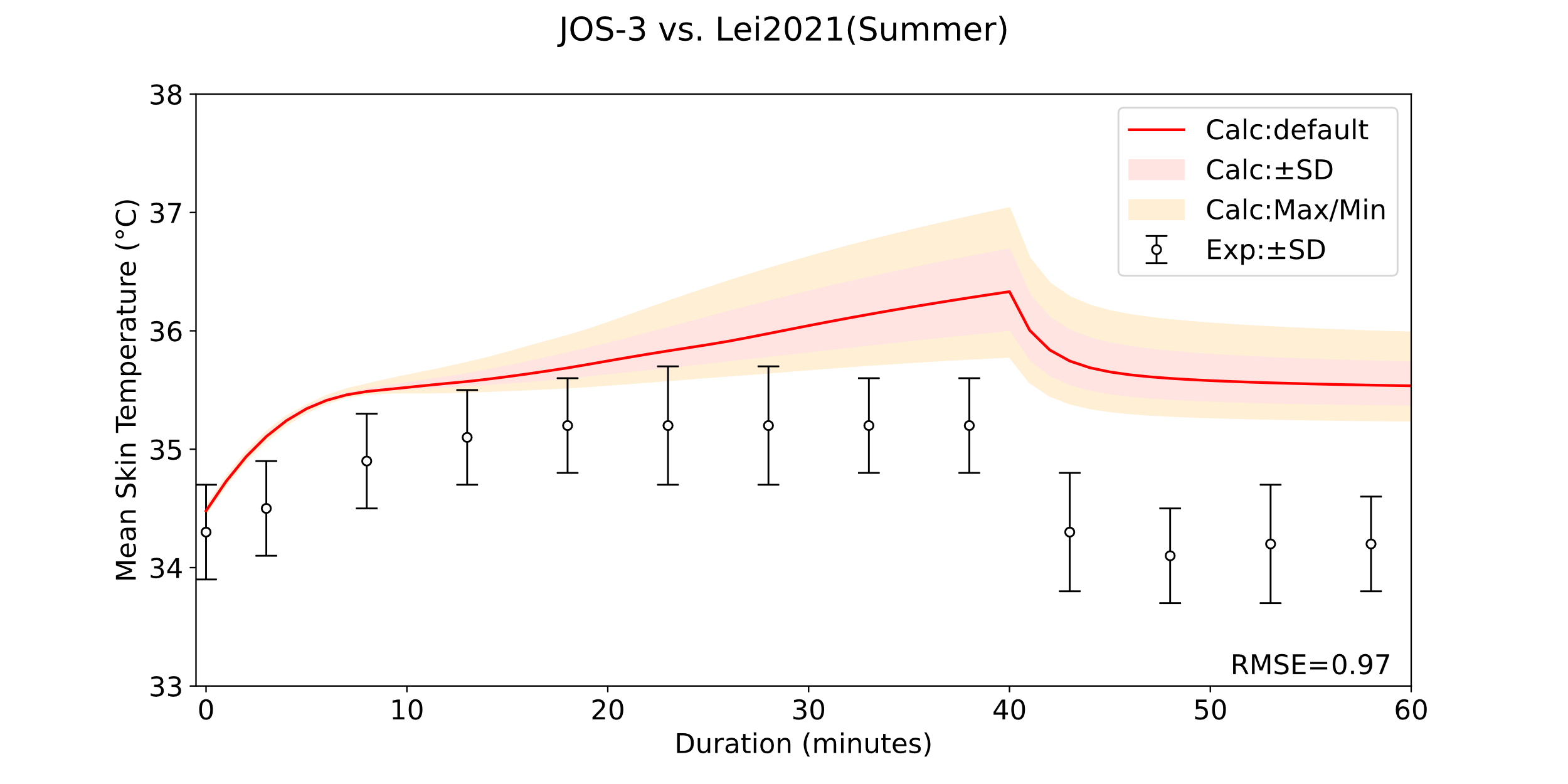


Supplementary Fig. 40 Mean skin temperature reproduced by the joint system thermoregulation model [JOS-3] (case 11: common laboratory exercises, Lei et al. (2021), summer, n=12); For the four parameters of height, weight, age, and metabolic rate, three patterns of mean values and mean ± standard deviation were set and exhaustively combined, resulting in 81 calculation patterns.


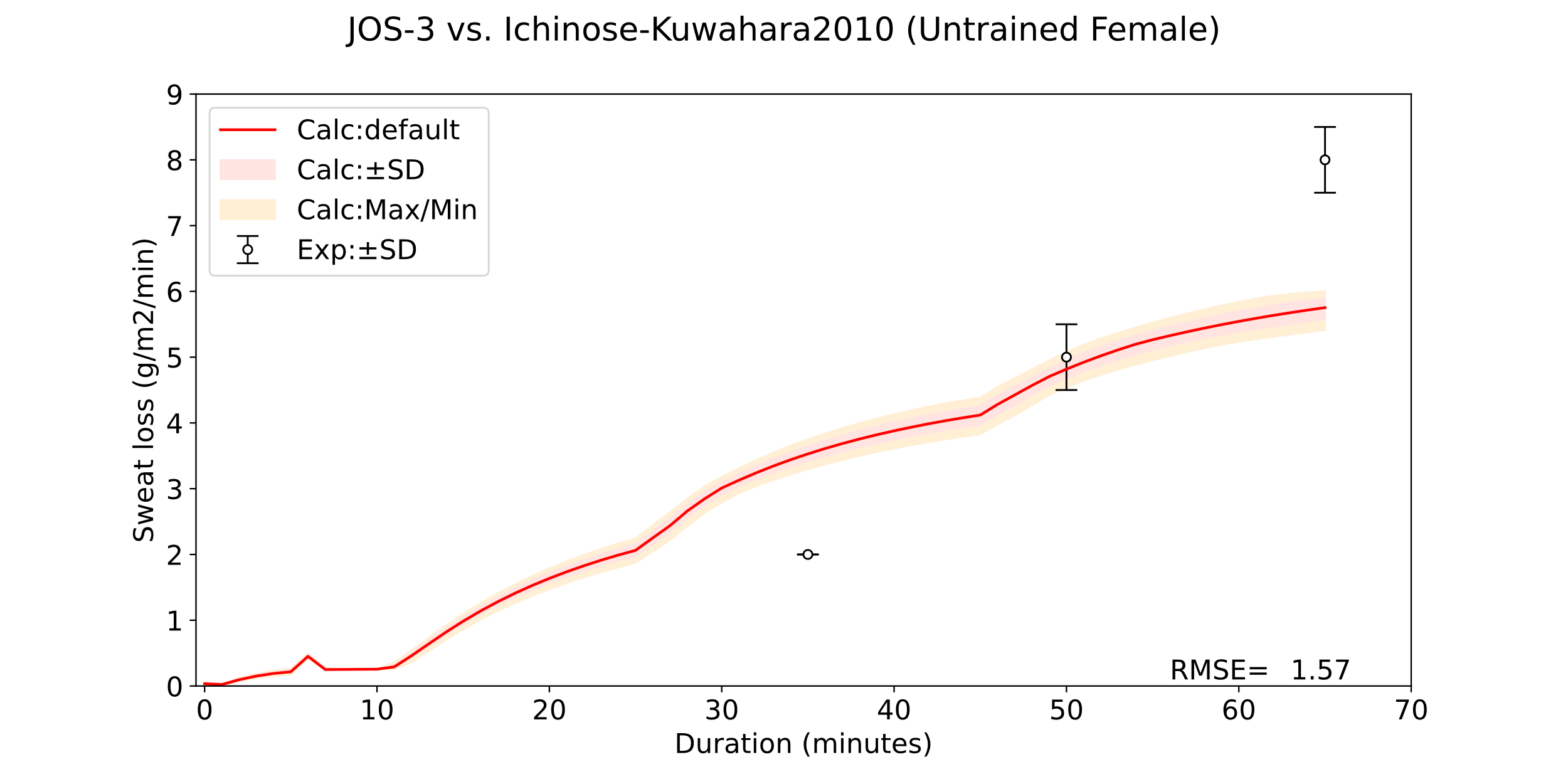


Supplementary Fig. 41 Sweat loss reproduced by the joint system thermoregulation model [JOS-3] (case 1: common laboratory exercises, Ichinose-Kuwahara et al. (2010), untrained female, n=10); For the four parameters of height, weight, age, and metabolic rate, three patterns of mean values and mean ± standard deviation were set and exhaustively combined, resulting in 81 calculation patterns.


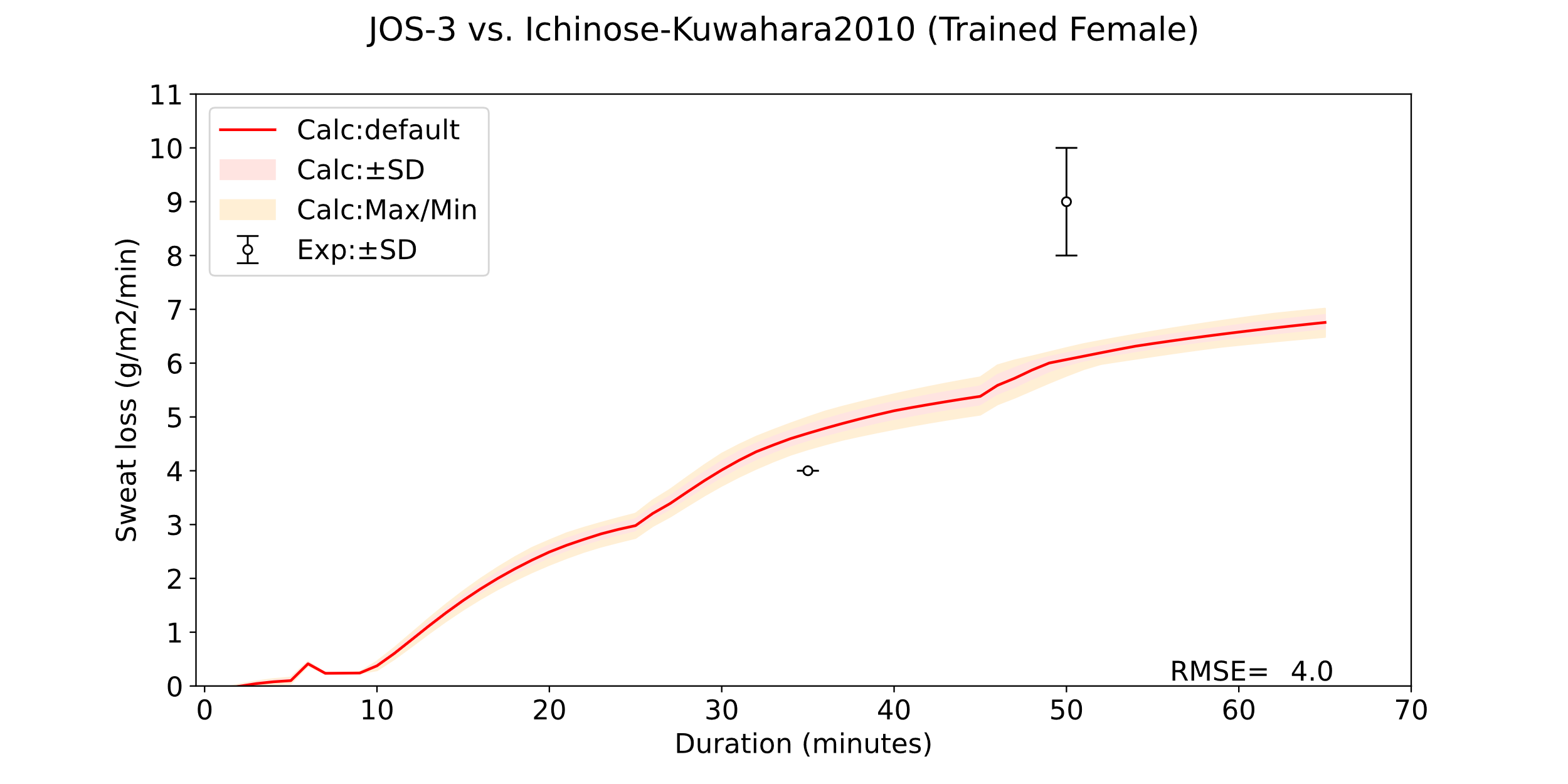


Supplementary Fig. 42 Sweat loss reproduced by the joint system thermoregulation model [JOS-3] (case 2: common laboratory exercises, Ichinose-Kuwahara et al. (2010), trained female, n=10); For the four parameters of height, weight, age, and metabolic rate, three patterns of mean values and mean ± standard deviation were set and exhaustively combined, resulting in 81 calculation patterns.


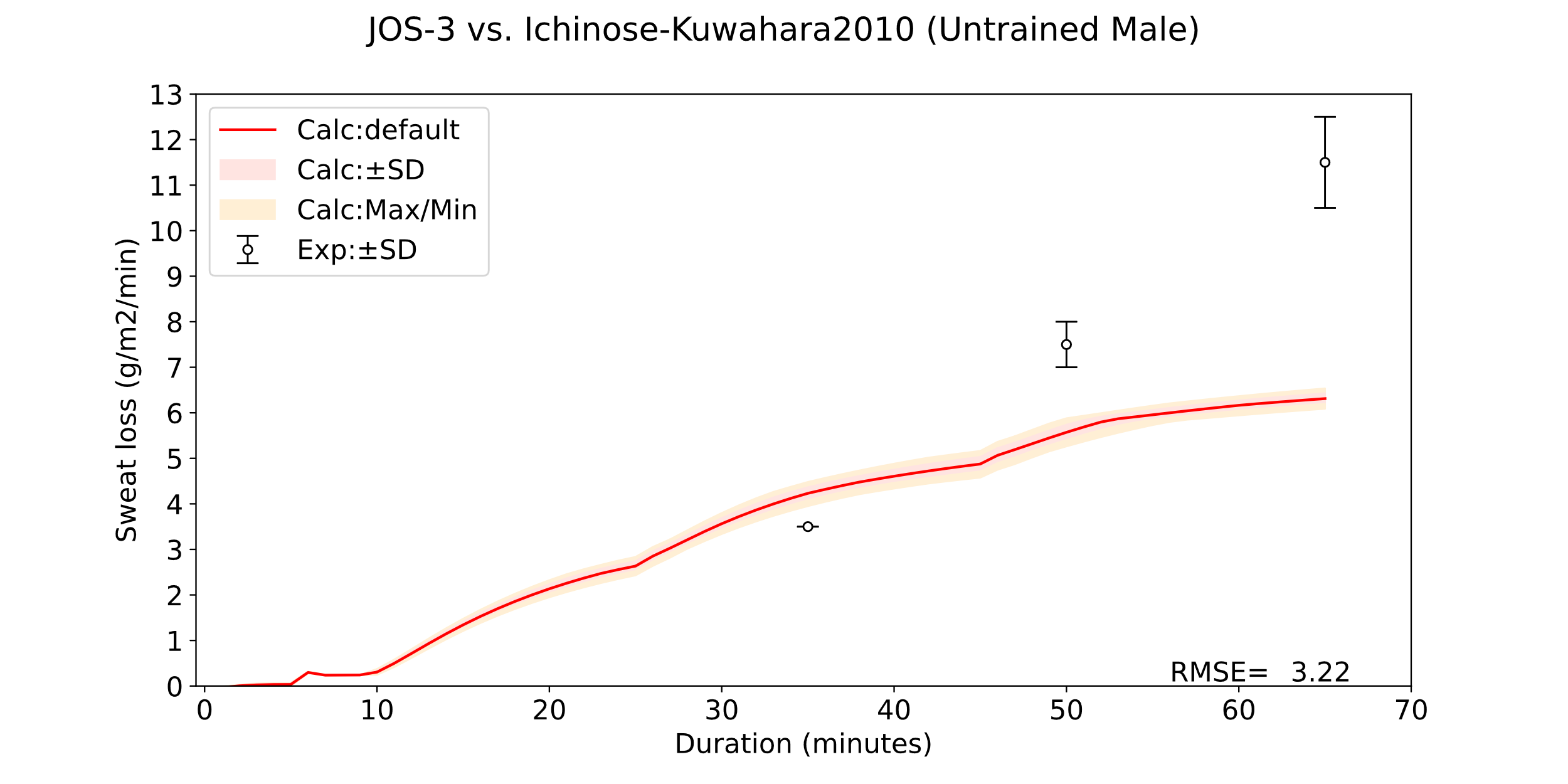


Supplementary Fig. 43 Sweat loss reproduced by the joint system thermoregulation model [JOS-3] (case 3: common laboratory exercises, Ichinose-Kuwahara et al. (2010), untrained male, n=9); For the four parameters of height, weight, age, and metabolic rate, three patterns of mean values and mean ± standard deviation were set and exhaustively combined, resulting in 81 calculation patterns.


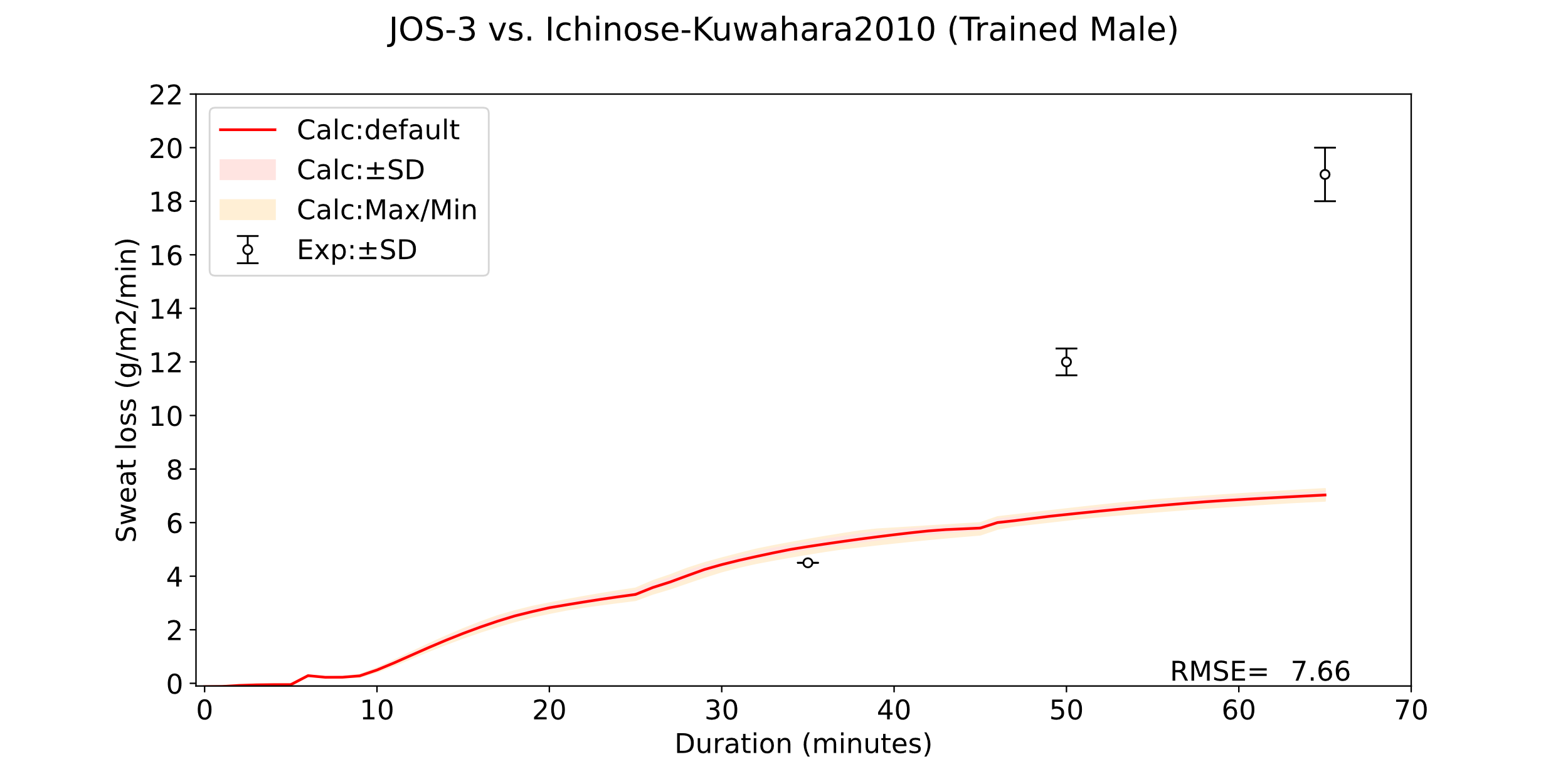


Supplementary Fig. 44 Sweat loss reproduced by the joint system thermoregulation model [JOS-3] (case 4: common laboratory exercises, Ichinose-Kuwahara et al. (2010), trained male, n=8); For the four parameters of height, weight, age, and metabolic rate, three patterns of mean values and mean ± standard deviation were set and exhaustively combined, resulting in 81 calculation patterns.


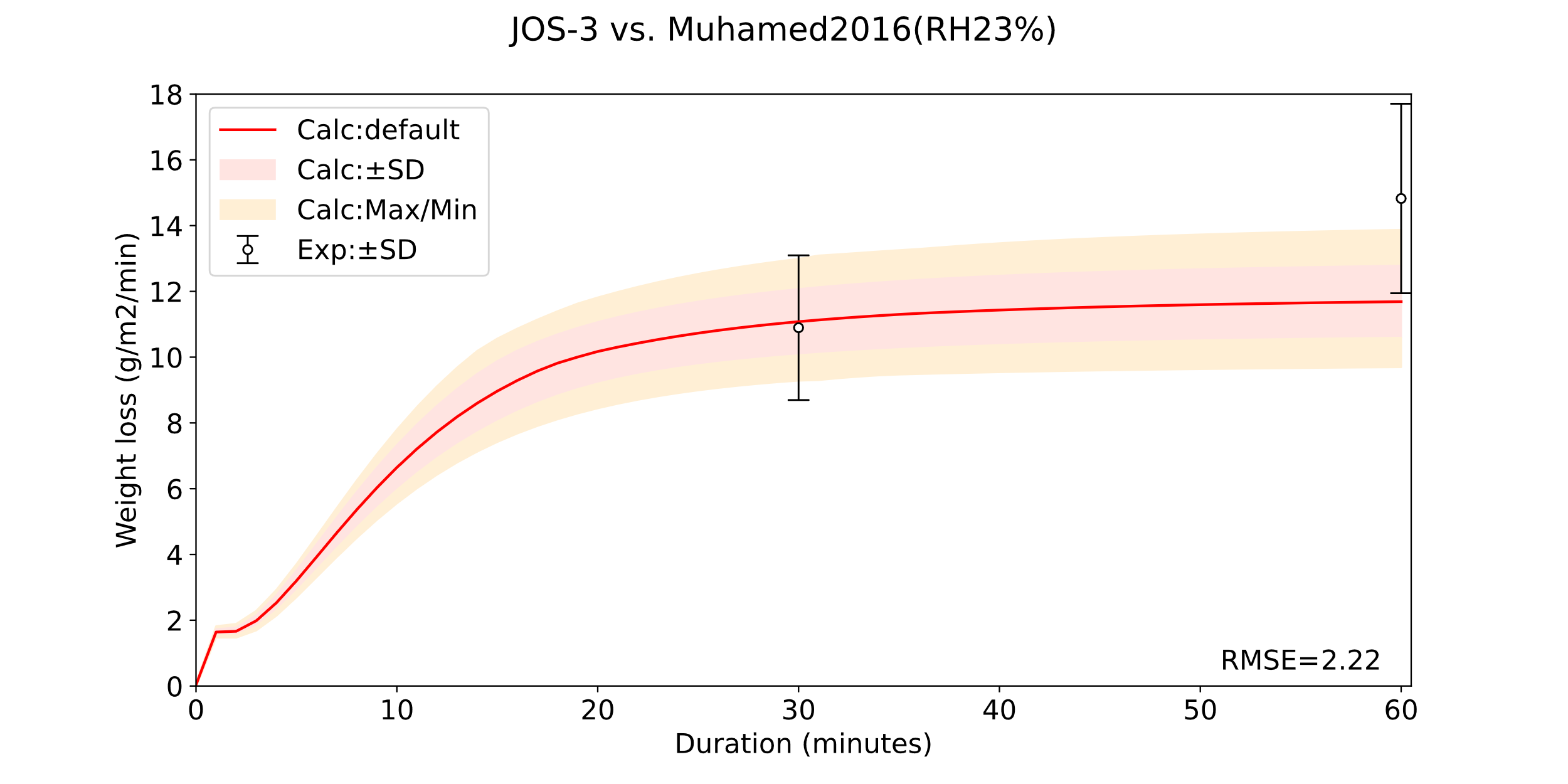


Supplementary Fig. 45 Sweat loss reproduced by the joint system thermoregulation model [JOS-3] (case 5: common laboratory exercises, Muhamed et al. (2016), RH=23%, n=12); Three patterns of mean and mean ± standard deviation were set for the four parameters of height, weight, age, and metabolic rate, and three patterns for temperature trends, which were exhaustively combined, resulting in 243 calculation patterns.


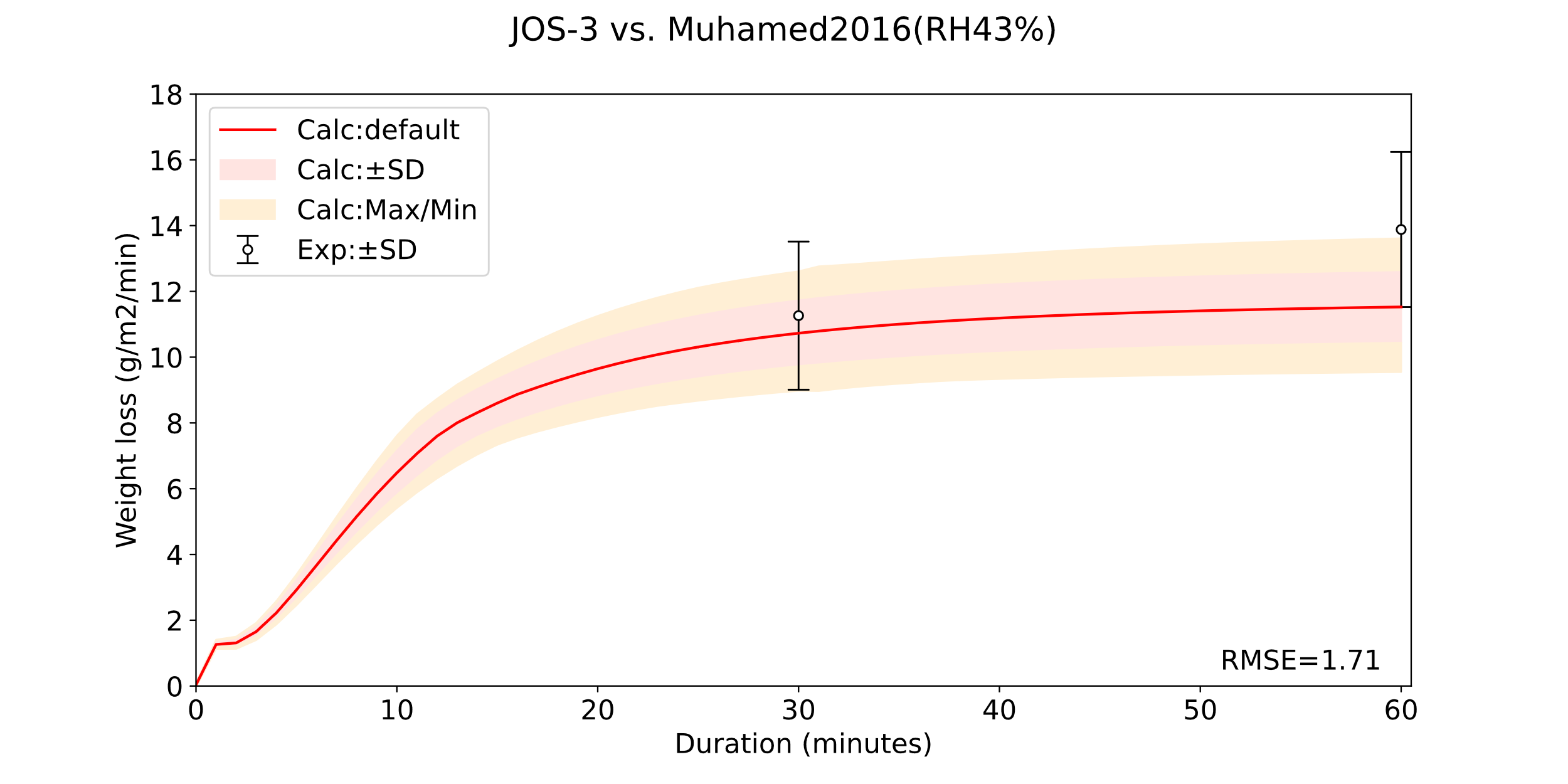


Supplementary Fig. 46 Sweat loss reproduced by the joint system thermoregulation model [JOS-3] (case 6: common laboratory exercises, Muhamed et al. (2016), RH=43%, n=12) ; Three patterns of mean and mean ± standard deviation were set for the four parameters of height, weight, age, and metabolic rate, and three patterns for temperature trends, which were exhaustively combined, resulting in 243 calculation patterns.


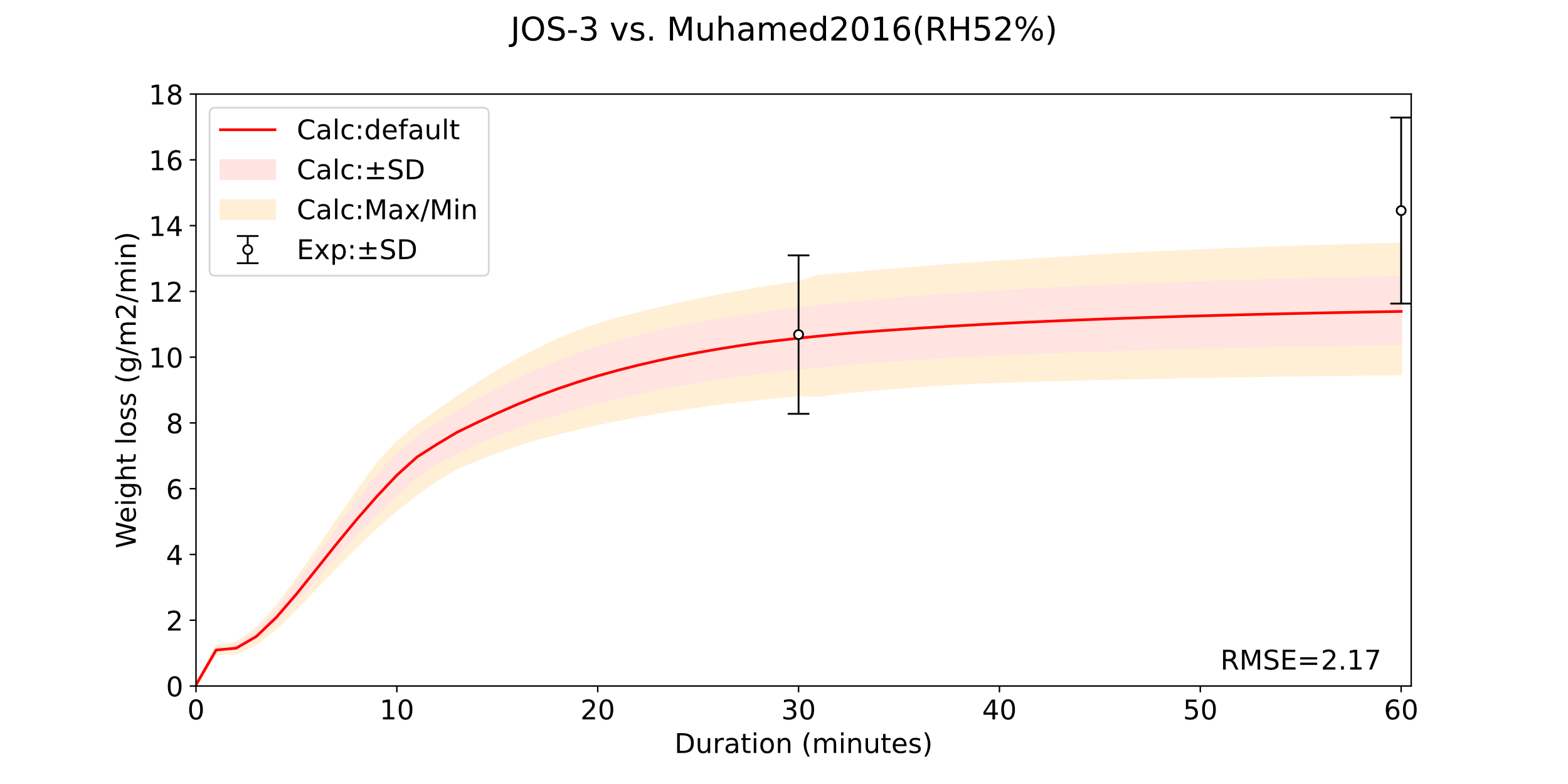


Supplementary Fig. 47 Sweat loss reproduced by the joint system thermoregulation model [JOS-3] (case 7: common laboratory exercises, Muhamed et al. (2016), RH=52%, n=12); Three patterns of mean and mean ± standard deviation were set for the four parameters of height, weight, age, and metabolic rate, and three patterns for temperature trends, which were exhaustively combined, resulting in 243 calculation patterns.


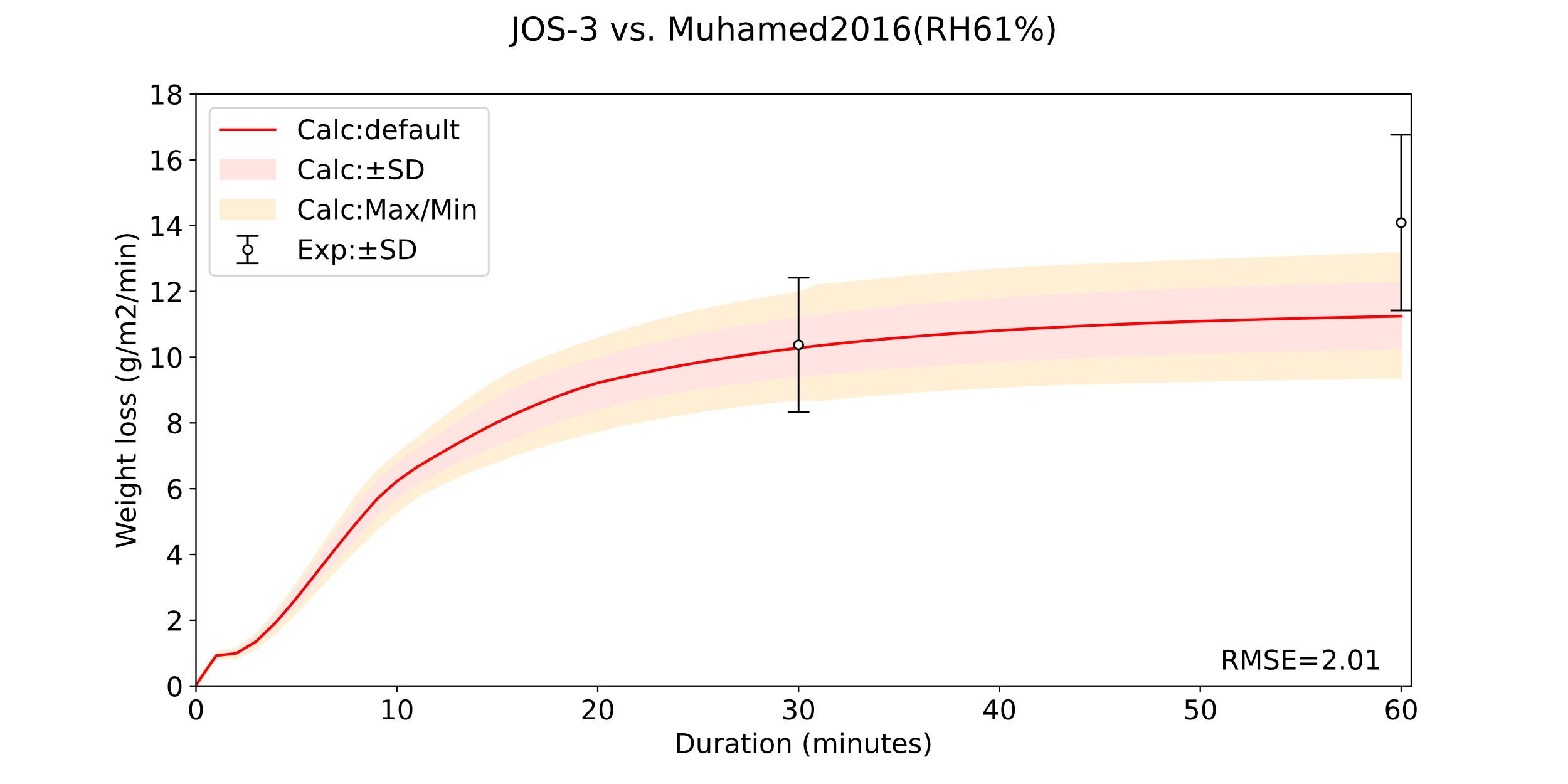


Supplementary Fig. 48 Sweat loss reproduced by the joint system thermoregulation model [JOS-3] (case 8: common laboratory exercises, Muhamed et al. (2016), RH=61%, n=12); Three patterns of mean and mean ± standard deviation were set for the four parameters of height, weight, age, and metabolic rate, and three patterns for temperature trends, which were exhaustively combined, resulting in 243 calculation patterns.


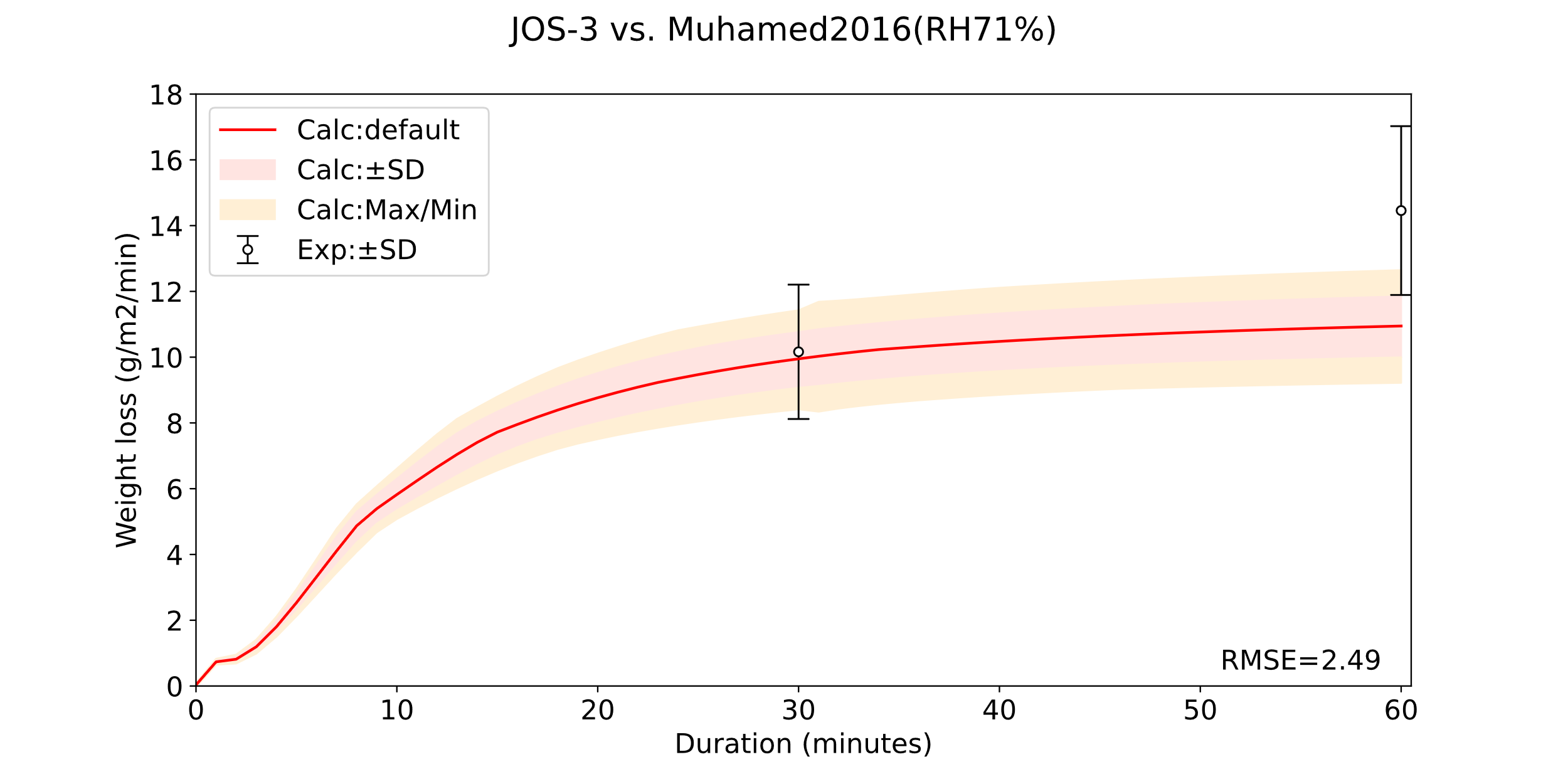


Supplementary Fig. 49 Sweat loss reproduced by the joint system thermoregulation model [JOS-3] (case 9: common laboratory exercises, Muhamed et al. (2016), RH=71%, n=12); Three patterns of mean and mean ± standard deviation were set for the four parameters of height, weight, age, and metabolic rate, and three patterns for temperature trends, which were exhaustively combined, resulting in 243 calculation patterns.


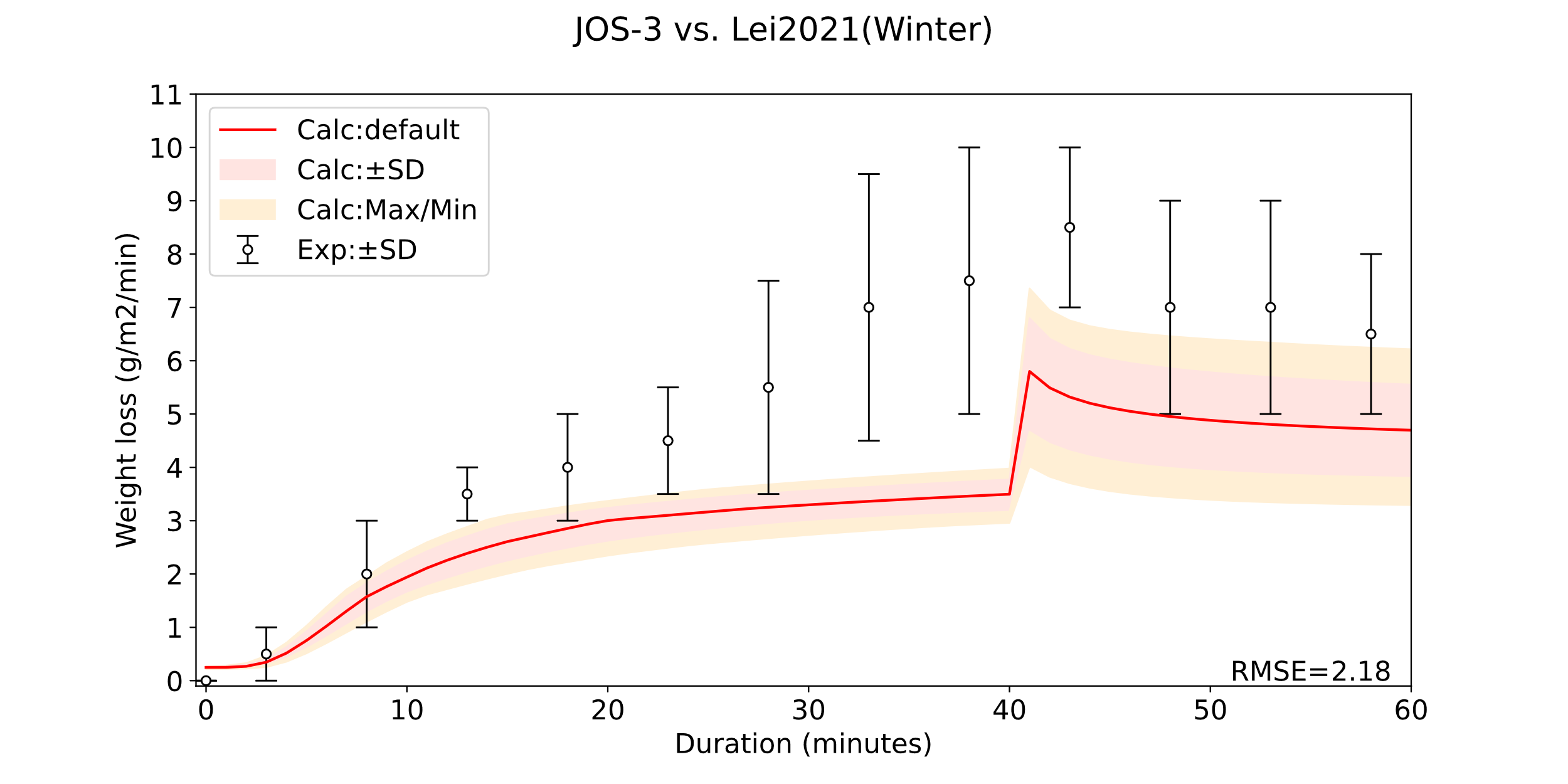


Supplementary Fig. 50 Sweat loss reproduced by the joint system thermoregulation model [JOS-3] (case 10: common laboratory exercises, Lei et al. (2021), winter, n=12); For the four parameters of height, weight, age, and metabolic rate, three patterns of mean values and mean ± standard deviation were set and exhaustively combined, resulting in 81 calculation patterns.


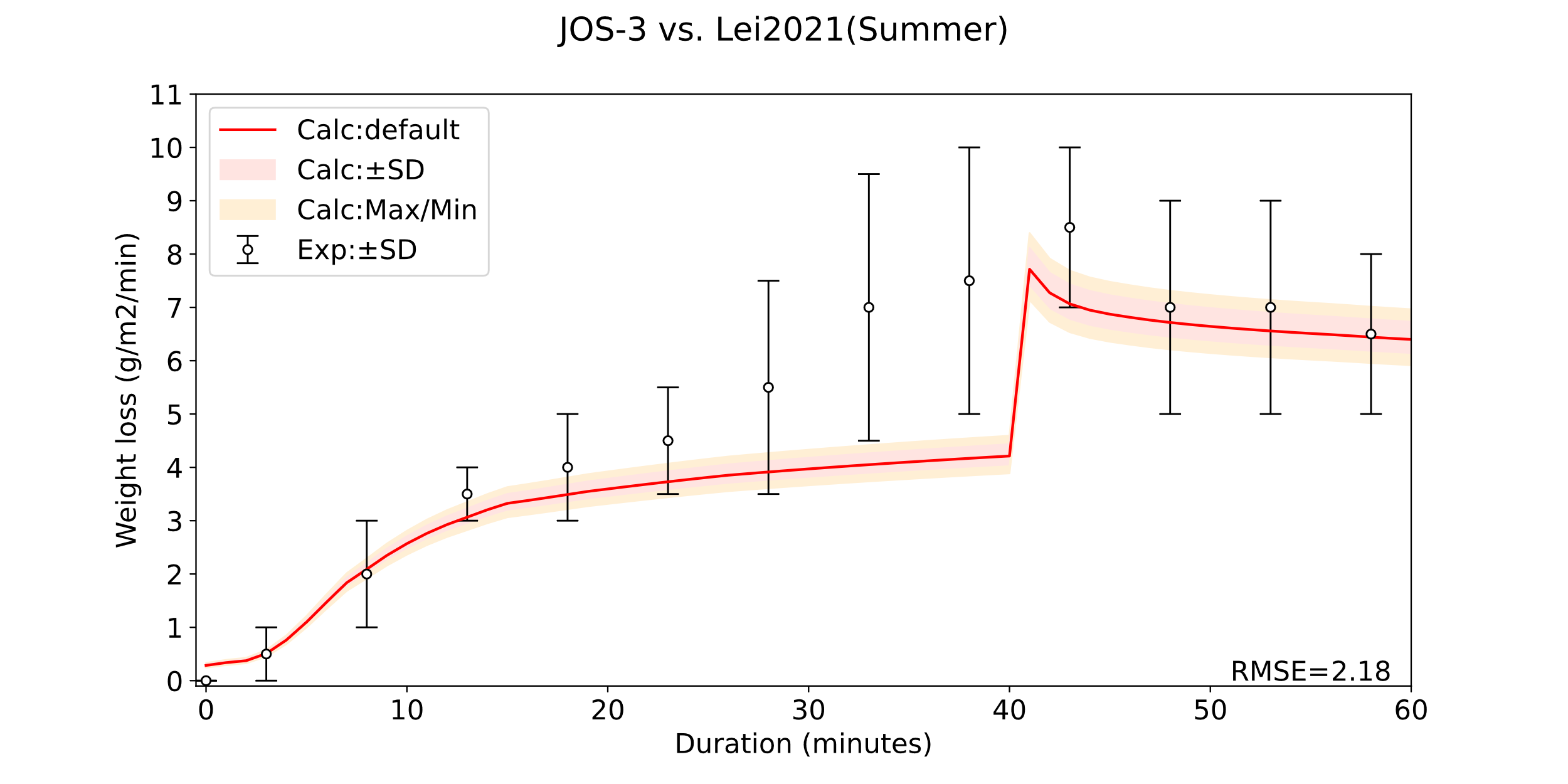


**Supplementary Fig. 51** Sweat loss reproduced by the joint system thermoregulation model [JOS-3] (case 11: common laboratory exercises, Lei et al. (2021), summer, n=12); For the four parameters of height, weight, age, and metabolic rate, three patterns of mean values and mean ± standard deviation were set and exhaustively combined, resulting in 81 calculation patterns.


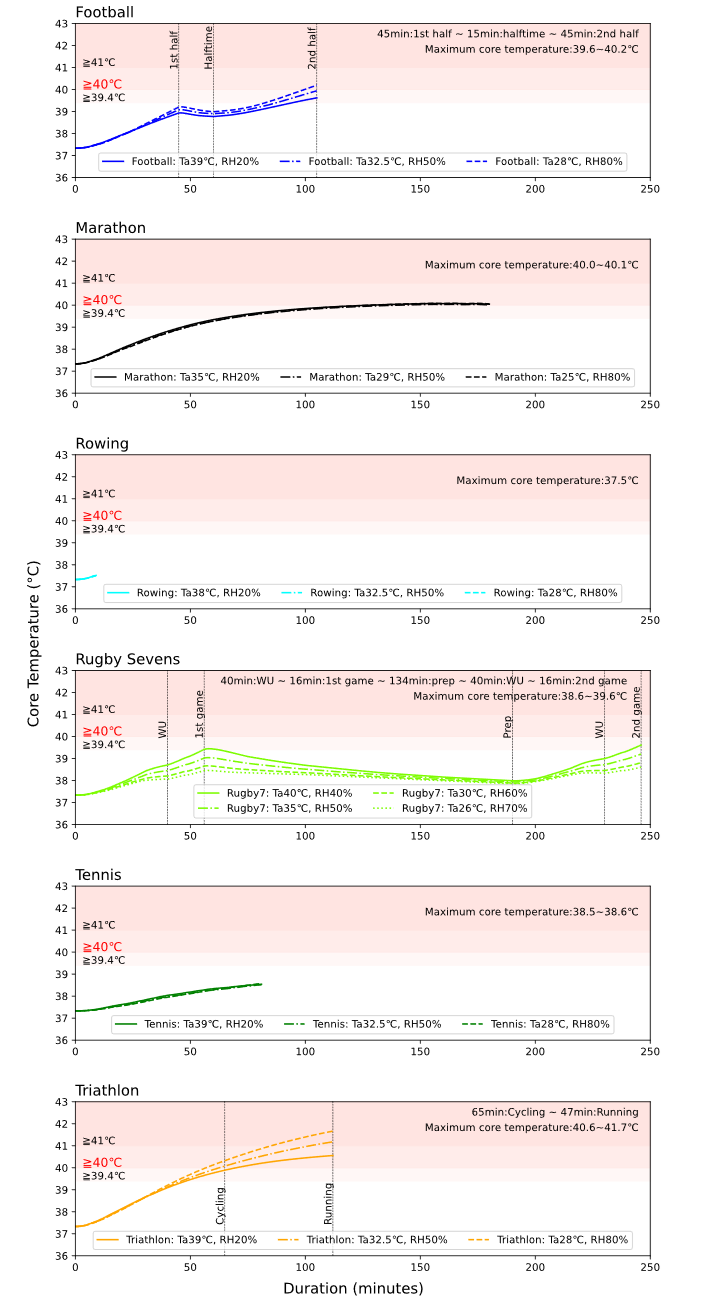


**Supplementary Fig. 52** Predicted core temperature in six sports at the upper thresholds of the thermal safety guidelines (90th percentile value)


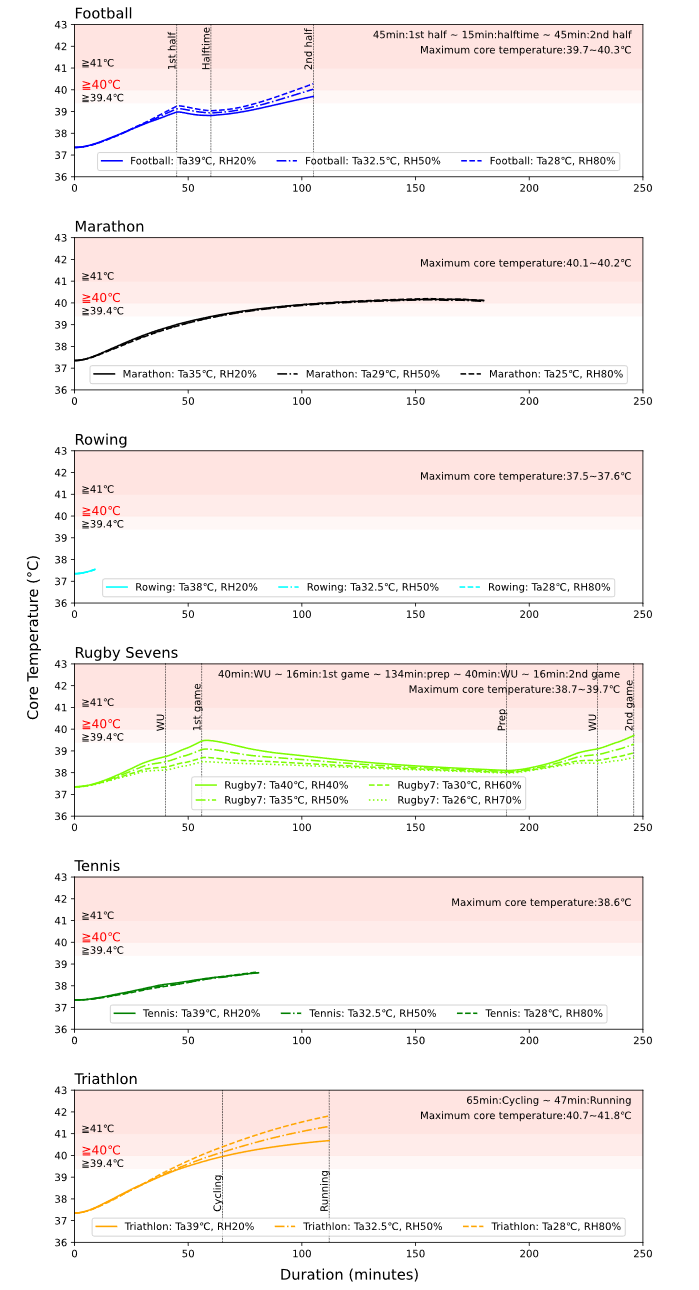


**Supplementary Fig. 53** Predicted core temperature in six sports at the upper thresholds of the thermal safety guidelines (95th percentile value)


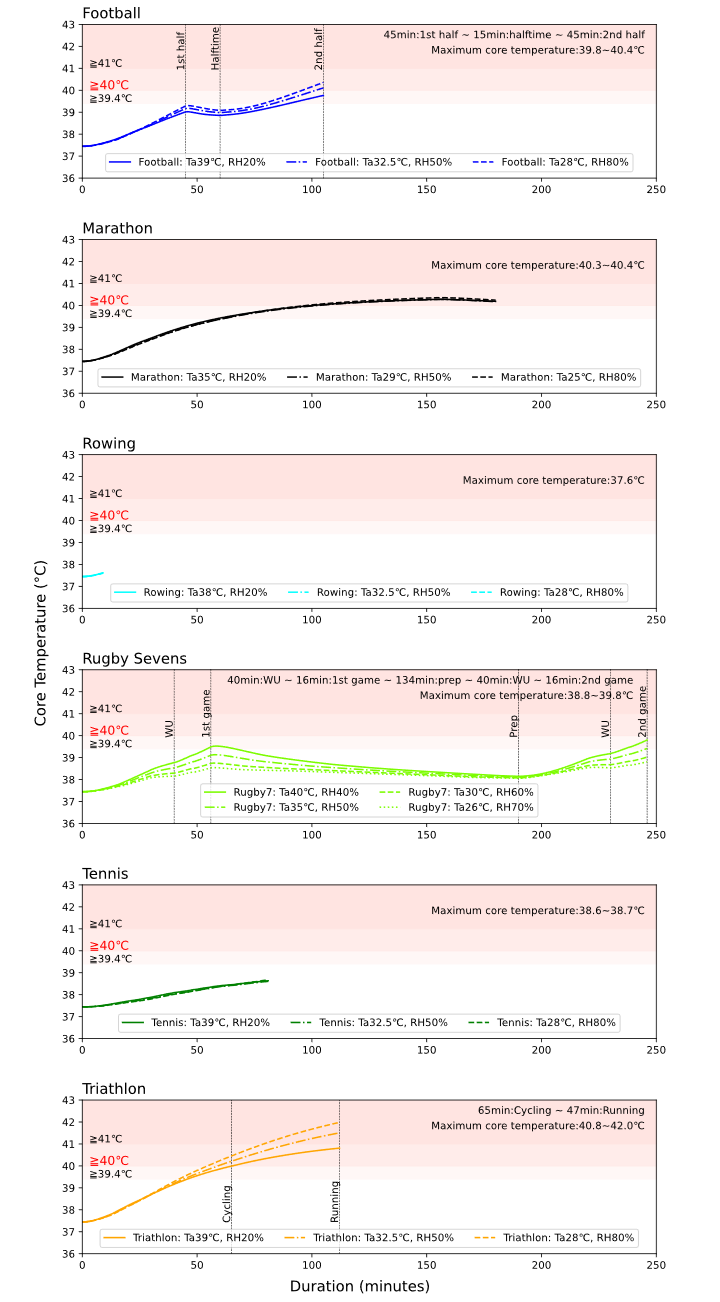


**Supplementary Fig. 54** Predicted core temperature in six sports at the upper thresholds of the thermal safety guidelines (97.5th percentile value)


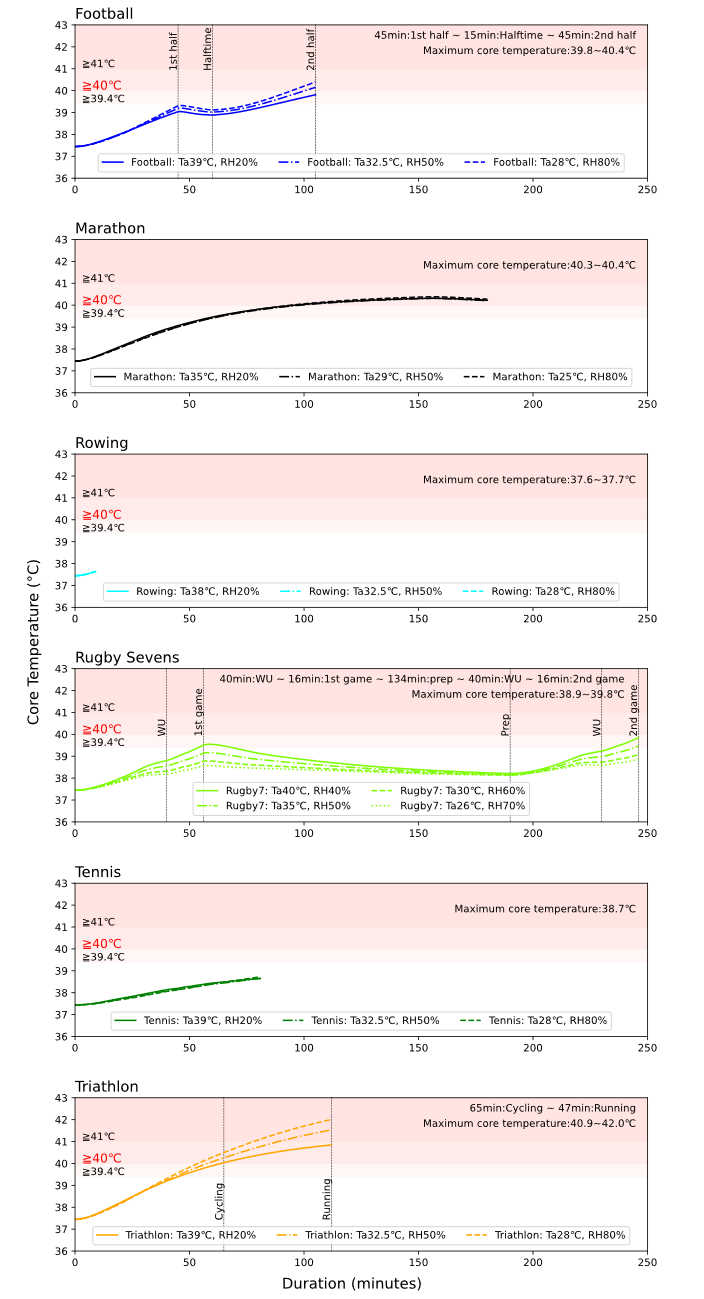


**Supplementary Fig. 55** Predicted core temperature in six sports at the upper thresholds of the thermal safety guidelines (99th percentile value)

1. Revised main parameter settings for three target sports in JOS-3 (90th percentile value)

| Sports  (guidelines’ upper thresholds) | T_a_ (℃) | RH (%) | T_r_ (℃) | v (m/s) | M (ml・　kg^-1^・min^-1^) | D (min) | W (kg) | H (m) | Age (year) | Sex |
| --- | --- | --- | --- | --- | --- | --- | --- | --- | --- | --- |
| Football  (WBGT32℃) | 40  (HT:15) | 20  (HT:50) | 60  (HT:15) | 1  (HT: 5) | Game: 35  (SD:3.5)  HT: 8.8  (SD:0.9) | 105(1^st^ half:45, HT:15,  2^nd^ half:45) | 75  (SD:10) | 1.75  (SD:0.1) | 35  (SD:10) | Male |
|  | 32.5  (HT:15) | 50  (HT:50) | 52.5  (HT:15) |  |  |  |  |  |  |  |
|  | 28  (HT:15) | 80  (HT:50) | 48  (HT:15) |  |  |  |  |  |  |  |
| Marathon  (WBGT27℃) | 34 | 20 | 54 | 1 | 0-36km: 44  (SD:4.4)  36-42.195km: 38  (SD:3.8) | 210  (SD:30) |  |  |  |  |
|  | 28 | 50 | 48 |  |  |  |  |  |  |  |
|  | 24 | 80 | 44 |  |  |  |  |  |  |  |
| Triathlon  (WBGT26℃) | 32 | 20 | 52 | 1 | Cycling: 45.5  (SD:4.6)  Running: 42  (SD:4.2) | 119  (SD:7) |  |  |  |  |
|  | 27 | 50 | 47 |  |  |  |  |  |  |  |
|  | 23 | 80 | 43 |  |  |  |  |  |  |  |

(Abbreviations – D: duration, H: height, HT: halftime, RH: relative humidity, M: metabolic rate, SD: standard deviation, Ta: ambient temperature, Tr: mean radiative temperature, WBGT: Wet Bulb Globe Temperature, v: wind speed, W: weight, WU: warm-up)

**Supplementary Fig. 56** Predicted core temperature in three sports at the modified upper thresholds of the thermal safety guidelines (90th percentile value)

1. Revised main parameter settings for three target sports in JOS-3 (95th percentile value)

| Sports  (guidelines’ upper thresholds) | T_a_ (℃) | RH (%) | T_r_ (℃) | v (m/s) | M (ml・　kg^-1^・min^-1^) | D (min) | W (kg) | H (m) | Age (year) | Sex |
| --- | --- | --- | --- | --- | --- | --- | --- | --- | --- | --- |
| Football  (WBGT31℃) | 38  (HT:15) | 20  (HT:50) | 58  (HT:15) | 1  (HT: 5) | Game: 35(SD:3.5)  HT: 8.8(SD:0.9) | 105(1^st^ half:45, HT:15,  2^nd^ half:45) | 75  (SD:10) | 1.75  (SD:0.1) | 35  (SD:10) | Male |
|  | 32  (HT:15) | 50  (HT:50) | 52  (HT:15) |  |  |  |  |  |  |  |
|  | 27.5  (HT:15) | 80  (HT:50) | 47.5  (HT:15) |  |  |  |  |  |  |  |
| Marathon  (WBGT26℃) | 32 | 20 | 52 | 1 | 0-36km: 44(SD:4.4)  36-42.195km: 38(SD:3.8) | 210(SD:30) |  |  |  |  |
|  | 27 | 50 | 47 |  |  |  |  |  |  |  |
|  | 23 | 80 | 43 |  |  |  |  |  |  |  |
| Triathlon  (WBGT25℃) | 31 | 20 | 51 | 1 | Cycling: 45.5(SD:4.6)  Running: 42(SD:4.2) | 119(SD:7) |  |  |  |  |
|  | 26 | 50 | 46 |  |  |  |  |  |  |  |
|  | 22 | 80 | 42 |  |  |  |  |  |  |  |

(Abbreviations – D: duration, H: height, HT: halftime, RH: relative humidity, M: metabolic rate, SD: standard deviation, Ta: ambient temperature, Tr: mean radiative temperature, WBGT: Wet Bulb Globe Temperature, v: wind speed, W: weight, WU: warm-up)

**Supplementary Fig. 57** Predicted core temperature in three sports at the modified upper thresholds of the thermal safety guidelines (95th percentile value)

1. Revised main parameter settings for three target sports in JOS-3 (97.5th percentile value)

| Sports  (guidelines’ upper thresholds) | T_a_ (℃) | RH (%) | T_r_ (℃) | v (m/s) | M (ml・　kg^-1^・min^-1^) | D (min) | W (kg) | H (m) | Age (year) | Sex |
| --- | --- | --- | --- | --- | --- | --- | --- | --- | --- | --- |
| Football  (WBGT30℃) | 37  (HT:15) | 20  (HT:50) | 57  (HT:15) | 1  (HT: 5) | Game: 35  (SD:3.5)  HT: 8.8  (SD:0.9) | 105(1^st^ half:45, HT:15,  2^nd^ half:45) | 75  (SD:10) | 1.75  (SD:0.1) | 35  (SD:10) | Male |
|  | 31  (HT:15) | 50  (HT:50) | 51  (HT:15) |  |  |  |  |  |  |  |
|  | 27  (HT:15) | 80  (HT:50) | 47  (HT:15) |  |  |  |  |  |  |  |
| Marathon  (WBGT25℃) | 31 | 20 | 51 | 1 | 0-36km: 44(SD:4.4)  36-42.195km: 38(SD:3.8) | 210(SD:30) |  |  |  |  |
|  | 26 | 50 | 46 |  |  |  |  |  |  |  |
|  | 22 | 80 | 42 |  |  |  |  |  |  |  |
| Triathlon  (WBGT24℃) | 30 | 20 | 50 | 1 | Cycling: 45.5(SD:4.6)  Running: 42(SD:4.2) | 119(SD:7) |  |  |  |  |
|  | 25 | 50 | 45 |  |  |  |  |  |  |  |
|  | 21 | 80 | 41 |  |  |  |  |  |  |  |

(Abbreviations – D: duration, H: height, HT: halftime, RH: relative humidity, M: metabolic rate, SD: standard deviation, Ta: ambient temperature, Tr: mean radiative temperature, WBGT: Wet Bulb Globe Temperature, v: wind speed, W: weight, WU: warm-up)

**Supplementary Fig. 58** Predicted core temperature in three sports at the modified upper thresholds of the thermal safety guidelines (97.5th percentile value)

1. Revised main parameter settings for three target sports in JOS-3 (99th percentile value)

| Sports  (guidelines’ upper thresholds) | T_a_ (℃) | RH (%) | T_r_ (℃) | v (m/s) | M (ml・kg^-1^・min^-1^) | D (min) | W (kg) | H (m) | Age (year) | Sex |
| --- | --- | --- | --- | --- | --- | --- | --- | --- | --- | --- |
| Football  (WBGT30℃) | 37  (HT:15) | 20  (HT:50) | 57  (HT:15) | 1  (HT: 5) | Game: 35(SD:3.5)  HT: 8.8(SD:0.9) | 105(  1^st^ half:45, HT:15,  2^nd^ half:45) | 75  (SD:10) | 1.75  (SD:0.1) | 35  (SD:10) | Male |
|  | 31  (HT:15) | 50  (HT:50) | 51  (HT:15) |  |  |  |  |  |  |  |
|  | 27  (HT:15) | 80  (HT:50) | 47  (HT:15) |  |  |  |  |  |  |  |
| Marathon  (WBGT24℃) | 30 | 20 | 50 | 1 | 0-36km: 44(SD:4.4)  36-42.195km: 38(SD:3.8) | 210(SD:30) |  |  |  |  |
|  | 25 | 50 | 45 |  |  |  |  |  |  |  |
|  | 21 | 80 | 41 |  |  |  |  |  |  |  |
| Triathlon  (WBGT24℃) | 30 | 20 | 50 | 1 | Cycling: 45.5(SD:4.6)  Running: 42(SD:4.2) | 119(SD:7) |  |  |  |  |
|  | 25 | 50 | 45 |  |  |  |  |  |  |  |
|  | 21 | 80 | 41 |  |  |  |  |  |  |  |

(Abbreviations – D: duration, H: height, HT: halftime, RH: relative humidity, M: metabolic rate, SD: standard deviation, Ta: ambient temperature, Tr: mean radiative temperature, WBGT: Wet Bulb Globe Temperature, v: wind speed, W: weight, WU: warm-up)

**Supplementary Fig. 59** Predicted core temperature in three sports at the modified upper thresholds of the thermal safety guidelines (99th percentile value)

1. Revised main parameter settings for three target sports in JOS-3 (99.7th percentile value)

| Sports  (guidelines’ upper thresholds) | T_a_ (℃) | RH (%) | T_r_ (℃) | v (m/s) | M (ml・kg^-1^・min^-1^) | D (min) | W (kg) | H (m) | Age (year) | Sex |
| --- | --- | --- | --- | --- | --- | --- | --- | --- | --- | --- |
| Football  (WBGT29℃) | 36  (HT:15) | 20  (HT:50) | 56  (HT:15) | 1  (HT: 5) | Game: 35(SD:3.5)  HT: 8.8(SD:0.9) | 105(1^st^ half:45, HT:15,  2^nd^ half:45) | 75  (SD:10) | 1.75  (SD:0.1) | 35  (SD:10) | Male |
|  | 30  (HT:15) | 50  (HT:50) | 50  (HT:15) |  |  |  |  |  |  |  |
|  | 26  (HT:15) | 80  (HT:50) | 46  (HT:15) |  |  |  |  |  |  |  |
| Marathon  (WBGT24℃) | 30 | 20 | 50 | 1 | 0-36km: 44(SD:4.4)  36-42.195km: 38(SD:3.8) | 210(SD:30) |  |  |  |  |
|  | 25 | 50 | 45 |  |  |  |  |  |  |  |
|  | 21 | 80 | 41 |  |  |  |  |  |  |  |
| Triathlon  (WBGT23℃) | 28 | 20 | 48 | 1 | Cycling: 45.5(SD:4.6)  Running: 42(SD:4.2) | 119(SD:7) |  |  |  |  |
|  | 24 | 50 | 44 |  |  |  |  |  |  |  |
|  | 20 | 80 | 40 |  |  |  |  |  |  |  |

(Abbreviations – D: duration, H: height, HT: halftime, RH: relative humidity, M: metabolic rate, SD: standard deviation, Ta: ambient temperature, Tr: mean radiative temperature, WBGT: Wet Bulb Globe Temperature, v: wind speed, W: weight, WU: warm-up)

**Supplementary Fig. 60** Predicted core temperature in three sports at the modified upper thresholds of the thermal safety guidelines (99.7th percentile value)

1. Percentiles corresponding to a core temperature of 40℃, stratified according to WBGT and type of sport

| WBGT (℃) | Football | Marathon | Triathlon |
| --- | --- | --- | --- |
| >32 | <90th PCTL | <90th PCTL | <90th PCTL |
| 32 | 90th PCTL |  |  |
| 31 | 95th PCTL |  |  |
| 30 | 97.5th–99th PCTL |  |  |
| 29 | 99.7th PCTL |  |  |
| 28 | >99.7th PCTL |  |  |
| 27 |  | 90th PCTL |  |
| 26 |  | 95th PCTL | 90th PCTL |
| 25 |  | 97.5th–99th PCTL | 95th PCTL |
| 24 |  | 99.7th PCTL | 97.5th–99th PCTL |
| 23 |  | >99.7th PCTL | 99.7th PCTL |
| <23 |  |  | >99.7th PCTL |

(Abbreviations – PCTL: percentile, WBGT: Wet Bulb Globe Temperature)
